# Supplementary material for: Design of bacteriophage T4-based artificial viral vectors for human genome remodeling
Source: Nat Commun. 2023 May 30;14:2928. doi: 10.1038/s41467-023-38364-1 (PMC10229621; doi:10.1038/s41467-023-38364-1)
Supplement: Supplementary file 1 — Supplementary Information [file 41467_2023_38364_MOESM1_ESM.pdf]

Supplementary information

Design of Bacteriophage T4-based Artificial Viral Vectors for Human Genome Remodeling

Jingen Zhu, Himanshu Batra, Neeti Ananthaswamy, Marthandan Mahalingam, Pan Tao, Xiaorong Wu, Wenzheng Guo, Andrei Fokin, and Venigalla B. Rao

Supplemental Figures and Legends

Supplementary Fig. 1: Preparation of head nanoparticles, packaging assays, and quantification of packaged DNAs for assembly of various T4-AVVs described in Results.

Supplementary Fig. 2: T4-AVV formation, payload delivery, and comparison with AAV2.

Supplementary Fig. 3: T4-AVVs displayed with Soc- and Hoc-fused proteins and their delivery characterizations.

Supplementary Fig. 4: Purified Cas9-Soc fusion protein showing site-specific DNA cleavage activity comparable to WT Cas9 protein, and delivery of displayed gRNA-Cas9-Soc RNP complexes by T4-AVVs.

Supplementary Fig. 5: T4-AVV mediated genome editing, homologous recombination, and site-specific Cre-recombination.

Supplementary Fig. 6: siRNA and mRNA delivery by T4-AVVs in combination with gene expression and genome editing molecules.

Supplementary Fig. 7: Re-wiring the T4 capsid exterior and interior by CRISPR engineering and construction of super-acidic and protein-packaging recombinant phages.

Supplemental Tables

Supplemental Table 1: Confirmation of dystrophin protein expression in T4(*Dys*)-AVV transduced cells by mass spectrometry.

Supplemental Table 2: Cryo-EM data and refinement statistics of 9DE-T4 capsid.

Supplemental Notes

Supplemental Note 1: Sequences of DNA template, gRNA, and recombinant protein used in this study.

Supplemental Note 2: Full wwPDB EM validation report.

Supplemental Figures and Legends

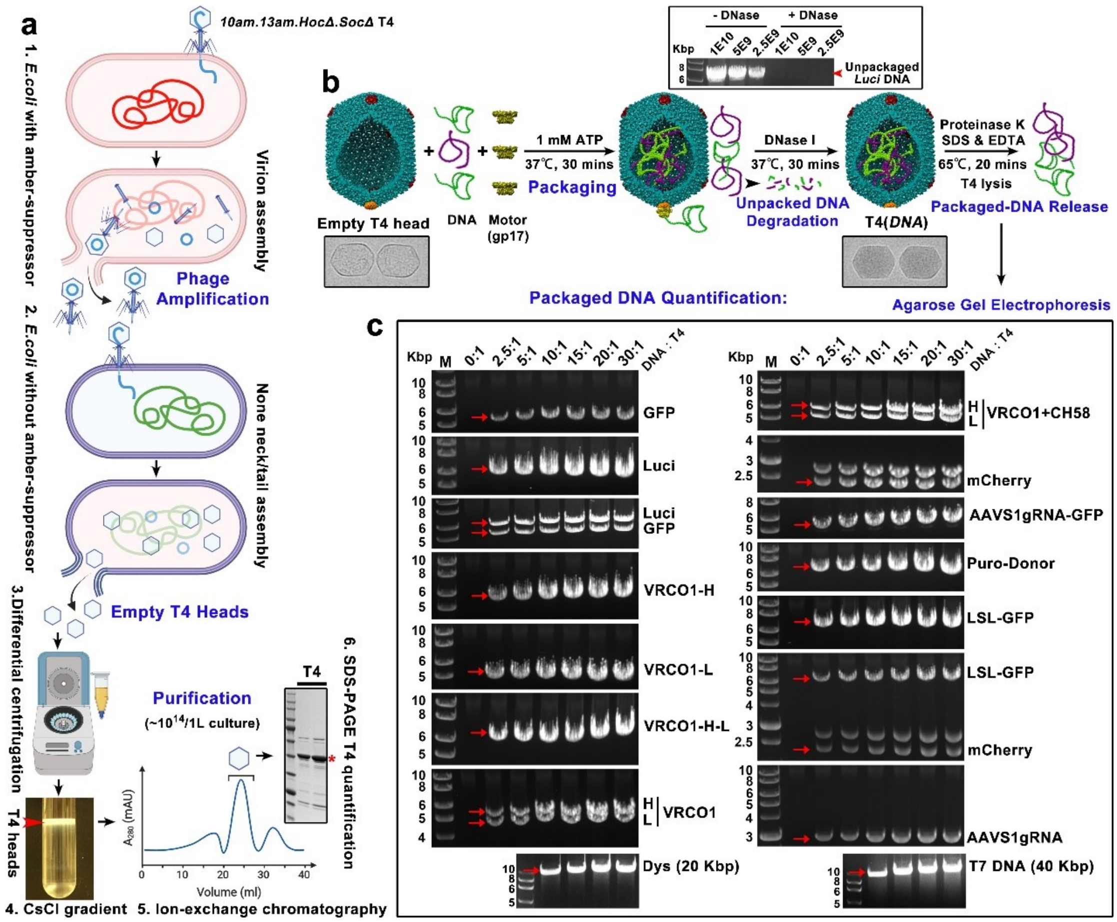

Supplementary Fig. 1: **Preparation of head nanoparticles, packaging assays, and quantification of packaged DNAs for assembly of various T4-AVVs described in Results**. **a** The workflow for production, purification, and quantification of *10am.13am.HocΔ.SocΔ* empty head particles. The amber mutations (TAG) in T4 gp10 (baseplate) and gp13 (neck) were read-through by *E.coli* containing amber suppressor (e.g., *E. coli* B40 *sup*[1], serine suppressor) producing *10am.13am.HocΔ.SocΔ* T4 phages. When these phages infect *E.coli* lacking amber suppressor (e.g., *E. coli* P301), empty T4 heads are produced. The heads were isolated by differential centrifugation followed by CsCl density gradient centrifugation and anion-exchange chromatography (diethylaminoethyl (DEAE)-Sepharose or quaternary ammonium (Q)-Sepharose). The purified head particles were quantified by SDS-PAGE, using the intensity of the major capsid protein gp23* (red star, 48.7kDa, 930 copies per head, ~0.75 μg gp23 per 1 x 10^{10} particles). Approximately 1 x 10^{14} head particles were produced from one liter of *E.coli* lab-culture. **b** The workflow for packaging assays and packaged DNA quantification. The packaging reaction mixture contained purified heads, purified gp17 motor protein, DNA, and ATP. After incubation at 37°C for 30 mins, the reactions were terminated by the addition of excess DNase I or Benzonase to digest the unpackaged DNA, while the packaged DNA was resistant. The encapsidated DNA molecules were then released by digesting the capsid with proteinase K and analyzed by agarose gel electrophoresis. The cryo-EM

images of empty heads and DNA-packaged heads are shown below the schematic. The top box shows the unpackaged *Luci* DNA in the T4 packaging reaction with and without DNase treatment. **c** The linearized DNAs were incubated with T4 heads at increasing DNA molecules-to-head particle ratios as indicated at the top of the panels. Maximum packaging occurred at a ratio of 15-20:1. The red arrows indicate the positions of the packaged DNA bands. Panel a (except the CsCl tube and SDS-PAGE images) was created by BioRender.

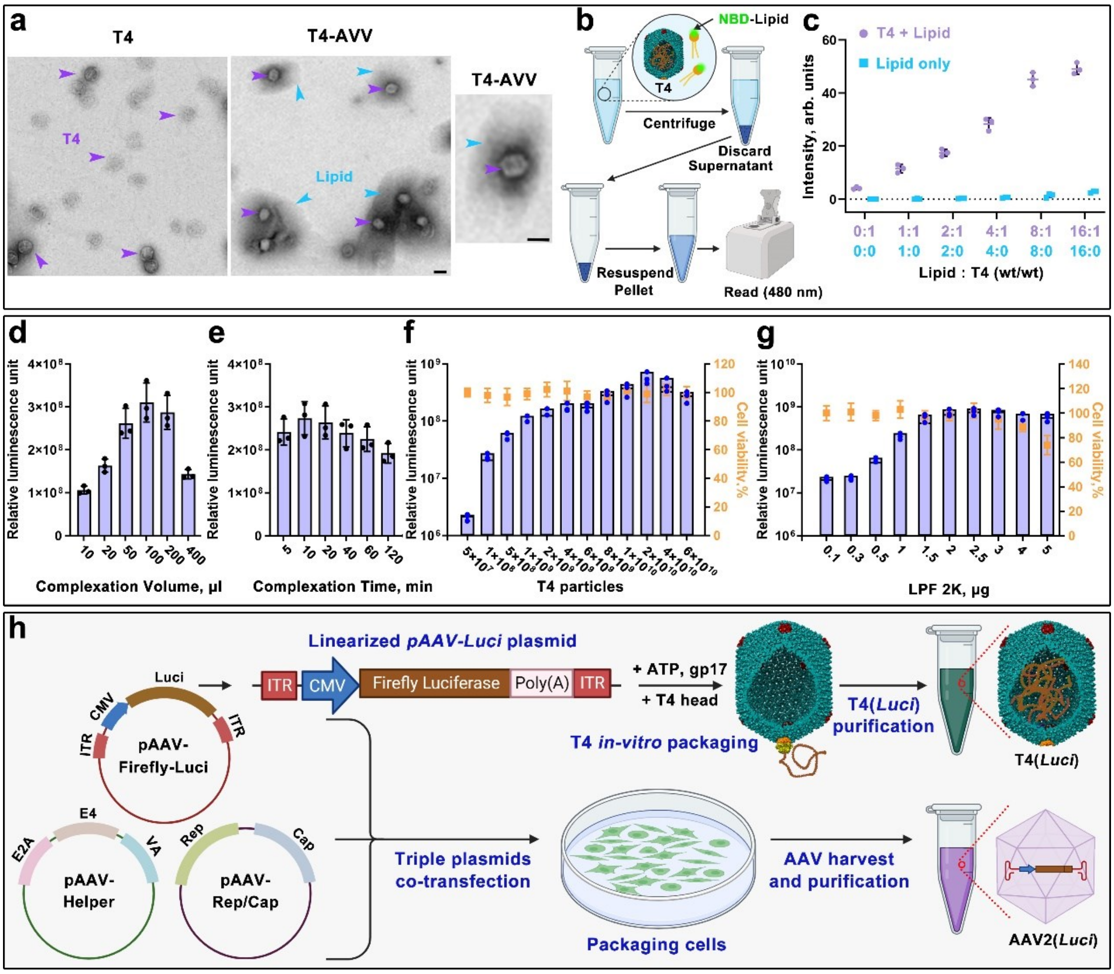

Supplementary Fig. 2: T4-AVV formation, payload delivery, and comparison with AAV2. **a** Negative stain electron-microscopy (EM) images of T4 control and T4-AVVs. The fuzzy lipid coat and T4 head are labeled with cyan and blue arrowheads, respectively. Bar = 100 nm. **b** The schematic workflow of quantitative analysis of lipid coating on T4 head. Briefly, T4 head particles were first incubated with cationic lipid molecules labeled with nitrobenzoxadiazole (NBD, green) at room temperature for 45 minutes. The resulting mixture was then subjected to 7,000 g centrifugation for 30 minutes, after which the pellet was washed and resuspended with 1× PBS. The fluorescent T4-AVVs were subsequently quantified using a spectrophotometer with an excitation wavelength of 480 nm. NBD-labeled cationic lipids were used as the control for this analysis. **c** Quantitative measurement of lipid coating on T4 head (n = 3). Different amounts of lipids were incubated with T4 as indicated at the bottom. The amount of lipids alone in the pellet was minimal, whereas the fluorescence intensity of T4-AVVs in the pellet increased with an increase in

the amount of lipids. arb. units, arbitrary units. **d** Effect of T4 and lipid complexation volume on T4(*Luci*)-AVV delivery efficiency (n = 3). **e** Effect of T4 and lipid complexation time on T4(*Luci*)-AVV delivery efficiency (n = 3). **f** Optimal ratio of T4 particles to LPF2K concentration (2 μg) on delivery efficiency and cell viability (n = 3). Luminescence activity (histogram) and cell viability (orange squares) assays were performed at 48 hr post-transduction. Quantification of the number of viable cells in culture was based on the determination of ATP by luminescent cell viability assay, which directly correlates with the presence of metabolically active cells. Percent viability was calculated in comparison with the untreated control. The T4-AVVs show no toxicity at a MOI of 10^5 where maximum signal readout of transgene expression was obtained. **g** Optimization of the LPF2K amount for complexing with 2 x 10^{10} T4(*Luci*) (histogram) and relative cell viability (orange squares) (n = 3). As low as 1.5 μg lipids are sufficient for complexing 2 x 10^{10} T4(*Luci*) particles for maximum transgene expression. Much excessive lipids lead to reduced cell viability, such as 3-fold more lipids than needed. **h** Schematic of T4(*Luci*) and AAV2(*Luci*) production. The recombinant AAV2 was produced by the standard triple transfection protocol using the adenoviral helper plasmid (pAAV-Helper), a rep/cap plasmid expressing Rep and Cap proteins (pAAV-Rep/Cap), and an inverted terminal repeat (ITR) plasmid containing firefly luciferase gene (pAAV-Luciferase). The single-stranded genome packaged inside the AAV capsid is: *AAV2ITR-CMV enhancer and promoter-fireflyLuci-hGH poly(A)*. For head-to-head comparison, the same-sequence genome in the form of double-stranded linearized plasmid was also packaged into T4, to ~8 molecules per T4 head particle. Values represent mean with SD. Panels b and h were created by BioRender.

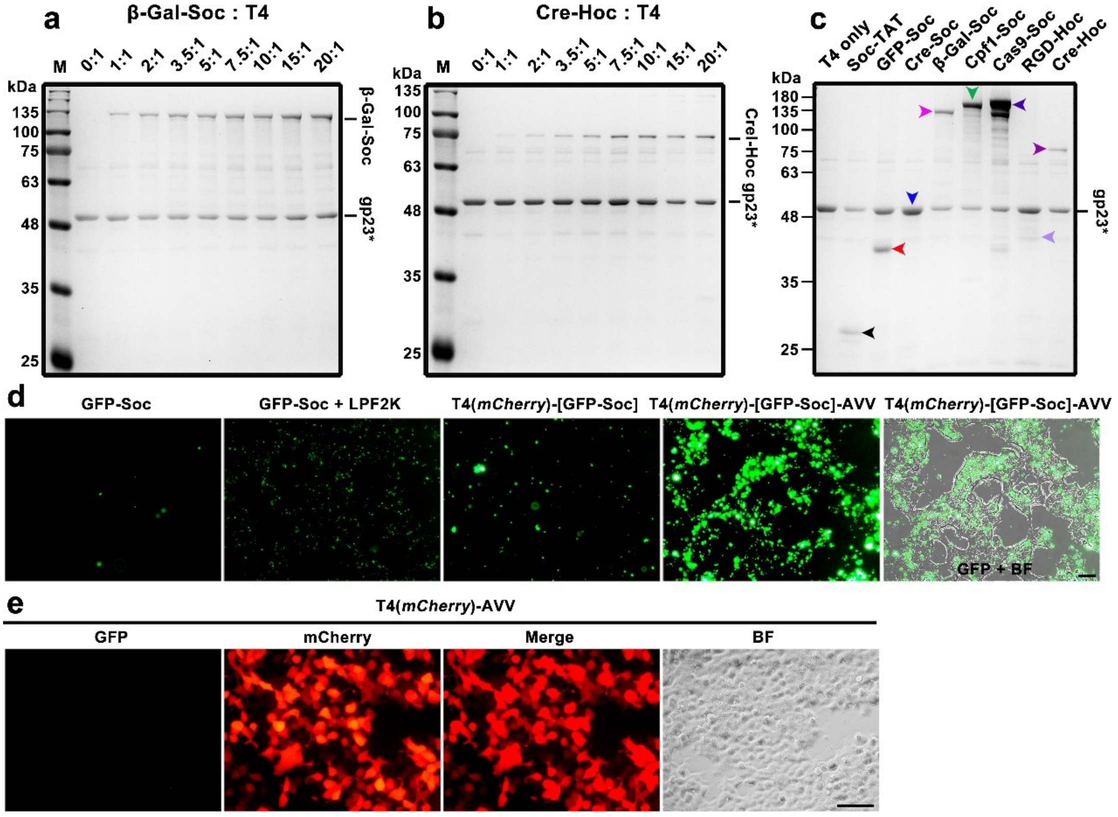

Supplementary Fig. 3: T4-AVVs displayed with Soc- and Hoc-fused proteins and their delivery characterizations. a to c Display of Soc- and Hoc-fused proteins on T4 capsids. Soc-TAT, GFP-Soc, Cre-Soc, β-Gal-Soc, Cpf1-Soc, Cas9-Soc, RGD-Hoc, and Cre-Hoc were overexpressed, purified, and incubated with T4 heads at increasing ratios of protein molecules to Soc- or Hoc-binding sites. The arrowheads show the positions of various displayed proteins. The major capsid protein gp23* was used as an internal control (~0.75 μg gp23 per 1 x 10^{10} T4 particles, 930 copies per capsid) to determine the copy number of the respective displayed protein per capsid. **(a)** and **(b)** show binding patterns at different ratios, indicating that saturation reached at ~15-20:1 ratio. **(c)** shows binding characteristics of a variety of proteins differing in size, charge, oligomeric nature, and function at 20:1 ratio. **d** Representative GFP fluorescent images of cells at 3 hr after treatment with GFP-Soc, GFP-Soc + LPF2K (mixture), T4(*mCherry*)-[GFP-Soc] (no lipids), or T4(*mCherry*)-[GFP-Soc]-AVVs. The rightmost panel shows the merged image of GFP signal and bright field (BF), demonstrating that the displayed GFP protein efficiently attached to the cell surface at 3 hr after exposure. Bar = 50 μm. **e** Control experiment (related to Fig. **3e**) showing *mCherry* DNA delivery using T4(*mCherry*)-AVVs where only mCherry expression was observed. Bar = 50 μm.

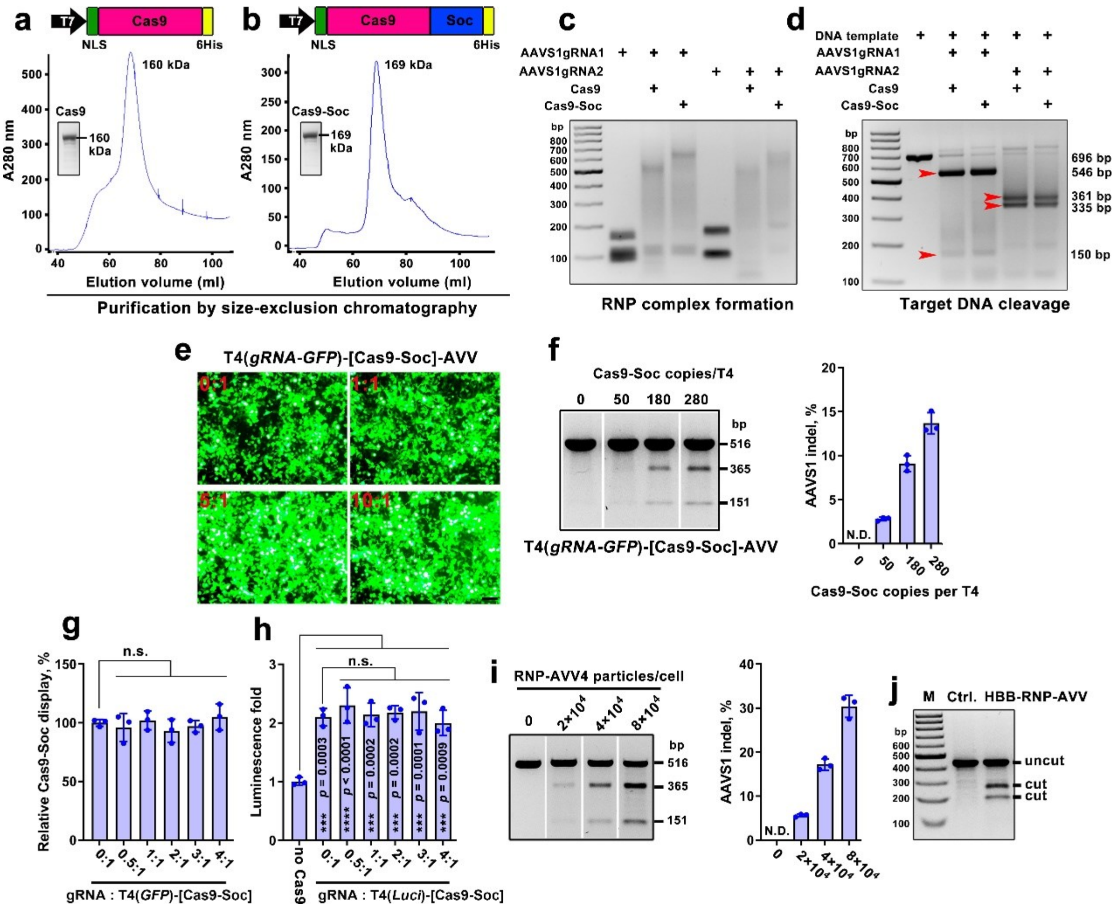

Supplementary Fig. 4: Purified Cas9-Soc fusion protein showing site-specific DNA cleavage activity comparable to WT Cas9 protein, and delivery of displayed gRNA-Cas9-Soc RNP complexes by T4-AVVs. a and b show the expression cassettes and size-exclusion chromatography profiles of Cas9 **(a)** and Cas9-Soc **(b)**, respectively. Both

Cas9 and Cas9-Soc also contain an N-terminal SV40 nuclear localization signal (NLS) (NLS allows the transport of Cas9 into the nucleus) and a C-terminal hexa-His tag. The fusion proteins were over-expressed in *E. coli* under the control of the T7 promoter and purified by HisTrap affinity chromatography and size-exclusion chromatography. The purified Cas9 and Cas9-Soc exist predominantly as monomers, as evident from the molecular size determined using the respective elution volumes. The purified proteins showed a single protein band, as analyzed by SDS-PAGE and shown in the inserts. **c** Comparison of Cas9 and Cas9-Soc binding to AAVS1 gRNA1 or gRNA2 by agarose gel retardation assay. **d** Cas9 and Cas9-Soc showed comparable levels of DNA cleavage activity at the specific gRNA targeted sites *in vitro*. Red arrowheads point to the respective cleavage products. **e** Fluorescence images showing enhanced GFP reporter expression with increasing copy number of displayed Cas9-Soc on T4(*gRNA-GFP*)-AVVs, 48 hrs post-transduction. Bar = 50 µm. **f** Agarose gel showing the editing of endogenous AAVS1 locus following AVV-mediated delivery of Cas9 protein and gRNA-expressing plasmid DNA (T4-AVV2 in Fig. **4b**). Indel mutations were detected by the T7E1 assay three days after transduction (left panel) and their efficiency at various copy numbers of displayed Cas9 was quantified and shown in the right panel (n = 3). **g** Control experiment showing that binding of gRNA to T4(*GFP*)–[Cas9-Soc] did not cause any dissociation of the displayed Cas9-Soc on T4 (n = 3). **h** Control experiment showing that *luciferase* transgene transduction was not affected by the amount of gRNA bound to T4(*Luci*)-[RNP-Soc]-AVVs at increasing gRNA ratios (0:1 to 4:1) (n = 3). The luciferase activity is presented as the fold change compared to Cas9-lacking AVVs. **i** T7E1 assay (left panel) and quantification (right panel) show genome editing at the AAVS1 locus by T4-RNP-AVVs delivered at different MOIs (AVV nanoparticles to cells) (n = 3). **j** T7E1 assay shows HBB gene disruption mediated by T4(*GFP*)-[HBB-RNP-Soc]-AVVs. Values represent mean with SD. ***$P < 0.001$, ****$P < 0.0001$, n.s., not significant, one-way ANOVA test.

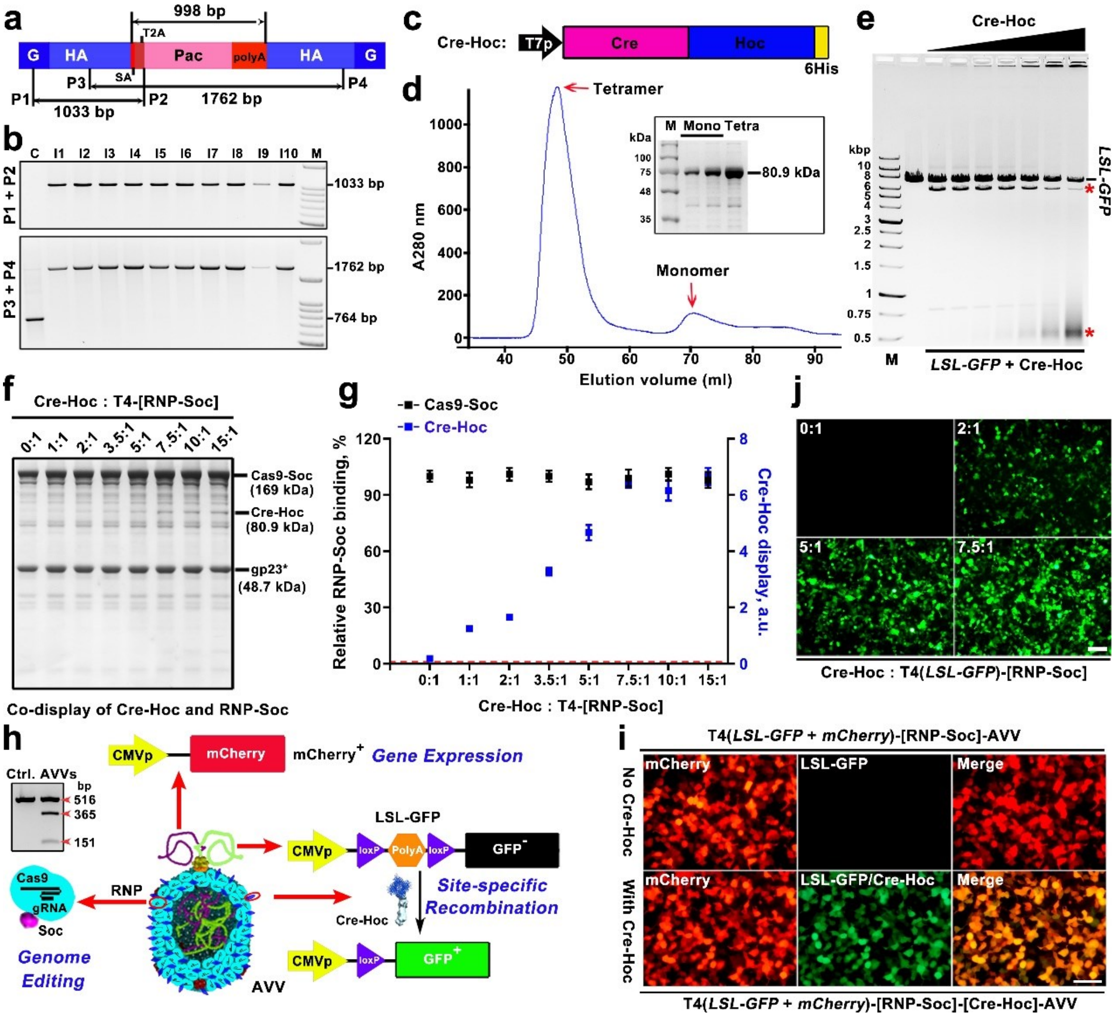

Supplementary Fig. 5: T4-AVV mediated genome editing, homologous recombination, and site-specific Cre-recombination. a Schematic showing the locations of PCR primer sets (P1&P2, P3&P4) for detecting targeted insertions resulted from T4(*Pac*-donor)-[RNP-Soc]-AVVs transduction. SA, splice acceptor site. T2A, 2A cleavable peptide from *Thosea asigna* virus capsid protein. **b** PCR assay of the AAVS1 gene was performed on the DNA isolated from single cell clones of T4(*Pac-donor*)-[RNP-Soc]-AVVs treated cells. Ten independent single cell puromycin-resistant clones (I1 to I10) were analyzed using each primer set. **c** Schematic of Cre-Hoc expression cassette. Hoc was fused to the C-terminus of Cre with a hexa-His tag and over-expressed in *E. coli* under the control of the T7 promoter. **d** Size-exclusion chromatography profile of Cre-Hoc protein. The red arrows indicate the eluted Cre-Hoc tetramer and monomer. The hatched box inside shows the SDS-PAGE analysis of Cre-Hoc tetramer and monomer. Both tetramer and monomer were active for phage display and site-specific recombination. **e** Agarose gel showing site-specific recombination by Cre-Hoc of *LoxP-STOP-LoxP-GFP (LSL-GFP)* DNA substrate containing two *loxP* sites. Red stars point to the recombinant products of Cre recombinase. **f** Co-display of Cas9-Soc at constant 5:1 ratio (Cas9-Soc molecules to Soc binding sites) and Cre-Hoc at increasing ratios (Cre-Hoc molecules to Hoc binding sites), as determined by SDS-PAGE analysis. **g** Both Cas9-Soc and Cre-Hoc bound to the same capsid and increasing Cre-Hoc display did not affect Cas9-Soc binding. Values

represent mean with SD (*n* = 3). **h** Schematic of site-specific recombination by delivery of T4(*LoxP-STOP-LoxP-GFP* + *mCherry*)-[RNP-Soc]-[Cre-Hoc]-AVVs. **i** *LoxP-STOP-LoxP-GFP* and *mCherry* DNAs were co-delivered and co-expressed in each cell, with the GFP expression occurring following recombination by co-delivered Cre protein, all through the same payload incorporated into the T4-AVVs. Bar = 50 μm. **j** Representative GFP expression images following transduction of 293 cells with T4(*LoxP-STOP-LoxP-GFP*)-[RNP-Soc]-[Cre-Hoc]-AVVs at increasing Cre-Hoc display ratio. Bar = 50 μm.

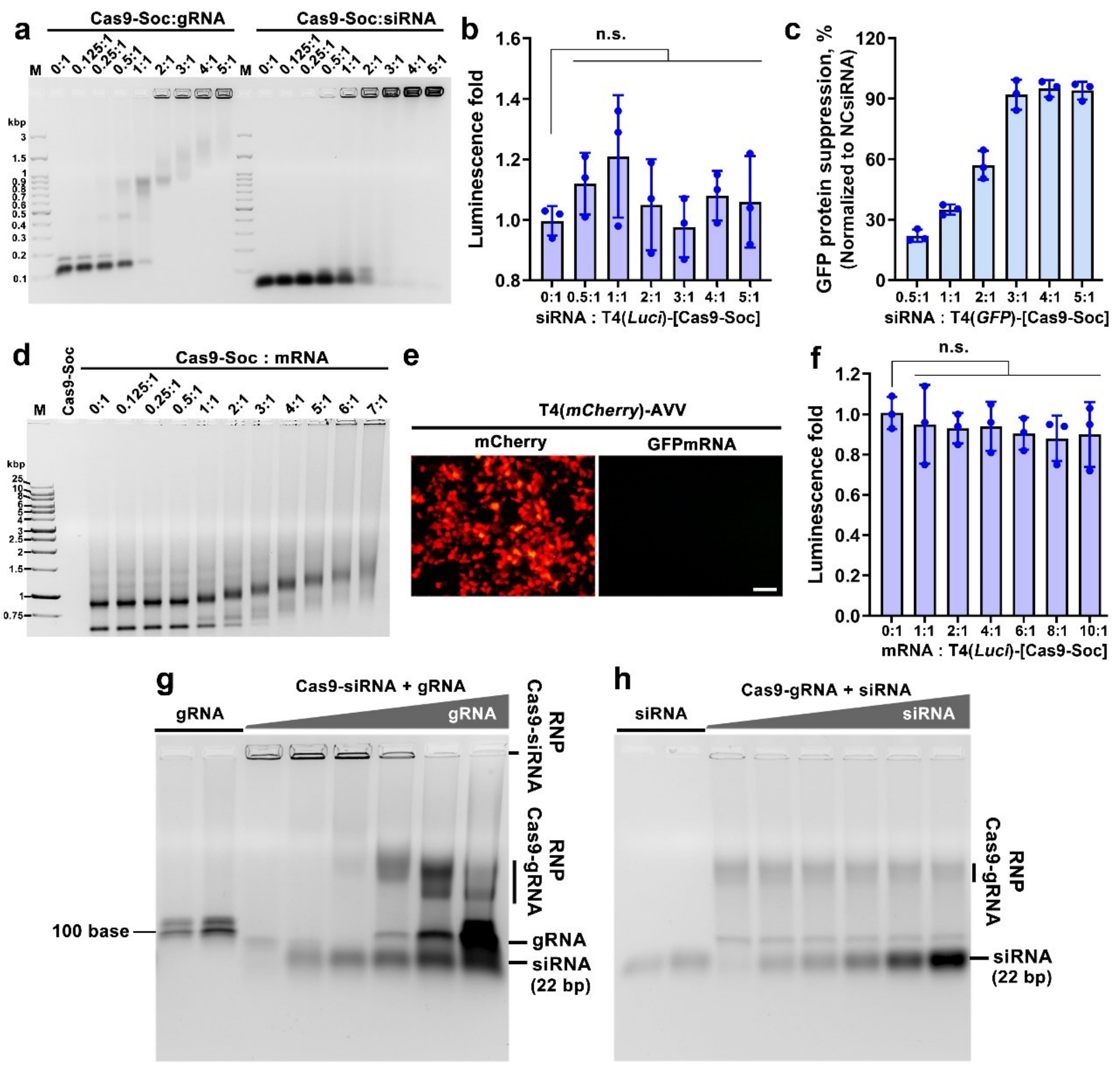

Supplementary Fig. 6: siRNA and mRNA delivery by T4-AVVs in combination with gene expression and genome editing molecules. **a** Electrophoretic mobility shift (gel retardation) assay to determine gRNA or siRNA binding to Cas9-Soc. A constant amount of gRNA or siRNA was mixed with various molar ratios (0:1 to 5:1) of Cas9-Soc molecules for 1 hr at room temperature and then analyzed by agarose gel electrophoresis. The gRNA-Cas9-Soc or siRNA-Cas9-Soc complexes were retarded in mobility or remained in the loading well. **b** Effect of siRNA:T4(*Luci*)–[Cas9-Soc] ratios on T4-AVVs' delivery efficiency as determined by luciferase activity of T4(*Luci*)-[siRNA-Cas9-Soc]-AVVs when compared to control T4(*Luci*)-[Cas9-Soc]-AVVs lacking siRNA and presented as the fold change (n = 3). The amount of siRNA displayed did not affect the delivery efficiency of T4-AVVs. **c**

Dose-dependent silencing of GFP expression with increasing amounts of displayed GFPsiRNA (n = 3). **d** Electrophoretic mobility shift assay showing the binding of mRNA to Cas9-Soc protein at increasing ratios of Cas9-Soc molecules to mRNA (0:1 to 7:1). **e** Delivery and expression of T4(*mCherry*)-AVVs lacking Cas9 was tested as another control (related to Fig. **5h**). T4(*mCherry*)-AVVs were prepared similar to T4(*mCherry*)-[mRNA-Cas9-Soc]-AVVs but without the displayed Cas9-Soc. No significant binding of GFPmRNA to T4(*mCherry*) capsids or green fluorescence signal in transduced cells was observed without Cas9-Soc display. Bar = 50 µm. **f** Increasing the amount of the displayed mRNA did not affect on the co-delivery efficiency of T4(*Luci*)-[mRNA-Cas9-Soc]-AVVs. Luciferase expression was compared with the control AVVs lacking mRNA display and presented as the fold change (n = 3). **g and h** Electrophoretic mobility shift assays showing that gRNA can replace bound siRNA in the Cas9-siRNA complex **(g)**, whereas siRNA cannot replace gRNA in the Cas9-gRNA complex **(h)**. Note the Cas9-siRNA complexes in the wells and the Cas9-gRNA complexes in the middle of the gel, as labeled. Values represent mean with SD. n.s., not significant, one-way ANOVA test.

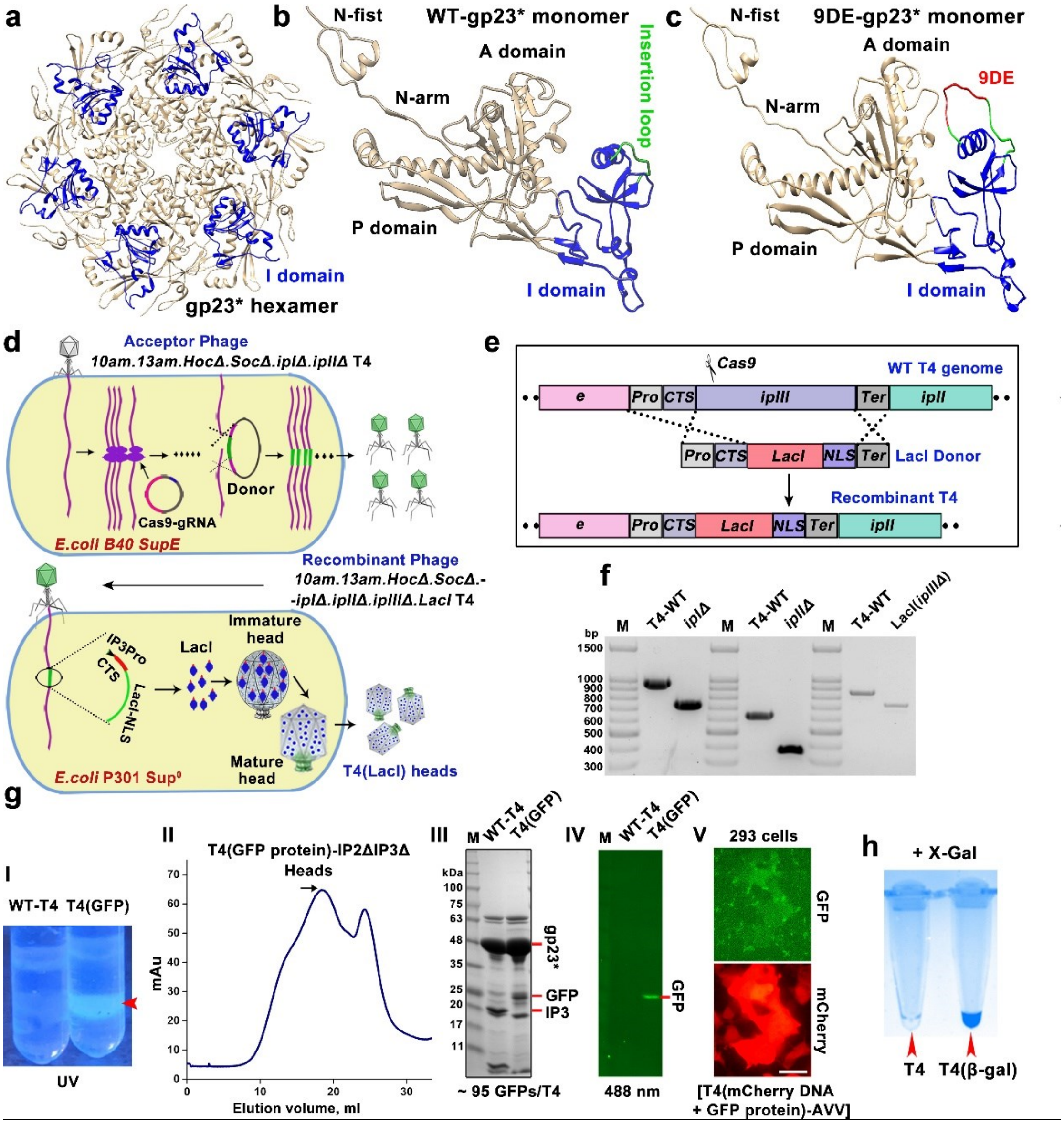

Supplementary Fig. 7: Re-wiring the T4 capsid exterior and interior by CRISPR engineering and construction of super-acidic and protein-packaging recombinant phages. a The cryo-EM structures of WT gp23* hexamer. The I domains are shown in blue color. **b and c** The cryo-EM structure of WT-gp23* **(b)** and 9DE-gp23* **(c)** monomer (A domain, axial domain; P domain, peripheral domain; I domain, insertion domain). The I domain insertion loop and the 9DE loop are shown in green and red colors, respectively. **d** Schematic of T4(LacI protein) head production. Top: CRISPR-mediated *LacI-NLS* gene insertion (*ipIII* replacement) in the background of *10am.13am.HocΔ.SocΔ.ipIΔ.ipIIΔ* T4 phage (acceptor phage). The acceptor phage was constructed by deleting *ipI* and *ipII* genes in the background of *10am.13am.HocΔ.SocΔ* T4 phage using a similar strategy. Bottom: production of LacI-NLS protein-packaged T4 heads by infecting *E. coli* containing no amber suppressor (P301) with recombinant *10am.13am.HocΔ.SocΔ.ipIΔ.ipIIΔ.ipIIIΔ.LacI* T4 phage. **e** Schematic of CRISPR-mediated *LacI-NLS* gene insertion (*ipIII* replacement). **f** Successive rounds of CRISPR-mediated T4 genome editing (*ipIΔ, ipIIΔ,* and *LacI-NLS* gene insertion with *ipIII* replacement) to create the mutant phages were confirmed by PCR. **g** Green fluorescence phage produced by packaging GFP protein in the capsid interior. **(I)** Control WT-T4 and T4(GFP protein) phages were purified by CsCl gradient centrifugation and exposed to UV light. **(II)** Anion-exchange chromatography profiles of T4(GFP) heads. **(III)** and **(IV)** WT-T4 and T4(GFP) heads were electrophoresed on an SDS polyacrylamide gel and stained with Coomassie blue **(III)** or exposed to UV light at 488nm **(IV)**. The positions of the protein bands corresponding to the respective CRISPR-introduced mutations are labeled. **(V)** T4(*mCherry DNA* + GFP protein)-AVVs deliver two fluorescence reporters into 293 cells, GFP as packaged protein and *mCherry* as packaged DNA. Bar = 10 μm. **h** Blue T4 heads, produced by packaging β-galactosidase tetramers in the capsid interior. The T4(β-gal protein) heads turn blue after the addition of the X-Gal substrate.

Supplemental Tables

Top 5 Reference Human Genes	Match Scores	
	T4 control	T4(*Dys*)-AVV
sp|P11532|**DMD** (426.5 kDa)	0	482
sp|Q14204|**DYHC1** (532.1 kDa)	320	294
sp|P78527|**PRKDC** (468.8 kDa)	208	191
sp|P12270|**TPR** (267.1 kDa)	111	167
sp|Q15149|**PLEC** (531.5 kDa)	143	140

Protein and Peptide Details for reference sp|P11532|DMD_HUMAN

MLWWEEVEDC YEREDVQKKT FTKWVNAQFS KFGKQHIENL FSDLQDGRRL LDLLEGLTGQ KLPKEK
GSTR VHALNNVNKA LRVLQNNNVD LVNIGSTDIV DGNHKLTLGL IWNIILHWQV KNVMKNIMAG L
QQTNSEKIL LSWVRQSTRN YPQVNVINFT TSWSDGLALN ALIHSHRPDL FDWNSVVCQQ SATQRLE
HAF NIARYQLGIE KLLDPEDVDT TYPDKKSILM YITSLFQVLP QQVSIEAIQE VEMLPRPPKV TKEEHF
QLHH QMHYSQQITV SLAQGYERTS SPKPRFKSYA YTQAAYVTTS DPTRSPFPSQ HLEAPEDKSF GSSL
MESEVN LDRYQTALEE VLSWLLSAED TLQAQGEISN DVEVVKDQFH THEGYMMDLT AHQGRVGNIL
QLGSKLIGTG KLSEDEETEV QEQMNLLNSR WECLRVASME KQSNLHRVLM DLQNQKLKEL NDWLT
KTEER TRKMEEEPLG PDLEDLKRQV QQHKVLQEDL EQEQVRVNSL THMVVVVDES SGDHATAALE E
QLKVLGDRW ANICRWTEDR WVLLQDILLK WQRLTEEQCL FSAWLSEKED AVNKIHTTGF KDQNEML
SSL QKLAVLKADL EKKKQSMGKL YSLKQDLLST LKNKSVTQKT EAWLDNFARC WDNLVQKLEK STAQ
ISQAVT TTQPSLTQTT VMETVTTVTT REQILVKHAQ EELPPPPPQK KRQITVDSEI RKRLDVDITE LHS
WITRSEA VLQSPEFAIF RKEGNFSDLK EKVNAIEREK AEKFRKLQDA SRSAQALVEQ MVNEGVNADS I
KQASEQLNS RWIEFCQLLS ERLNWLEYQN NIIAFYNQLQ QLEQMTTTAE NWLKIQPTTP SEPTAIKSQ
L KICKDEVNRL SDLQPQIERL KIQSIALKEK GQGPMFLDAD FVAFTNHFKQ VFSDVQAREK ELQTIFDT
LP PMRYQETMSA IRTWVQQSET KLSIPQLSVT DYEIMEQRLG ELQALQSSLQ EQQSGLYYLS TTVKE
MSKKA PSEISRKYQS EFEEIEGRWK KLSSQLVEHC QKLEEQMNKL RKIQNHIQTL KKWMAEVDVF LK
EEWPALGD SEILKKQLKQ CRLLVSDIQT IQPSLNSVNE GGQKIKNEAE PEFASRLETE LKELNTQWDH
MCQQVYARKE ALKGGLEKTV SLQKDLSEMH EWMTQAEEEY LERDFEYKTP DELQKAVEEM KRAKEE
AQQK EAKVKLLTES VNSVIAQAPP VAQEALKKEL ETLTTNYQWL CTRLNGKCKT LEEVWACWHE LLS
YLEKANK WLNEVEFKLK TTENIPGGAE EISEVLDSLE NLMRHSEDNP NQIRILAQTL TDGGVMDELI N
EELETFNSR WRELHEEAVR RQKLLEQSIQ SAQETEKSLH LIQESLTFID KQLAAYIADK VDAAQMPQEA
QKIQSDLTSH EISLEEMKKH NQGKEAAQRV LSQIDVAQKK LQDVSMKFRL FQKPANFEQR LQESKMI
LDE VKMHLPALET KSVEQEVVQS QLNHCVNLYK SLSEVKSEVE MVIKTGRQIV QKKQTENPKE LDER
VTALKL HYNELGAKVT ERKQQLEKCL KLSRKMRKEM NVLTEWLAAT DMELTKRSAV EGMPSNLDSE
VAWGKATQKE IEKQKVHLKS ITEVGEALKT VLGKKETLVE DKLSLLNSNW IAVTSRAEEW LNLLLEYQK
H METFDQNVDH ITKWIIQADT LLDESEKKKP QQKEDVLKRL KAELNDIRPK VDSTRDQAAN LMANR
GDHCR KLVEPQISEL NHRFAAISHR IKTGKASIPL KELEQFNSDI QKLLEPLEAE IQQGVNLKEE DFNKD
MNEDN EGTVKELLQR GDNLQQRITD ERKREEIKIK QQLLQTKHNA LKDLRSQRRK KALEISHQWY Q
YKRQADDLL KCLDDIEKKL ASLPEPRDER KIKEIDRELQ KKKEELNAVR RQAEGLSEDG AAMAVEPTQI
QLSKRWREIE SKFAQFRRLN FAQIHTVREE TMMVMTEDMP LEISYVPSTY LTEITHVSQA LLEVEQLLN
A PDLCAKDFED LFKQEESLKN IKDSLQQSSG RIDIIHSKKT AALQSATPVE RVKLQEALSQ LDFQWEKV
NK MYKDRQGRFD RSVEKWRRFH YDIKIFNQWL TEAEQFLRKT QIPENWEHAK YKWYLKELQD GIG
QRQTVVR TLNATGEEII QQSSKTDASI LQEKLGSLNL RWQEVCKQLS DRKKRLEEQK NILSEFQRDL NE
FVLWLEEA DNIASIPLEP GKEQQLKEKL EQVKLLVEEL PLRQGILKQL NETGGPVLVS APISPEEQDK LE

NKLKQTNL QWIKVSRALP EKQGEIEAQI KDLGQLEKKL EDLEEQLNHL LLWLSPIRNQ LEIYNQPNQE
GPFDVKETEI AVQAKQPDVE EILSKGQHLY KEKPATQPVK RKLEDLSSEW KAVNRLLQEL RAKQPDLA
PG LTTIGASPTQ TVTLVTQPVV TKETAISKLE MPSSLMLEVP ALADFNRAWT ELTDWLSLLD QVIKSQ
RVMV GDLEDINEMI IKQKATMQDL EQRRPQLEEL ITAAQNLKNK TSNQEARTII TDRIERIQNQ WDE
VQEHLQN RRQQLNEMLK DSTQWLEAKE EAEQVLGQAR AKLESWKEGP YTVDAIQKKI TETKQLAKD
L RQWQTNVDVA NDLALKLLRD YSADDTRKVH MITENINASW RSIHKRVSER EAALEETHRL LQQFP
LDLEK FLAWLTEAET TANVLQDATR KERLLEDSKG VKELMKQWQD LQGEIEAHTD VYHNLDENSQ KI
LRSLEGSD DAVLLQRRLD NMNFKWSELR KKSLNIRSHL EASSDQWKRL HLSLQELLVW LQLKDDELSR
QAPIGGDFPA VQKQNDVHRA FKRELKTKEP VIMSTLETVR IFLTEQPLEG LEKLYQEPRE LPPEERAQN
V TRLLRKQAEE VNTEWEKLNL HSADWQRKID ETLERLQELQ EATDELDLKL RQAEVIKGSW QPVGDL
LIDS LQDHLEKVKA LRGEIAPLKE NVSHVNDLAR QLTTLGIQLS PYNLSTLEDL NTRWKLLQVA VEDRV
RQLHE AHRDFGPASQ HFLSTSVQGP WERAISPNKV PYYINHETQT TCWDHPKMTE LYQSLADLNN V
RFSAYRTAM KLRRLQKALC LDLLSLSAAC DALDQHNLKQ NDQPMDILQI INCLTTIYDR LEQEHNNLVN
VPLCVDMCLN WLLNVYDTGR TGRIRVLSFK TGIISLCKAH LEDKYRYLFK QVASSTGFCD QRRLGLLLH
D SIQIPRQLGE VASFGGSNIE PSVRSCFQFA NNKPEIEAAL FLDWMRLEPQ SMVWLPVLHR VAAAET
AKHQ AKCNICKECP IIGFRYRSLK HFNYDICQSC FFSGRVAKGH KMHYPMVEYC TPTTSGEDVR DFAK
VLKNKF RTKRYFAKHP RMGYLPVQTV LEGDNMETPV TLINFWPVDS APASSPQLSH DDTHSRIEHY
ASRLAEMENS NGSYLNDSIS PNESIDDEHL LIQHYCQSLN QDSPLSQPRS PAQILISLES EERGELERIL A
DLEEENRNL QAEYDRLKQQ HEHKGLSPLP SPPEMMPTSP QSPRDAELIA EAKLLRQHKG RLEARMQI
LE DHNKQLESQL HRLRQLLEQP QAEAKVNGTT VSSPSTSLQR SDSSQPMLLR VVGSQTSDSM GEED
LLSPPQ DTSTGLEEVM EQLNNSFPSS RGRNTPGKPM REDTM

Supplemental Table 1: Confirmation of dystrophin protein expression in T4(*Dys*)-AVV transduced cells by mass spectrometry. The protein band at the expected size was excised from polyacrylamide gel and analyzed by mass spectrometry. The top table shows the top 5 detected human genes in the excised bands from T4(*Dys*)-AVV and T4 control transduced cells. The bottom table shows the matched peptides (green color) in T4(*Dys*)-AVV group based on reference human DMD sp|P11532|.

**Cryo-EM reconstruction**	
**Microscope**	Titan Krios
**Magnification**	64,000
**Voltage (kV)**	300
**Detector**	Gatan K3 Summit
**Recording mode**	super-resolution
**Total dose (e^-/Å^2)**	36
**Pixel size (Å)**	0.666 (1.85 after rescaling)
**Number of particles**	70340
**Symmetry imposed**	D5
**Map resolution (FSC 0.143; Å)**	3.9
**Map sharpening B-factor (Å^2)**	-129
**Structure refinement**	
**R.m.s. deviations**	
**Bond lengths (Å)**	0.003
**Bond angles (°)**	0.51
**Dihedral angles (°)**	12.7
**Ramachandran plot**	
**Favored (%)**	88.2
**Allowed (%)**	11.3
**Disallowed (%)**	0.5
**Clashscore**	10.8
**Rotamers outliers (%)**	7
**CC_{mask}**	0.85

Supplemental Table 2: Cryo-EM data and refinement statistics of 9DE-T4 capsid.

Supplemental Note 1: Sequences of DNA template, gRNA, and recombinant protein used in this study.

1. DNA template for AAVS1gRNA *in vitro* synthesis.

AAVS1 target sequence

AagcTAATACGACTCACTATAGGGGCCACTAGGGACAGGATGTTTTAGAGCTAGAAATAGCAAGTTAA

T7 promoter gRNA scafold sequence

AATAAGGCTAGTCCGTTATCAACTTGAAAAAGTGGCACCGAGTCGGTGCTTTT

gRNA scafold sequence

2. gRNA sequence.

AAVS1-Cas9-gRNA1: GGGGCCACTAGGGACAGGAT

AAVS1-Cas9-gRNA2: CTCCCTCCCAGGATCCTCTC

HBB-Cas9-gRNA: AGTCTGCCGTTACTGCCCTG

ipIII-Cas9-gRNA: GGCCTTTACTACAGAAGCTT

gp23-Cas9-gRNA: GTTCAAGTAACAATCGATGC

ipI-Cpf1- gRNA1: TTCAGCAGGAGAGATAACGATTG

ipI-Cpf1-gRNA2: TACCATTACCGAAGCTACTCTTA

ipII-Cpf1-gRNA1: CTTCTAAGTTCGGCATGTCTATG

ipII-Cpf1-gRNA2: TTACGGTCTTTATCGGGCAA
ipIII-Cpf1-gRNA1: AAGTCGGAAGCCTTTGTAGCTAA

ipIII-Cpf1-gRNA2: TGCTTGGCAAATTCAAGACCTGC

3. Primers for donor DNA insertion identification.

P1: CTGCCGTCTCTCTCCTGAGT

P2:GTGGGCTTGTACTCGGTCAT

P3:AAAACTGACGCACGGAGGAA

P4:GTGGATTCGGGTCACCTCTC

4. Some protein sequences.

a. Cas9-Soc:

PKKK: SV40 NLS; MDKKY: SpCas9; GGYVNI: Soc

PKKKRKVMDKKYSIGLDIGTNSVGWAVITDEYKVPSKKFKVLGNTDRHSIKKNLIGALLFD
SGETAEATRLKRTARRRYTRRKNRICYLQEIFSNEMAKVDDSFFHRLEESFLVEEDKKHE
RHPIFGNIVDEVAYHEKYPTIHLRKKLVDSTDKADLRLIYLALAHMIKFRGHFLIEGDLNPD
NSDVDKLFIQLVQTYNQLFEENPINASGVDAKAILSARLSKSRRLENLIAQLPGEKKNGLF
GNLIALSLGLTPNFKSNFDLAEDAKLQLSKDTYDDDLDNLLAQIGDQYADLFLAAKNLSDA
ILLSDILRVNTEITKAPLSASMIKRYDEHHQDLTLLKALVRQQLPEKYKEIFFDQSKNGYAG
YIDGGASQEEFYKFIKPILEKMDGTEELLVKLNREDLLRKQRTFDNGSIPHQIHLGELHAIL
RRQEDFYPFLKDNREKIEKILTFRIPYYVGPLARGNSRFAWMTRKSEETITPWNFEEVVD
KGASAQSFIERMTNFDKNLPNEKVLPKHSLLYEYFTVYNELTKVKYVTEGMRKPAFLSGE
QKKAIVDLLFKTNRKVTVKQLKEDYFKKIECFDSVEISGVEDRFNASLGTYHDLLKIIKDKD
FLDNEENEDILEDIVLTLTLFEDREMIEERLKTYAHLFDDKVMKQLKRRRYTGWGRLSRK
LINGIRDKQSGKTILDFLKSDGFANRNFMQLIHDDSLTFKEDIQKAQVSGQGDSLHEHIAN
LAGSPAIKKGILQTVKVVDELVKVMGRHKPENIVIEMARENQTTQKGQKNSRERMKRIEE
GIKELGSQILKEHPVENTQLQNEKLYLYYLQNGRDMYVDQELDINRLSDYDVDHIVPQSF
LKDDSIDNKVLTRSDKNRGKSDNVPSEEVVKKMKNYWRQLLNAKLITQRKFDNLTKAER
GGLSELDKAGFIKRQLVETRQITKHVAQILDSRMNTKYDENDKLIREVKVITLKSKLVSDF
RKDFQFYKVREINNYHHAHDAYLNAVVGTALIKKYPKLESEFVYGDYKVYDVRKMIAKSE
QEIGKATAKYFFYSNIMNFFKTEITLANGEIRKRPLIETNGETGEIVWDKGRDFATVRKVLS
MPQVNIVKKTEVQTGGFSKESILPKRNSDKLIARKKDWDPKKYGGFDSPTVAYSVLVVAK
VEKGKSKKLKSVKELLGITIMERSSFEKNPIDFLEAKGYKEVKKDLIIKLPKYSLFELENGR
KRMLASAGELQKGNELALPSKYVNFLYLASHYEKLKGSPEDNEQKQLFVEQHKHYLDEII
EQISEFSKRVILADANLDKVLSAYNKHRDKPIREQAENIIHLFTLTNLGAPAAFKYFDTTIDR
KRYTSTKEVLDATLIHQSITGLYERIDLSQLGGDGGGGSRSGGYVNIKTFTHPAGEGKEV
KGMEVSVPFEIYSNEHRIADAHYQTFPSEKAAYTVVTDAADWRTKNAAMFTPTPVSGHH
HHHH

b. Cpf1-Soc:

PKKK: SV40 NLS; MSKLE: LbCpf1; GGYVNI: Soc

PKKKRKVMSKLEKFTNCYSLSKTLRFKAIPVGKTQENIDNKRLLVEDEKRAEDYKGVKKL
LDRYYLSFINDVLHSIKLKNLNNYISLFRKKTRTEKENKELENLEINLRKEIAKAFKGNEGY
KSLFKKDIIETILPEFLDDKDEIALVNSFNGFTTAFTGFFDNRENMFSEEAKSTSIAFRCINE
NLTRYISNMDIFEKVDAIFDKHEVQEIKEKILNSDYDVEDFFEGEFFNFVLTQEGIDVYNAII
GGFVTESGEKIKGLNEYINLYNQKTKQKLPKFKPLYKQVLSDRESLSFYGEGYTSDEEVE

VFRNTLNKNSEIFSSIKKLEKLFKNFDEYSSAGIFVKNGPAISTISKDIFGEWNVIRDKWNA
EYDDIHLKKKAVVTEKYEDDRRKSFKKIGSFSLEQLQEYADADLSVVEKLKEIIIQKVDEIY
KVYGSSEKLFDADFVLEKSLKKNDAVVAIMKDLLDSVKSFENYIKAFFGEGKETNRDESF
YGDFVLAYDILLKVDHIYDAIRNYVTQKPYSKDKFKLYFQNPQFMGGWDKDKETDYRATI
LRYGSKYYLAIMDKKYAKCLQKIDKDDVNGNYEKINYKLLPGPNKMLPKVFFSKKWMAY
YNPSEDIQKIYKNGTFKKGDMFNLNDCHKLIDFFKDSISRYPKWSNAYDFNFSETEKYKD
IAGFYREVEEQGYKVSFESASKKEVDKLVEEGKLYMFQIYNKDFSDKSHGTPNLHTMYF
KLLFDENNHGQIRLSGGAELFMRRASLKKEELVVHPANSPIANKNPDNPKKTTTLSYDVY
KDKRFSEDQYELHIPIAINKCPKNIFKINTEVRVLLKHDDNPYVIGIDRGERNLLYIVVVDGK
GNIVEQYSLNEIINNFNGIRIKTDYHSLLDKKEKERFEARQNWTSIENIKELKAGYISQVVH
KICELVEKYDAVIALEDLNSGFKNSRVKVEKQVYQKFEKMLIDKLNYMVDKKSNPCATGG
ALKGYQITNKFESFKSMSTQNGFIFYIPAWLTSKIDPSTGFVNLLKTKYTSIADSKKFISSF
DRIMYVPEEDLFEFALDYKNFSRTDADYIKKWKLYSYGNRIRIFRNPKKNNVFDWEEVCL
TSAYKELFNKYGINYQQGDIRALLCEQSDKAFYSSFMALMSLMLQMRNSITGRTDVDFLI
SPVKNSDGIFYDSRNYEAQENAILPKNADANGAYNIARKVLWAIGQFKKAEDEKLDKVKI
AISNKEWLEYAQTSVKHAAAGGGGSGGGGSRSGGYVNIKTFTHPAGEGKEVKGMEVS
VPFEIYSNEHRIADAHYQTFPSEKAAYTTVVTDAADWRTKNAAMFTPTPVSGLEHHHHH
H

c. Cre-Hoc:

MSNLLT: Cre; FTVDIT: Hoc

MSNLLTVHQNLPALPVDATSDEVRKNLMDMFRDRQAFSEHTWKMLLSVCRSWAAWCK
LNNRKWFPAEPEDVRDYLLYLQARGLAVKTIQQHLGQLNMLHRRSGLPRPSDSNAVSLV
MRRIRKENVDAGERAKQALAFERTDFDQVRSLMENSDRCQDIRNLAFLGIAYNTLLRIAE
IARIRVKDISRTDGGRMLIHIGRTKTLVSTAGVEKALSLGVTKLVERWISVSGVADDPNNYL
FCRVRKNGVAAPSATSQLSTRALEGIFEATHRLIYGAKDDSGQRYLAWSGHSARVGAAR
DMARAGVSIPEIMQAGGWTNVNIVMNYIRNLDSETGAMVRLLEDGDGGSEFGGSGGS
GGSGGSFTVDITPKTPTGVIDETKQFTATPSGQTGGGTITYAWSVDNVPQDGAEATFSY
VLKGPAGQKTIKVVATNTLSEGGPETAEATTTITVKNKTQTTTLAVTPASPAAGVIGTPVQF
TAALASQPDGASATYQWYVDDSQVGGETNSTFSYTPTTSGVKRIKCVAQVTATDYDALS
VTSNEVSLTVNKKTMNPQVTLTPPSINVQQDASATFTANVTGAPEEAQITYSWKKDSSPV
EGSTNVYTVDTSSVGSQTIEVTATVTAADYNPVTVTKTGNVTVTAKVAPEPEGELPYVHP
LPHRSSAYIWCGWWVMDEIQKMTEEGKDWKTDDPDSKYYLHRYTLQKMMKDYPEVDV
QESRNGYIIHKTALETGIIYTYPGSHHHHHH

d. RGD-Hoc:

CDCRGD: RGD; FTVDIT: Hoc

CDCRGDCFCSAMTFTVDITPKTPTGVIDETKQFTATPSGQTGGGTITYAWSVDNVPQDG
AEATFSYVLKGPAGQKTIKVVATNTLSEGGPETAEATTTITVKNKTQTTTLAVTPASPAAG
VIGTPVQFTAALASQPDGASATYQWYVDDSQVGGETNSTFSYTPTTSGVKRIKCVAQVT

ATDYDALSVTSNEVSLTVNKKTMNPQVTLTPPSINVQQDASATFTANVTGAPEEAQITYS
WKKDSSPVEGSTNVYTVDTSSVGSQTIEVTATVTAADYNPVTVTKTGNVTVTAKVAPEP
EGELPYVHPLPHRSSAYIWCGWWVMDEIQKMTEEGKDWKTDDPDSKYYLHRYTLQKM
MKDYPEVDVQESRNGYIIHKTALETGIIYTYPKLAAALEHHHHHH

e. CTS-LacI-NLS:

MKTYQ: CTS; MKYVTLYD: LacI N; KRPAATKK: NLS

MKTYQEFIAEMKYVTLYDVAEYAGVSHQTVSRVVNQASHVSAKTREKVEAAMAELNYIP
NRVAQQLAGKQSLLIKRPAATKKAGQAKKKK

Supplemental Note 2: Full wwPDB EM validation report.

Full wwPDB EM Validation Report (i)

Apr 12, 2023 – 07:31 PM EDT

PDB ID	:	8GMO
EMDB ID	:	EMD-40228
Title	:	Bacteriophage T4 capsid shell containing 9DE insertions into the gp23* major capsid protein subunits
Deposited on	:	2023-03-26
Resolution	:	3.90 Å(reported)
Based on initial model	:	7VS5

This wwPDB validation report is for manuscript review

This is a Full wwPDB EM Validation Report.

This report is produced by the wwPDB biocuration pipeline after annotation of the structure.

We welcome your comments at *validation@mail.wwpdb.org*
A user guide is available at
https://www.wwpdb.org/validation/2017/EMValidationReportHelp
with specific help available everywhere you see the (i) symbol.

The types of validation reports are described at
http://www.wwpdb.org/validation/2017/FAQs#types.

The following versions of software and data (see references (i)) were used in the production of this report:

EMDB validation analysis	:	0.0.1.dev50
MolProbity	:	4.02b-467
Percentile statistics	:	20191225.v01 (using entries in the PDB archive December 25th 2019)
MapQ	:	1.9.9
Ideal geometry (proteins)	:	Engh & Huber (2001)
Ideal geometry (DNA, RNA)	:	Parkinson et al. (1996)
Validation Pipeline (wwPDB-VP)	:	2.32.2

For Manuscript Review

1 Overall quality at a glance (i)

The following experimental techniques were used to determine the structure:
ELECTRON MICROSCOPY

The reported resolution of this entry is 3.90 Å.

Percentile scores (ranging between 0-100) for global validation metrics of the entry are shown in the following graphic. The table shows the number of entries on which the scores are based.

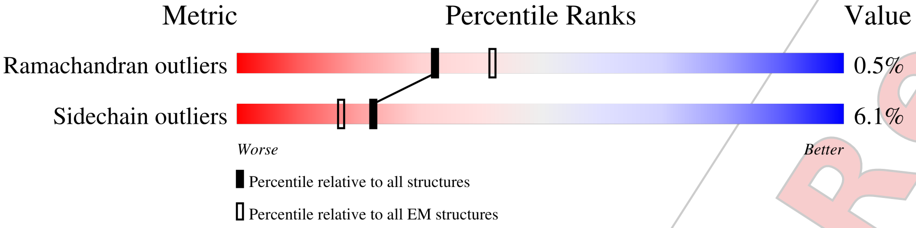

Metric	Whole archive (#Entries)	EM structures (#Entries)
Ramachandran outliers	154571	4023
Sidechain outliers	154315	3826

The table below summarises the geometric issues observed across the polymeric chains and their fit to the map. The red, orange, yellow and green segments of the bar indicate the fraction of residues that contain outliers for >=3, 2, 1 and 0 types of geometric quality criteria respectively. A grey segment represents the fraction of residues that are not modelled. The numeric value for each fraction is indicated below the corresponding segment, with a dot representing fractions <=5% The upper red bar (where present) indicates the fraction of residues that have poor fit to the EM map (all-atom inclusion < 40%). The numeric value is given above the bar.

Mol	Chain	Length	Quality of chain
1	0	465	• 94% 6%
1	1	465	• 96% •
1	2	465	• 94% 6%
1	3	465	• 96% •
1	4	465	• 94% 6%
1	5	465	• 95% 5%
1	6	465	• 97% •
1	7	465	• 97% •

Continued on next page...

For Manuscript Review

Continued from previous page...

Mol	Chain	Length	Quality of chain
1	8	465	92% 8%
1	9	465	96% •
1	AA	465	96% •
1	AB	465	• 94% 6%
1	AC	465	• 94% 6%
1	AD	465	90% 5% 6%
1	AE	465	• 95% 5%
1	AF	465	92% 8%
1	AG	465	96% •
1	AH	465	92% 8%
1	AI	465	93% 7%
1	AJ	465	• 94% 6%
1	AK	465	95% 5%
1	AL	465	94% 6%
1	AM	465	95% 5%
1	AN	465	• 97% •
1	AO	465	97% •
1	AP	465	• 96% •
1	AQ	465	94% 6%
1	AR	465	95% 5%
1	AS	465	• 96% •
1	AT	465	92% 8%
1	AU	465	95% 5%
1	AV	465	• 93% 7%
1	AW	465	96% •

Continued on next page...

For Manuscript Review

Continued from previous page...

Mol	Chain	Length	Quality of chain
1	AX	465	94% 6%
1	AY	465	95% 5%
1	AZ	465	• 97% •
1	Aa	465	97% •
1	Ab	465	95% 5%
1	Ac	465	96% •
1	Ad	465	96% •
1	Ae	465	96% •
1	Af	465	91% 9%
1	Ag	465	95% 5%
1	Ah	465	• 94% 6%
1	Ai	465	• 95% 5%
1	Aj	465	95% 5%
1	Ak	465	96% •
1	Al	465	97% •
1	Am	465	• 98% •
1	G	465	• 94% 6%
1	H	465	95% 5%
1	J	465	95% •
1	K	465	95% 5%
1	L	465	97% •
1	M	465	96% •
1	N	465	97% •
1	O	465	91% 9%
1	P	465	94% 6%

Continued on next page...

For Manuscript Review

Continued from previous page...

Mol	Chain	Length	Quality of chain
1	Q	465	94% 6%
1	R	465	95% 5%
1	S	465	94% 6%
1	T	465	95% 5%
1	U	465	95% 5%
1	V	465	96% •
1	W	465	96% •
1	X	465	97% •
1	Y	465	93% 7%
1	Z	465	94% 6%
1	b	465	94% 6%
1	c	465	97% •
1	d	465	96% •
1	e	465	96% •
1	f	465	92% 8%
1	g	465	97% •
1	h	465	97% •
1	i	465	97% •
1	j	465	98% •
1	k	465	95% 5%
1	l	465	95% 5%
1	m	465	95% 5%
1	n	465	96% •
1	o	465	95% 5%
1	p	465	94% 6%

Continued on next page...

For Manuscript Review

Continued from previous page...

Mol	Chain	Length	Quality of chain
1	q	465	• 95% 5%
1	r	465	• 94% 6%
1	s	465	96% •
1	t	465	• 94% 6%
1	u	465	96% •
1	v	465	• 97% •
1	w	465	• 97% •
1	x	465	• 96% •
1	y	465	• 95% 5%
1	z	465	• 91% 9%
2	A	415	91% 8%
2	B	415	• 90% 10%
2	C	415	• 89% 11%
2	D	415	• 90% 10%
2	a	415	90% 10%

For Manuscript Review

2 Entry composition (i)

There are 2 unique types of molecules in this entry. The entry contains 341379 atoms, of which 0 are hydrogens and 0 are deuteriums.

In the tables below, the AltConf column contains the number of residues with at least one atom in alternate conformation and the Trace column contains the number of residues modelled with at most 2 atoms.

- Molecule 1 is a protein called Mature major capsid protein.

Mol	Chain	Residues	Atoms					AltConf	Trace
1	0	465	Total 3501	C 2207	N 593	O 686	S 15	0	0
1	1	465	Total 3501	C 2207	N 593	O 686	S 15	0	0
1	2	465	Total 3501	C 2207	N 593	O 686	S 15	0	0
1	3	465	Total 3501	C 2207	N 593	O 686	S 15	0	0
1	4	465	Total 3501	C 2207	N 593	O 686	S 15	0	0
1	5	465	Total 3501	C 2207	N 593	O 686	S 15	0	0
1	6	465	Total 3501	C 2207	N 593	O 686	S 15	0	0
1	7	465	Total 3501	C 2207	N 593	O 686	S 15	0	0
1	8	465	Total 3501	C 2207	N 593	O 686	S 15	0	0
1	9	465	Total 3501	C 2207	N 593	O 686	S 15	0	0
1	AA	465	Total 3501	C 2207	N 593	O 686	S 15	0	0
1	AB	465	Total 3501	C 2207	N 593	O 686	S 15	0	0
1	AC	465	Total 3501	C 2207	N 593	O 686	S 15	0	0
1	AD	439	Total 3312	C 2092	N 559	O 646	S 15	0	0
1	AE	465	Total 3501	C 2207	N 593	O 686	S 15	0	0
1	AF	465	Total 3501	C 2207	N 593	O 686	S 15	0	0
1	AG	465	Total 3501	C 2207	N 593	O 686	S 15	0	0

Continued on next page...

For Manuscript Review

Continued from previous page...

Mol	Chain	Residues	Atoms					AltConf	Trace
1	AH	465	Total 3501	C 2207	N 593	O 686	S 15	0	0
1	AI	465	Total 3501	C 2207	N 593	O 686	S 15	0	0
1	AJ	465	Total 3501	C 2207	N 593	O 686	S 15	0	0
1	AK	465	Total 3501	C 2207	N 593	O 686	S 15	0	0
1	AL	465	Total 3501	C 2207	N 593	O 686	S 15	0	0
1	AM	465	Total 3501	C 2207	N 593	O 686	S 15	0	0
1	AN	465	Total 3501	C 2207	N 593	O 686	S 15	0	0
1	AO	465	Total 3501	C 2207	N 593	O 686	S 15	0	0
1	AP	465	Total 3488	C 2197	N 591	O 685	S 15	0	0
1	AQ	465	Total 3501	C 2207	N 593	O 686	S 15	0	0
1	AR	465	Total 3501	C 2207	N 593	O 686	S 15	0	0
1	AS	465	Total 3501	C 2207	N 593	O 686	S 15	0	0
1	AT	465	Total 3501	C 2207	N 593	O 686	S 15	0	0
1	AU	465	Total 3501	C 2207	N 593	O 686	S 15	0	0
1	AV	465	Total 3501	C 2207	N 593	O 686	S 15	0	0
1	AW	465	Total 3501	C 2207	N 593	O 686	S 15	0	0
1	AX	465	Total 3501	C 2207	N 593	O 686	S 15	0	0
1	AY	465	Total 3501	C 2207	N 593	O 686	S 15	0	0
1	AZ	465	Total 3501	C 2207	N 593	O 686	S 15	0	0
1	Aa	465	Total 3501	C 2207	N 593	O 686	S 15	0	0
1	Ab	465	Total 3488	C 2197	N 591	O 685	S 15	0	0

Continued on next page...

For Manuscript Review

Continued from previous page...

Mol	Chain	Residues	Atoms					AltConf	Trace
1	Ac	465	Total 3501	C 2207	N 593	O 686	S 15	0	0
1	Ad	465	Total 3501	C 2207	N 593	O 686	S 15	0	0
1	Ae	465	Total 3501	C 2207	N 593	O 686	S 15	0	0
1	Af	465	Total 3501	C 2207	N 593	O 686	S 15	0	0
1	Ag	465	Total 3501	C 2207	N 593	O 686	S 15	0	0
1	Ah	465	Total 3501	C 2207	N 593	O 686	S 15	0	0
1	Ai	465	Total 3501	C 2207	N 593	O 686	S 15	0	0
1	Aj	465	Total 3501	C 2207	N 593	O 686	S 15	0	0
1	Ak	465	Total 3501	C 2207	N 593	O 686	S 15	0	0
1	Al	465	Total 3501	C 2207	N 593	O 686	S 15	0	0
1	Am	465	Total 3501	C 2207	N 593	O 686	S 15	0	0
1	G	465	Total 3501	C 2207	N 593	O 686	S 15	0	0
1	H	465	Total 3501	C 2207	N 593	O 686	S 15	0	0
1	J	465	Total 3501	C 2207	N 593	O 686	S 15	0	0
1	K	465	Total 3488	C 2197	N 591	O 685	S 15	0	0
1	L	465	Total 3501	C 2207	N 593	O 686	S 15	0	0
1	M	465	Total 3501	C 2207	N 593	O 686	S 15	0	0
1	N	465	Total 3501	C 2207	N 593	O 686	S 15	0	0
1	O	465	Total 3501	C 2207	N 593	O 686	S 15	0	0
1	P	465	Total 3501	C 2207	N 593	O 686	S 15	0	0
1	Q	465	Total 3501	C 2207	N 593	O 686	S 15	0	0

Continued on next page...

For Manuscript Review

Continued from previous page...

Mol	Chain	Residues	Atoms					AltConf	Trace
1	R	465	Total 3501	C 2207	N 593	O 686	S 15	0	0
1	S	465	Total 3501	C 2207	N 593	O 686	S 15	0	0
1	T	465	Total 3501	C 2207	N 593	O 686	S 15	0	0
1	U	465	Total 3488	C 2197	N 591	O 685	S 15	0	0
1	V	465	Total 3501	C 2207	N 593	O 686	S 15	0	0
1	W	465	Total 3501	C 2207	N 593	O 686	S 15	0	0
1	X	465	Total 3501	C 2207	N 593	O 686	S 15	0	0
1	Y	465	Total 3501	C 2207	N 593	O 686	S 15	0	0
1	Z	465	Total 3501	C 2207	N 593	O 686	S 15	0	0
1	b	465	Total 3501	C 2207	N 593	O 686	S 15	0	0
1	c	465	Total 3501	C 2207	N 593	O 686	S 15	0	0
1	d	465	Total 3501	C 2207	N 593	O 686	S 15	0	0
1	e	465	Total 3501	C 2207	N 593	O 686	S 15	0	0
1	f	465	Total 3501	C 2207	N 593	O 686	S 15	0	0
1	g	465	Total 3501	C 2207	N 593	O 686	S 15	0	0
1	h	465	Total 3501	C 2207	N 593	O 686	S 15	0	0
1	i	465	Total 3501	C 2207	N 593	O 686	S 15	0	0
1	j	465	Total 3501	C 2207	N 593	O 686	S 15	0	0
1	k	465	Total 3501	C 2207	N 593	O 686	S 15	0	0
1	l	465	Total 3501	C 2207	N 593	O 686	S 15	0	0
1	m	465	Total 3501	C 2207	N 593	O 686	S 15	0	0

Continued on next page...

For Manuscript Review

Continued from previous page...

Mol	Chain	Residues	Atoms					AltConf	Trace
			Total	C	N	O	S		
1	n	465	3501	2207	593	686	15	0	0
1	o	465	3501	2207	593	686	15	0	0
1	p	465	3501	2207	593	686	15	0	0
1	q	465	3501	2207	593	686	15	0	0
1	r	465	3501	2207	593	686	15	0	0
1	s	465	3501	2207	593	686	15	0	0
1	t	465	3501	2207	593	686	15	0	0
1	u	465	3501	2207	593	686	15	0	0
1	v	465	3488	2197	591	685	15	0	0
1	w	465	3501	2207	593	686	15	0	0
1	x	465	3501	2207	593	686	15	0	0
1	y	465	3501	2207	593	686	15	0	0
1	z	465	3501	2207	593	686	15	0	0

There are 837 discrepancies between the modelled and reference sequences:

Chain	Residue	Modelled	Actual	Comment	Reference
0	201A	ASP	-	insertion	UNP P04535
0	201B	GLU	-	insertion	UNP P04535
0	201C	ASP	-	insertion	UNP P04535
0	201D	GLU	-	insertion	UNP P04535
0	201E	ASP	-	insertion	UNP P04535
0	201F	GLU	-	insertion	UNP P04535
0	201G	ASP	-	insertion	UNP P04535
0	201H	GLU	-	insertion	UNP P04535
0	201I	ASP	-	insertion	UNP P04535
1	201A	ASP	-	insertion	UNP P04535
1	201B	GLU	-	insertion	UNP P04535
1	201C	ASP	-	insertion	UNP P04535
1	201D	GLU	-	insertion	UNP P04535

Continued on next page...

For Manuscript Review

Continued from previous page...

Chain	Residue	Modelled	Actual	Comment	Reference
1	201E	ASP	-	insertion	UNP P04535
1	201F	GLU	-	insertion	UNP P04535
1	201G	ASP	-	insertion	UNP P04535
1	201H	GLU	-	insertion	UNP P04535
1	201I	ASP	-	insertion	UNP P04535
2	201A	ASP	-	insertion	UNP P04535
2	201B	GLU	-	insertion	UNP P04535
2	201C	ASP	-	insertion	UNP P04535
2	201D	GLU	-	insertion	UNP P04535
2	201E	ASP	-	insertion	UNP P04535
2	201F	GLU	-	insertion	UNP P04535
2	201G	ASP	-	insertion	UNP P04535
2	201H	GLU	-	insertion	UNP P04535
2	201I	ASP	-	insertion	UNP P04535
3	201A	ASP	-	insertion	UNP P04535
3	201B	GLU	-	insertion	UNP P04535
3	201C	ASP	-	insertion	UNP P04535
3	201D	GLU	-	insertion	UNP P04535
3	201E	ASP	-	insertion	UNP P04535
3	201F	GLU	-	insertion	UNP P04535
3	201G	ASP	-	insertion	UNP P04535
3	201H	GLU	-	insertion	UNP P04535
3	201I	ASP	-	insertion	UNP P04535
4	201A	ASP	-	insertion	UNP P04535
4	201B	GLU	-	insertion	UNP P04535
4	201C	ASP	-	insertion	UNP P04535
4	201D	GLU	-	insertion	UNP P04535
4	201E	ASP	-	insertion	UNP P04535
4	201F	GLU	-	insertion	UNP P04535
4	201G	ASP	-	insertion	UNP P04535
4	201H	GLU	-	insertion	UNP P04535
4	201I	ASP	-	insertion	UNP P04535
5	201A	ASP	-	insertion	UNP P04535
5	201B	GLU	-	insertion	UNP P04535
5	201C	ASP	-	insertion	UNP P04535
5	201D	GLU	-	insertion	UNP P04535
5	201E	ASP	-	insertion	UNP P04535
5	201F	GLU	-	insertion	UNP P04535
5	201G	ASP	-	insertion	UNP P04535
5	201H	GLU	-	insertion	UNP P04535
5	201I	ASP	-	insertion	UNP P04535
6	201A	ASP	-	insertion	UNP P04535

Continued on next page...

For Manuscript Review

Continued from previous page...

Chain	Residue	Modelled	Actual	Comment	Reference
6	201B	GLU	-	insertion	UNP P04535
6	201C	ASP	-	insertion	UNP P04535
6	201D	GLU	-	insertion	UNP P04535
6	201E	ASP	-	insertion	UNP P04535
6	201F	GLU	-	insertion	UNP P04535
6	201G	ASP	-	insertion	UNP P04535
6	201H	GLU	-	insertion	UNP P04535
6	201I	ASP	-	insertion	UNP P04535
7	201A	ASP	-	insertion	UNP P04535
7	201B	GLU	-	insertion	UNP P04535
7	201C	ASP	-	insertion	UNP P04535
7	201D	GLU	-	insertion	UNP P04535
7	201E	ASP	-	insertion	UNP P04535
7	201F	GLU	-	insertion	UNP P04535
7	201G	ASP	-	insertion	UNP P04535
7	201H	GLU	-	insertion	UNP P04535
7	201I	ASP	-	insertion	UNP P04535
8	201A	ASP	-	insertion	UNP P04535
8	201B	GLU	-	insertion	UNP P04535
8	201C	ASP	-	insertion	UNP P04535
8	201D	GLU	-	insertion	UNP P04535
8	201E	ASP	-	insertion	UNP P04535
8	201F	GLU	-	insertion	UNP P04535
8	201G	ASP	-	insertion	UNP P04535
8	201H	GLU	-	insertion	UNP P04535
8	201I	ASP	-	insertion	UNP P04535
9	201A	ASP	-	insertion	UNP P04535
9	201B	GLU	-	insertion	UNP P04535
9	201C	ASP	-	insertion	UNP P04535
9	201D	GLU	-	insertion	UNP P04535
9	201E	ASP	-	insertion	UNP P04535
9	201F	GLU	-	insertion	UNP P04535
9	201G	ASP	-	insertion	UNP P04535
9	201H	GLU	-	insertion	UNP P04535
9	201I	ASP	-	insertion	UNP P04535
AA	201A	ASP	-	insertion	UNP P04535
AA	201B	GLU	-	insertion	UNP P04535
AA	201C	ASP	-	insertion	UNP P04535
AA	201D	GLU	-	insertion	UNP P04535
AA	201E	ASP	-	insertion	UNP P04535
AA	201F	GLU	-	insertion	UNP P04535
AA	201G	ASP	-	insertion	UNP P04535

Continued on next page...

For Manuscript Review

Continued from previous page...

Chain	Residue	Modelled	Actual	Comment	Reference
AA	201H	GLU	-	insertion	UNP P04535
AA	201I	ASP	-	insertion	UNP P04535
AB	201A	ASP	-	insertion	UNP P04535
AB	201B	GLU	-	insertion	UNP P04535
AB	201C	ASP	-	insertion	UNP P04535
AB	201D	GLU	-	insertion	UNP P04535
AB	201E	ASP	-	insertion	UNP P04535
AB	201F	GLU	-	insertion	UNP P04535
AB	201G	ASP	-	insertion	UNP P04535
AB	201H	GLU	-	insertion	UNP P04535
AB	201I	ASP	-	insertion	UNP P04535
AC	201A	ASP	-	insertion	UNP P04535
AC	201B	GLU	-	insertion	UNP P04535
AC	201C	ASP	-	insertion	UNP P04535
AC	201D	GLU	-	insertion	UNP P04535
AC	201E	ASP	-	insertion	UNP P04535
AC	201F	GLU	-	insertion	UNP P04535
AC	201G	ASP	-	insertion	UNP P04535
AC	201H	GLU	-	insertion	UNP P04535
AC	201I	ASP	-	insertion	UNP P04535
AD	201A	ASP	-	insertion	UNP P04535
AD	201B	GLU	-	insertion	UNP P04535
AD	201C	ASP	-	insertion	UNP P04535
AD	201D	GLU	-	insertion	UNP P04535
AD	201E	ASP	-	insertion	UNP P04535
AD	201F	GLU	-	insertion	UNP P04535
AD	201G	ASP	-	insertion	UNP P04535
AD	201H	GLU	-	insertion	UNP P04535
AD	201I	ASP	-	insertion	UNP P04535
AE	201A	ASP	-	insertion	UNP P04535
AE	201B	GLU	-	insertion	UNP P04535
AE	201C	ASP	-	insertion	UNP P04535
AE	201D	GLU	-	insertion	UNP P04535
AE	201E	ASP	-	insertion	UNP P04535
AE	201F	GLU	-	insertion	UNP P04535
AE	201G	ASP	-	insertion	UNP P04535
AE	201H	GLU	-	insertion	UNP P04535
AE	201I	ASP	-	insertion	UNP P04535
AF	201A	ASP	-	insertion	UNP P04535
AF	201B	GLU	-	insertion	UNP P04535
AF	201C	ASP	-	insertion	UNP P04535
AF	201D	GLU	-	insertion	UNP P04535

Continued on next page...

For Manuscript Review

Continued from previous page...

Chain	Residue	Modelled	Actual	Comment	Reference
AF	201E	ASP	-	insertion	UNP P04535
AF	201F	GLU	-	insertion	UNP P04535
AF	201G	ASP	-	insertion	UNP P04535
AF	201H	GLU	-	insertion	UNP P04535
AF	201I	ASP	-	insertion	UNP P04535
AG	201A	ASP	-	insertion	UNP P04535
AG	201B	GLU	-	insertion	UNP P04535
AG	201C	ASP	-	insertion	UNP P04535
AG	201D	GLU	-	insertion	UNP P04535
AG	201E	ASP	-	insertion	UNP P04535
AG	201F	GLU	-	insertion	UNP P04535
AG	201G	ASP	-	insertion	UNP P04535
AG	201H	GLU	-	insertion	UNP P04535
AG	201I	ASP	-	insertion	UNP P04535
AH	201A	ASP	-	insertion	UNP P04535
AH	201B	GLU	-	insertion	UNP P04535
AH	201C	ASP	-	insertion	UNP P04535
AH	201D	GLU	-	insertion	UNP P04535
AH	201E	ASP	-	insertion	UNP P04535
AH	201F	GLU	-	insertion	UNP P04535
AH	201G	ASP	-	insertion	UNP P04535
AH	201H	GLU	-	insertion	UNP P04535
AH	201I	ASP	-	insertion	UNP P04535
AI	201A	ASP	-	insertion	UNP P04535
AI	201B	GLU	-	insertion	UNP P04535
AI	201C	ASP	-	insertion	UNP P04535
AI	201D	GLU	-	insertion	UNP P04535
AI	201E	ASP	-	insertion	UNP P04535
AI	201F	GLU	-	insertion	UNP P04535
AI	201G	ASP	-	insertion	UNP P04535
AI	201H	GLU	-	insertion	UNP P04535
AI	201I	ASP	-	insertion	UNP P04535
AJ	201A	ASP	-	insertion	UNP P04535
AJ	201B	GLU	-	insertion	UNP P04535
AJ	201C	ASP	-	insertion	UNP P04535
AJ	201D	GLU	-	insertion	UNP P04535
AJ	201E	ASP	-	insertion	UNP P04535
AJ	201F	GLU	-	insertion	UNP P04535
AJ	201G	ASP	-	insertion	UNP P04535
AJ	201H	GLU	-	insertion	UNP P04535
AJ	201I	ASP	-	insertion	UNP P04535
AK	201A	ASP	-	insertion	UNP P04535

Continued on next page...

For Manuscript Review

Continued from previous page...

Chain	Residue	Modelled	Actual	Comment	Reference
AK	201B	GLU	-	insertion	UNP P04535
AK	201C	ASP	-	insertion	UNP P04535
AK	201D	GLU	-	insertion	UNP P04535
AK	201E	ASP	-	insertion	UNP P04535
AK	201F	GLU	-	insertion	UNP P04535
AK	201G	ASP	-	insertion	UNP P04535
AK	201H	GLU	-	insertion	UNP P04535
AK	201I	ASP	-	insertion	UNP P04535
AL	201A	ASP	-	insertion	UNP P04535
AL	201B	GLU	-	insertion	UNP P04535
AL	201C	ASP	-	insertion	UNP P04535
AL	201D	GLU	-	insertion	UNP P04535
AL	201E	ASP	-	insertion	UNP P04535
AL	201F	GLU	-	insertion	UNP P04535
AL	201G	ASP	-	insertion	UNP P04535
AL	201H	GLU	-	insertion	UNP P04535
AL	201I	ASP	-	insertion	UNP P04535
AM	201A	ASP	-	insertion	UNP P04535
AM	201B	GLU	-	insertion	UNP P04535
AM	201C	ASP	-	insertion	UNP P04535
AM	201D	GLU	-	insertion	UNP P04535
AM	201E	ASP	-	insertion	UNP P04535
AM	201F	GLU	-	insertion	UNP P04535
AM	201G	ASP	-	insertion	UNP P04535
AM	201H	GLU	-	insertion	UNP P04535
AM	201I	ASP	-	insertion	UNP P04535
AN	201A	ASP	-	insertion	UNP P04535
AN	201B	GLU	-	insertion	UNP P04535
AN	201C	ASP	-	insertion	UNP P04535
AN	201D	GLU	-	insertion	UNP P04535
AN	201E	ASP	-	insertion	UNP P04535
AN	201F	GLU	-	insertion	UNP P04535
AN	201G	ASP	-	insertion	UNP P04535
AN	201H	GLU	-	insertion	UNP P04535
AN	201I	ASP	-	insertion	UNP P04535
AO	201A	ASP	-	insertion	UNP P04535
AO	201B	GLU	-	insertion	UNP P04535
AO	201C	ASP	-	insertion	UNP P04535
AO	201D	GLU	-	insertion	UNP P04535
AO	201E	ASP	-	insertion	UNP P04535
AO	201F	GLU	-	insertion	UNP P04535
AO	201G	ASP	-	insertion	UNP P04535

Continued on next page...

For Manuscript Review

Continued from previous page...

Chain	Residue	Modelled	Actual	Comment	Reference
AO	201H	GLU	-	insertion	UNP P04535
AO	201I	ASP	-	insertion	UNP P04535
AP	201A	ASP	-	insertion	UNP P04535
AP	201B	GLU	-	insertion	UNP P04535
AP	201C	ASP	-	insertion	UNP P04535
AP	201D	GLU	-	insertion	UNP P04535
AP	201E	ASP	-	insertion	UNP P04535
AP	201F	GLU	-	insertion	UNP P04535
AP	201G	ASP	-	insertion	UNP P04535
AP	201H	GLU	-	insertion	UNP P04535
AP	201I	ASP	-	insertion	UNP P04535
AQ	201A	ASP	-	insertion	UNP P04535
AQ	201B	GLU	-	insertion	UNP P04535
AQ	201C	ASP	-	insertion	UNP P04535
AQ	201D	GLU	-	insertion	UNP P04535
AQ	201E	ASP	-	insertion	UNP P04535
AQ	201F	GLU	-	insertion	UNP P04535
AQ	201G	ASP	-	insertion	UNP P04535
AQ	201H	GLU	-	insertion	UNP P04535
AQ	201I	ASP	-	insertion	UNP P04535
AR	201A	ASP	-	insertion	UNP P04535
AR	201B	GLU	-	insertion	UNP P04535
AR	201C	ASP	-	insertion	UNP P04535
AR	201D	GLU	-	insertion	UNP P04535
AR	201E	ASP	-	insertion	UNP P04535
AR	201F	GLU	-	insertion	UNP P04535
AR	201G	ASP	-	insertion	UNP P04535
AR	201H	GLU	-	insertion	UNP P04535
AR	201I	ASP	-	insertion	UNP P04535
AS	201A	ASP	-	insertion	UNP P04535
AS	201B	GLU	-	insertion	UNP P04535
AS	201C	ASP	-	insertion	UNP P04535
AS	201D	GLU	-	insertion	UNP P04535
AS	201E	ASP	-	insertion	UNP P04535
AS	201F	GLU	-	insertion	UNP P04535
AS	201G	ASP	-	insertion	UNP P04535
AS	201H	GLU	-	insertion	UNP P04535
AS	201I	ASP	-	insertion	UNP P04535
AT	201A	ASP	-	insertion	UNP P04535
AT	201B	GLU	-	insertion	UNP P04535
AT	201C	ASP	-	insertion	UNP P04535
AT	201D	GLU	-	insertion	UNP P04535

Continued on next page...

For Manuscript Review

Continued from previous page...

Chain	Residue	Modelled	Actual	Comment	Reference
AT	201E	ASP	-	insertion	UNP P04535
AT	201F	GLU	-	insertion	UNP P04535
AT	201G	ASP	-	insertion	UNP P04535
AT	201H	GLU	-	insertion	UNP P04535
AT	201I	ASP	-	insertion	UNP P04535
AU	201A	ASP	-	insertion	UNP P04535
AU	201B	GLU	-	insertion	UNP P04535
AU	201C	ASP	-	insertion	UNP P04535
AU	201D	GLU	-	insertion	UNP P04535
AU	201E	ASP	-	insertion	UNP P04535
AU	201F	GLU	-	insertion	UNP P04535
AU	201G	ASP	-	insertion	UNP P04535
AU	201H	GLU	-	insertion	UNP P04535
AU	201I	ASP	-	insertion	UNP P04535
AV	201A	ASP	-	insertion	UNP P04535
AV	201B	GLU	-	insertion	UNP P04535
AV	201C	ASP	-	insertion	UNP P04535
AV	201D	GLU	-	insertion	UNP P04535
AV	201E	ASP	-	insertion	UNP P04535
AV	201F	GLU	-	insertion	UNP P04535
AV	201G	ASP	-	insertion	UNP P04535
AV	201H	GLU	-	insertion	UNP P04535
AV	201I	ASP	-	insertion	UNP P04535
AW	201A	ASP	-	insertion	UNP P04535
AW	201B	GLU	-	insertion	UNP P04535
AW	201C	ASP	-	insertion	UNP P04535
AW	201D	GLU	-	insertion	UNP P04535
AW	201E	ASP	-	insertion	UNP P04535
AW	201F	GLU	-	insertion	UNP P04535
AW	201G	ASP	-	insertion	UNP P04535
AW	201H	GLU	-	insertion	UNP P04535
AW	201I	ASP	-	insertion	UNP P04535
AX	201A	ASP	-	insertion	UNP P04535
AX	201B	GLU	-	insertion	UNP P04535
AX	201C	ASP	-	insertion	UNP P04535
AX	201D	GLU	-	insertion	UNP P04535
AX	201E	ASP	-	insertion	UNP P04535
AX	201F	GLU	-	insertion	UNP P04535
AX	201G	ASP	-	insertion	UNP P04535
AX	201H	GLU	-	insertion	UNP P04535
AX	201I	ASP	-	insertion	UNP P04535
AY	201A	ASP	-	insertion	UNP P04535

Continued on next page...

For Manuscript Review

Continued from previous page...

Chain	Residue	Modelled	Actual	Comment	Reference
AY	201B	GLU	-	insertion	UNP P04535
AY	201C	ASP	-	insertion	UNP P04535
AY	201D	GLU	-	insertion	UNP P04535
AY	201E	ASP	-	insertion	UNP P04535
AY	201F	GLU	-	insertion	UNP P04535
AY	201G	ASP	-	insertion	UNP P04535
AY	201H	GLU	-	insertion	UNP P04535
AY	201I	ASP	-	insertion	UNP P04535
AZ	201A	ASP	-	insertion	UNP P04535
AZ	201B	GLU	-	insertion	UNP P04535
AZ	201C	ASP	-	insertion	UNP P04535
AZ	201D	GLU	-	insertion	UNP P04535
AZ	201E	ASP	-	insertion	UNP P04535
AZ	201F	GLU	-	insertion	UNP P04535
AZ	201G	ASP	-	insertion	UNP P04535
AZ	201H	GLU	-	insertion	UNP P04535
AZ	201I	ASP	-	insertion	UNP P04535
Aa	201A	ASP	-	insertion	UNP P04535
Aa	201B	GLU	-	insertion	UNP P04535
Aa	201C	ASP	-	insertion	UNP P04535
Aa	201D	GLU	-	insertion	UNP P04535
Aa	201E	ASP	-	insertion	UNP P04535
Aa	201F	GLU	-	insertion	UNP P04535
Aa	201G	ASP	-	insertion	UNP P04535
Aa	201H	GLU	-	insertion	UNP P04535
Aa	201I	ASP	-	insertion	UNP P04535
Ab	201A	ASP	-	insertion	UNP P04535
Ab	201B	GLU	-	insertion	UNP P04535
Ab	201C	ASP	-	insertion	UNP P04535
Ab	201D	GLU	-	insertion	UNP P04535
Ab	201E	ASP	-	insertion	UNP P04535
Ab	201F	GLU	-	insertion	UNP P04535
Ab	201G	ASP	-	insertion	UNP P04535
Ab	201H	GLU	-	insertion	UNP P04535
Ab	201I	ASP	-	insertion	UNP P04535
Ac	201A	ASP	-	insertion	UNP P04535
Ac	201B	GLU	-	insertion	UNP P04535
Ac	201C	ASP	-	insertion	UNP P04535
Ac	201D	GLU	-	insertion	UNP P04535
Ac	201E	ASP	-	insertion	UNP P04535
Ac	201F	GLU	-	insertion	UNP P04535
Ac	201G	ASP	-	insertion	UNP P04535

Continued on next page...

For Manuscript Review

Continued from previous page...

Chain	Residue	Modelled	Actual	Comment	Reference
Ac	201H	GLU	-	insertion	UNP P04535
Ac	201I	ASP	-	insertion	UNP P04535
Ad	201A	ASP	-	insertion	UNP P04535
Ad	201B	GLU	-	insertion	UNP P04535
Ad	201C	ASP	-	insertion	UNP P04535
Ad	201D	GLU	-	insertion	UNP P04535
Ad	201E	ASP	-	insertion	UNP P04535
Ad	201F	GLU	-	insertion	UNP P04535
Ad	201G	ASP	-	insertion	UNP P04535
Ad	201H	GLU	-	insertion	UNP P04535
Ad	201I	ASP	-	insertion	UNP P04535
Ae	201A	ASP	-	insertion	UNP P04535
Ae	201B	GLU	-	insertion	UNP P04535
Ae	201C	ASP	-	insertion	UNP P04535
Ae	201D	GLU	-	insertion	UNP P04535
Ae	201E	ASP	-	insertion	UNP P04535
Ae	201F	GLU	-	insertion	UNP P04535
Ae	201G	ASP	-	insertion	UNP P04535
Ae	201H	GLU	-	insertion	UNP P04535
Ae	201I	ASP	-	insertion	UNP P04535
Af	201A	ASP	-	insertion	UNP P04535
Af	201B	GLU	-	insertion	UNP P04535
Af	201C	ASP	-	insertion	UNP P04535
Af	201D	GLU	-	insertion	UNP P04535
Af	201E	ASP	-	insertion	UNP P04535
Af	201F	GLU	-	insertion	UNP P04535
Af	201G	ASP	-	insertion	UNP P04535
Af	201H	GLU	-	insertion	UNP P04535
Af	201I	ASP	-	insertion	UNP P04535
Ag	201A	ASP	-	insertion	UNP P04535
Ag	201B	GLU	-	insertion	UNP P04535
Ag	201C	ASP	-	insertion	UNP P04535
Ag	201D	GLU	-	insertion	UNP P04535
Ag	201E	ASP	-	insertion	UNP P04535
Ag	201F	GLU	-	insertion	UNP P04535
Ag	201G	ASP	-	insertion	UNP P04535
Ag	201H	GLU	-	insertion	UNP P04535
Ag	201I	ASP	-	insertion	UNP P04535
Ah	201A	ASP	-	insertion	UNP P04535
Ah	201B	GLU	-	insertion	UNP P04535
Ah	201C	ASP	-	insertion	UNP P04535
Ah	201D	GLU	-	insertion	UNP P04535

Continued on next page...

For Manuscript Review

Continued from previous page...

Chain	Residue	Modelled	Actual	Comment	Reference
Ah	201E	ASP	-	insertion	UNP P04535
Ah	201F	GLU	-	insertion	UNP P04535
Ah	201G	ASP	-	insertion	UNP P04535
Ah	201H	GLU	-	insertion	UNP P04535
Ah	201I	ASP	-	insertion	UNP P04535
Ai	201A	ASP	-	insertion	UNP P04535
Ai	201B	GLU	-	insertion	UNP P04535
Ai	201C	ASP	-	insertion	UNP P04535
Ai	201D	GLU	-	insertion	UNP P04535
Ai	201E	ASP	-	insertion	UNP P04535
Ai	201F	GLU	-	insertion	UNP P04535
Ai	201G	ASP	-	insertion	UNP P04535
Ai	201H	GLU	-	insertion	UNP P04535
Ai	201I	ASP	-	insertion	UNP P04535
Aj	201A	ASP	-	insertion	UNP P04535
Aj	201B	GLU	-	insertion	UNP P04535
Aj	201C	ASP	-	insertion	UNP P04535
Aj	201D	GLU	-	insertion	UNP P04535
Aj	201E	ASP	-	insertion	UNP P04535
Aj	201F	GLU	-	insertion	UNP P04535
Aj	201G	ASP	-	insertion	UNP P04535
Aj	201H	GLU	-	insertion	UNP P04535
Aj	201I	ASP	-	insertion	UNP P04535
Ak	201A	ASP	-	insertion	UNP P04535
Ak	201B	GLU	-	insertion	UNP P04535
Ak	201C	ASP	-	insertion	UNP P04535
Ak	201D	GLU	-	insertion	UNP P04535
Ak	201E	ASP	-	insertion	UNP P04535
Ak	201F	GLU	-	insertion	UNP P04535
Ak	201G	ASP	-	insertion	UNP P04535
Ak	201H	GLU	-	insertion	UNP P04535
Ak	201I	ASP	-	insertion	UNP P04535
Al	201A	ASP	-	insertion	UNP P04535
Al	201B	GLU	-	insertion	UNP P04535
Al	201C	ASP	-	insertion	UNP P04535
Al	201D	GLU	-	insertion	UNP P04535
Al	201E	ASP	-	insertion	UNP P04535
Al	201F	GLU	-	insertion	UNP P04535
Al	201G	ASP	-	insertion	UNP P04535
Al	201H	GLU	-	insertion	UNP P04535
Al	201I	ASP	-	insertion	UNP P04535
Am	201A	ASP	-	insertion	UNP P04535

Continued on next page...

For Manuscript Review

Continued from previous page...

Chain	Residue	Modelled	Actual	Comment	Reference
Am	201B	GLU	-	insertion	UNP P04535
Am	201C	ASP	-	insertion	UNP P04535
Am	201D	GLU	-	insertion	UNP P04535
Am	201E	ASP	-	insertion	UNP P04535
Am	201F	GLU	-	insertion	UNP P04535
Am	201G	ASP	-	insertion	UNP P04535
Am	201H	GLU	-	insertion	UNP P04535
Am	201I	ASP	-	insertion	UNP P04535
G	201A	ASP	-	insertion	UNP P04535
G	201B	GLU	-	insertion	UNP P04535
G	201C	ASP	-	insertion	UNP P04535
G	201D	GLU	-	insertion	UNP P04535
G	201E	ASP	-	insertion	UNP P04535
G	201F	GLU	-	insertion	UNP P04535
G	201G	ASP	-	insertion	UNP P04535
G	201H	GLU	-	insertion	UNP P04535
G	201I	ASP	-	insertion	UNP P04535
H	201A	ASP	-	insertion	UNP P04535
H	201B	GLU	-	insertion	UNP P04535
H	201C	ASP	-	insertion	UNP P04535
H	201D	GLU	-	insertion	UNP P04535
H	201E	ASP	-	insertion	UNP P04535
H	201F	GLU	-	insertion	UNP P04535
H	201G	ASP	-	insertion	UNP P04535
H	201H	GLU	-	insertion	UNP P04535
H	201I	ASP	-	insertion	UNP P04535
J	201A	ASP	-	insertion	UNP P04535
J	201B	GLU	-	insertion	UNP P04535
J	201C	ASP	-	insertion	UNP P04535
J	201D	GLU	-	insertion	UNP P04535
J	201E	ASP	-	insertion	UNP P04535
J	201F	GLU	-	insertion	UNP P04535
J	201G	ASP	-	insertion	UNP P04535
J	201H	GLU	-	insertion	UNP P04535
J	201I	ASP	-	insertion	UNP P04535
K	201A	ASP	-	insertion	UNP P04535
K	201B	GLU	-	insertion	UNP P04535
K	201C	ASP	-	insertion	UNP P04535
K	201D	GLU	-	insertion	UNP P04535
K	201E	ASP	-	insertion	UNP P04535
K	201F	GLU	-	insertion	UNP P04535
K	201G	ASP	-	insertion	UNP P04535

Continued on next page...

For Manuscript Review

Continued from previous page...

Chain	Residue	Modelled	Actual	Comment	Reference
K	201H	GLU	-	insertion	UNP P04535
K	201I	ASP	-	insertion	UNP P04535
L	201A	ASP	-	insertion	UNP P04535
L	201B	GLU	-	insertion	UNP P04535
L	201C	ASP	-	insertion	UNP P04535
L	201D	GLU	-	insertion	UNP P04535
L	201E	ASP	-	insertion	UNP P04535
L	201F	GLU	-	insertion	UNP P04535
L	201G	ASP	-	insertion	UNP P04535
L	201H	GLU	-	insertion	UNP P04535
L	201I	ASP	-	insertion	UNP P04535
M	201A	ASP	-	insertion	UNP P04535
M	201B	GLU	-	insertion	UNP P04535
M	201C	ASP	-	insertion	UNP P04535
M	201D	GLU	-	insertion	UNP P04535
M	201E	ASP	-	insertion	UNP P04535
M	201F	GLU	-	insertion	UNP P04535
M	201G	ASP	-	insertion	UNP P04535
M	201H	GLU	-	insertion	UNP P04535
M	201I	ASP	-	insertion	UNP P04535
N	201A	ASP	-	insertion	UNP P04535
N	201B	GLU	-	insertion	UNP P04535
N	201C	ASP	-	insertion	UNP P04535
N	201D	GLU	-	insertion	UNP P04535
N	201E	ASP	-	insertion	UNP P04535
N	201F	GLU	-	insertion	UNP P04535
N	201G	ASP	-	insertion	UNP P04535
N	201H	GLU	-	insertion	UNP P04535
N	201I	ASP	-	insertion	UNP P04535
O	201A	ASP	-	insertion	UNP P04535
O	201B	GLU	-	insertion	UNP P04535
O	201C	ASP	-	insertion	UNP P04535
O	201D	GLU	-	insertion	UNP P04535
O	201E	ASP	-	insertion	UNP P04535
O	201F	GLU	-	insertion	UNP P04535
O	201G	ASP	-	insertion	UNP P04535
O	201H	GLU	-	insertion	UNP P04535
O	201I	ASP	-	insertion	UNP P04535
P	201A	ASP	-	insertion	UNP P04535
P	201B	GLU	-	insertion	UNP P04535
P	201C	ASP	-	insertion	UNP P04535
P	201D	GLU	-	insertion	UNP P04535

Continued on next page...

For Manuscript Review

Continued from previous page...

Chain	Residue	Modelled	Actual	Comment	Reference
P	201E	ASP	-	insertion	UNP P04535
P	201F	GLU	-	insertion	UNP P04535
P	201G	ASP	-	insertion	UNP P04535
P	201H	GLU	-	insertion	UNP P04535
P	201I	ASP	-	insertion	UNP P04535
Q	201A	ASP	-	insertion	UNP P04535
Q	201B	GLU	-	insertion	UNP P04535
Q	201C	ASP	-	insertion	UNP P04535
Q	201D	GLU	-	insertion	UNP P04535
Q	201E	ASP	-	insertion	UNP P04535
Q	201F	GLU	-	insertion	UNP P04535
Q	201G	ASP	-	insertion	UNP P04535
Q	201H	GLU	-	insertion	UNP P04535
Q	201I	ASP	-	insertion	UNP P04535
R	201A	ASP	-	insertion	UNP P04535
R	201B	GLU	-	insertion	UNP P04535
R	201C	ASP	-	insertion	UNP P04535
R	201D	GLU	-	insertion	UNP P04535
R	201E	ASP	-	insertion	UNP P04535
R	201F	GLU	-	insertion	UNP P04535
R	201G	ASP	-	insertion	UNP P04535
R	201H	GLU	-	insertion	UNP P04535
R	201I	ASP	-	insertion	UNP P04535
S	201A	ASP	-	insertion	UNP P04535
S	201B	GLU	-	insertion	UNP P04535
S	201C	ASP	-	insertion	UNP P04535
S	201D	GLU	-	insertion	UNP P04535
S	201E	ASP	-	insertion	UNP P04535
S	201F	GLU	-	insertion	UNP P04535
S	201G	ASP	-	insertion	UNP P04535
S	201H	GLU	-	insertion	UNP P04535
S	201I	ASP	-	insertion	UNP P04535
T	201A	ASP	-	insertion	UNP P04535
T	201B	GLU	-	insertion	UNP P04535
T	201C	ASP	-	insertion	UNP P04535
T	201D	GLU	-	insertion	UNP P04535
T	201E	ASP	-	insertion	UNP P04535
T	201F	GLU	-	insertion	UNP P04535
T	201G	ASP	-	insertion	UNP P04535
T	201H	GLU	-	insertion	UNP P04535
T	201I	ASP	-	insertion	UNP P04535
U	201A	ASP	-	insertion	UNP P04535

Continued on next page...

For Manuscript Review

Continued from previous page...

Chain	Residue	Modelled	Actual	Comment	Reference
U	201B	GLU	-	insertion	UNP P04535
U	201C	ASP	-	insertion	UNP P04535
U	201D	GLU	-	insertion	UNP P04535
U	201E	ASP	-	insertion	UNP P04535
U	201F	GLU	-	insertion	UNP P04535
U	201G	ASP	-	insertion	UNP P04535
U	201H	GLU	-	insertion	UNP P04535
U	201I	ASP	-	insertion	UNP P04535
V	201A	ASP	-	insertion	UNP P04535
V	201B	GLU	-	insertion	UNP P04535
V	201C	ASP	-	insertion	UNP P04535
V	201D	GLU	-	insertion	UNP P04535
V	201E	ASP	-	insertion	UNP P04535
V	201F	GLU	-	insertion	UNP P04535
V	201G	ASP	-	insertion	UNP P04535
V	201H	GLU	-	insertion	UNP P04535
V	201I	ASP	-	insertion	UNP P04535
W	201A	ASP	-	insertion	UNP P04535
W	201B	GLU	-	insertion	UNP P04535
W	201C	ASP	-	insertion	UNP P04535
W	201D	GLU	-	insertion	UNP P04535
W	201E	ASP	-	insertion	UNP P04535
W	201F	GLU	-	insertion	UNP P04535
W	201G	ASP	-	insertion	UNP P04535
W	201H	GLU	-	insertion	UNP P04535
W	201I	ASP	-	insertion	UNP P04535
X	201A	ASP	-	insertion	UNP P04535
X	201B	GLU	-	insertion	UNP P04535
X	201C	ASP	-	insertion	UNP P04535
X	201D	GLU	-	insertion	UNP P04535
X	201E	ASP	-	insertion	UNP P04535
X	201F	GLU	-	insertion	UNP P04535
X	201G	ASP	-	insertion	UNP P04535
X	201H	GLU	-	insertion	UNP P04535
X	201I	ASP	-	insertion	UNP P04535
Y	201A	ASP	-	insertion	UNP P04535
Y	201B	GLU	-	insertion	UNP P04535
Y	201C	ASP	-	insertion	UNP P04535
Y	201D	GLU	-	insertion	UNP P04535
Y	201E	ASP	-	insertion	UNP P04535
Y	201F	GLU	-	insertion	UNP P04535
Y	201G	ASP	-	insertion	UNP P04535

Continued on next page...

For Manuscript Review

Continued from previous page...

Chain	Residue	Modelled	Actual	Comment	Reference
Y	201H	GLU	-	insertion	UNP P04535
Y	201I	ASP	-	insertion	UNP P04535
Z	201A	ASP	-	insertion	UNP P04535
Z	201B	GLU	-	insertion	UNP P04535
Z	201C	ASP	-	insertion	UNP P04535
Z	201D	GLU	-	insertion	UNP P04535
Z	201E	ASP	-	insertion	UNP P04535
Z	201F	GLU	-	insertion	UNP P04535
Z	201G	ASP	-	insertion	UNP P04535
Z	201H	GLU	-	insertion	UNP P04535
Z	201I	ASP	-	insertion	UNP P04535
b	201A	ASP	-	insertion	UNP P04535
b	201B	GLU	-	insertion	UNP P04535
b	201C	ASP	-	insertion	UNP P04535
b	201D	GLU	-	insertion	UNP P04535
b	201E	ASP	-	insertion	UNP P04535
b	201F	GLU	-	insertion	UNP P04535
b	201G	ASP	-	insertion	UNP P04535
b	201H	GLU	-	insertion	UNP P04535
b	201I	ASP	-	insertion	UNP P04535
c	201A	ASP	-	insertion	UNP P04535
c	201B	GLU	-	insertion	UNP P04535
c	201C	ASP	-	insertion	UNP P04535
c	201D	GLU	-	insertion	UNP P04535
c	201E	ASP	-	insertion	UNP P04535
c	201F	GLU	-	insertion	UNP P04535
c	201G	ASP	-	insertion	UNP P04535
c	201H	GLU	-	insertion	UNP P04535
c	201I	ASP	-	insertion	UNP P04535
d	201A	ASP	-	insertion	UNP P04535
d	201B	GLU	-	insertion	UNP P04535
d	201C	ASP	-	insertion	UNP P04535
d	201D	GLU	-	insertion	UNP P04535
d	201E	ASP	-	insertion	UNP P04535
d	201F	GLU	-	insertion	UNP P04535
d	201G	ASP	-	insertion	UNP P04535
d	201H	GLU	-	insertion	UNP P04535
d	201I	ASP	-	insertion	UNP P04535
e	201A	ASP	-	insertion	UNP P04535
e	201B	GLU	-	insertion	UNP P04535
e	201C	ASP	-	insertion	UNP P04535
e	201D	GLU	-	insertion	UNP P04535

Continued on next page...

For Manuscript Review

Continued from previous page...

Chain	Residue	Modelled	Actual	Comment	Reference
e	201E	ASP	-	insertion	UNP P04535
e	201F	GLU	-	insertion	UNP P04535
e	201G	ASP	-	insertion	UNP P04535
e	201H	GLU	-	insertion	UNP P04535
e	201I	ASP	-	insertion	UNP P04535
f	201A	ASP	-	insertion	UNP P04535
f	201B	GLU	-	insertion	UNP P04535
f	201C	ASP	-	insertion	UNP P04535
f	201D	GLU	-	insertion	UNP P04535
f	201E	ASP	-	insertion	UNP P04535
f	201F	GLU	-	insertion	UNP P04535
f	201G	ASP	-	insertion	UNP P04535
f	201H	GLU	-	insertion	UNP P04535
f	201I	ASP	-	insertion	UNP P04535
g	201A	ASP	-	insertion	UNP P04535
g	201B	GLU	-	insertion	UNP P04535
g	201C	ASP	-	insertion	UNP P04535
g	201D	GLU	-	insertion	UNP P04535
g	201E	ASP	-	insertion	UNP P04535
g	201F	GLU	-	insertion	UNP P04535
g	201G	ASP	-	insertion	UNP P04535
g	201H	GLU	-	insertion	UNP P04535
g	201I	ASP	-	insertion	UNP P04535
h	201A	ASP	-	insertion	UNP P04535
h	201B	GLU	-	insertion	UNP P04535
h	201C	ASP	-	insertion	UNP P04535
h	201D	GLU	-	insertion	UNP P04535
h	201E	ASP	-	insertion	UNP P04535
h	201F	GLU	-	insertion	UNP P04535
h	201G	ASP	-	insertion	UNP P04535
h	201H	GLU	-	insertion	UNP P04535
h	201I	ASP	-	insertion	UNP P04535
i	201A	ASP	-	insertion	UNP P04535
i	201B	GLU	-	insertion	UNP P04535
i	201C	ASP	-	insertion	UNP P04535
i	201D	GLU	-	insertion	UNP P04535
i	201E	ASP	-	insertion	UNP P04535
i	201F	GLU	-	insertion	UNP P04535
i	201G	ASP	-	insertion	UNP P04535
i	201H	GLU	-	insertion	UNP P04535
i	201I	ASP	-	insertion	UNP P04535
j	201A	ASP	-	insertion	UNP P04535

Continued on next page...

For Manuscript Review

Continued from previous page...

Chain	Residue	Modelled	Actual	Comment	Reference
j	201B	GLU	-	insertion	UNP P04535
j	201C	ASP	-	insertion	UNP P04535
j	201D	GLU	-	insertion	UNP P04535
j	201E	ASP	-	insertion	UNP P04535
j	201F	GLU	-	insertion	UNP P04535
j	201G	ASP	-	insertion	UNP P04535
j	201H	GLU	-	insertion	UNP P04535
j	201I	ASP	-	insertion	UNP P04535
k	201A	ASP	-	insertion	UNP P04535
k	201B	GLU	-	insertion	UNP P04535
k	201C	ASP	-	insertion	UNP P04535
k	201D	GLU	-	insertion	UNP P04535
k	201E	ASP	-	insertion	UNP P04535
k	201F	GLU	-	insertion	UNP P04535
k	201G	ASP	-	insertion	UNP P04535
k	201H	GLU	-	insertion	UNP P04535
k	201I	ASP	-	insertion	UNP P04535
l	201A	ASP	-	insertion	UNP P04535
l	201B	GLU	-	insertion	UNP P04535
l	201C	ASP	-	insertion	UNP P04535
l	201D	GLU	-	insertion	UNP P04535
l	201E	ASP	-	insertion	UNP P04535
l	201F	GLU	-	insertion	UNP P04535
l	201G	ASP	-	insertion	UNP P04535
l	201H	GLU	-	insertion	UNP P04535
l	201I	ASP	-	insertion	UNP P04535
m	201A	ASP	-	insertion	UNP P04535
m	201B	GLU	-	insertion	UNP P04535
m	201C	ASP	-	insertion	UNP P04535
m	201D	GLU	-	insertion	UNP P04535
m	201E	ASP	-	insertion	UNP P04535
m	201F	GLU	-	insertion	UNP P04535
m	201G	ASP	-	insertion	UNP P04535
m	201H	GLU	-	insertion	UNP P04535
m	201I	ASP	-	insertion	UNP P04535
n	201A	ASP	-	insertion	UNP P04535
n	201B	GLU	-	insertion	UNP P04535
n	201C	ASP	-	insertion	UNP P04535
n	201D	GLU	-	insertion	UNP P04535
n	201E	ASP	-	insertion	UNP P04535
n	201F	GLU	-	insertion	UNP P04535
n	201G	ASP	-	insertion	UNP P04535

Continued on next page...

For Manuscript Review

Continued from previous page...

Chain	Residue	Modelled	Actual	Comment	Reference
n	201H	GLU	-	insertion	UNP P04535
n	201I	ASP	-	insertion	UNP P04535
o	201A	ASP	-	insertion	UNP P04535
o	201B	GLU	-	insertion	UNP P04535
o	201C	ASP	-	insertion	UNP P04535
o	201D	GLU	-	insertion	UNP P04535
o	201E	ASP	-	insertion	UNP P04535
o	201F	GLU	-	insertion	UNP P04535
o	201G	ASP	-	insertion	UNP P04535
o	201H	GLU	-	insertion	UNP P04535
o	201I	ASP	-	insertion	UNP P04535
p	201A	ASP	-	insertion	UNP P04535
p	201B	GLU	-	insertion	UNP P04535
p	201C	ASP	-	insertion	UNP P04535
p	201D	GLU	-	insertion	UNP P04535
p	201E	ASP	-	insertion	UNP P04535
p	201F	GLU	-	insertion	UNP P04535
p	201G	ASP	-	insertion	UNP P04535
p	201H	GLU	-	insertion	UNP P04535
p	201I	ASP	-	insertion	UNP P04535
q	201A	ASP	-	insertion	UNP P04535
q	201B	GLU	-	insertion	UNP P04535
q	201C	ASP	-	insertion	UNP P04535
q	201D	GLU	-	insertion	UNP P04535
q	201E	ASP	-	insertion	UNP P04535
q	201F	GLU	-	insertion	UNP P04535
q	201G	ASP	-	insertion	UNP P04535
q	201H	GLU	-	insertion	UNP P04535
q	201I	ASP	-	insertion	UNP P04535
r	201A	ASP	-	insertion	UNP P04535
r	201B	GLU	-	insertion	UNP P04535
r	201C	ASP	-	insertion	UNP P04535
r	201D	GLU	-	insertion	UNP P04535
r	201E	ASP	-	insertion	UNP P04535
r	201F	GLU	-	insertion	UNP P04535
r	201G	ASP	-	insertion	UNP P04535
r	201H	GLU	-	insertion	UNP P04535
r	201I	ASP	-	insertion	UNP P04535
s	201A	ASP	-	insertion	UNP P04535
s	201B	GLU	-	insertion	UNP P04535
s	201C	ASP	-	insertion	UNP P04535
s	201D	GLU	-	insertion	UNP P04535

Continued on next page...

For Manuscript Review

Continued from previous page...

Chain	Residue	Modelled	Actual	Comment	Reference
s	201E	ASP	-	insertion	UNP P04535
s	201F	GLU	-	insertion	UNP P04535
s	201G	ASP	-	insertion	UNP P04535
s	201H	GLU	-	insertion	UNP P04535
s	201I	ASP	-	insertion	UNP P04535
t	201A	ASP	-	insertion	UNP P04535
t	201B	GLU	-	insertion	UNP P04535
t	201C	ASP	-	insertion	UNP P04535
t	201D	GLU	-	insertion	UNP P04535
t	201E	ASP	-	insertion	UNP P04535
t	201F	GLU	-	insertion	UNP P04535
t	201G	ASP	-	insertion	UNP P04535
t	201H	GLU	-	insertion	UNP P04535
t	201I	ASP	-	insertion	UNP P04535
u	201A	ASP	-	insertion	UNP P04535
u	201B	GLU	-	insertion	UNP P04535
u	201C	ASP	-	insertion	UNP P04535
u	201D	GLU	-	insertion	UNP P04535
u	201E	ASP	-	insertion	UNP P04535
u	201F	GLU	-	insertion	UNP P04535
u	201G	ASP	-	insertion	UNP P04535
u	201H	GLU	-	insertion	UNP P04535
u	201I	ASP	-	insertion	UNP P04535
v	201A	ASP	-	insertion	UNP P04535
v	201B	GLU	-	insertion	UNP P04535
v	201C	ASP	-	insertion	UNP P04535
v	201D	GLU	-	insertion	UNP P04535
v	201E	ASP	-	insertion	UNP P04535
v	201F	GLU	-	insertion	UNP P04535
v	201G	ASP	-	insertion	UNP P04535
v	201H	GLU	-	insertion	UNP P04535
v	201I	ASP	-	insertion	UNP P04535
w	201A	ASP	-	insertion	UNP P04535
w	201B	GLU	-	insertion	UNP P04535
w	201C	ASP	-	insertion	UNP P04535
w	201D	GLU	-	insertion	UNP P04535
w	201E	ASP	-	insertion	UNP P04535
w	201F	GLU	-	insertion	UNP P04535
w	201G	ASP	-	insertion	UNP P04535
w	201H	GLU	-	insertion	UNP P04535
w	201I	ASP	-	insertion	UNP P04535
x	201A	ASP	-	insertion	UNP P04535

Continued on next page...

For Manuscript Review

Continued from previous page...

Chain	Residue	Modelled	Actual	Comment	Reference
x	201B	GLU	-	insertion	UNP P04535
x	201C	ASP	-	insertion	UNP P04535
x	201D	GLU	-	insertion	UNP P04535
x	201E	ASP	-	insertion	UNP P04535
x	201F	GLU	-	insertion	UNP P04535
x	201G	ASP	-	insertion	UNP P04535
x	201H	GLU	-	insertion	UNP P04535
x	201I	ASP	-	insertion	UNP P04535
y	201A	ASP	-	insertion	UNP P04535
y	201B	GLU	-	insertion	UNP P04535
y	201C	ASP	-	insertion	UNP P04535
y	201D	GLU	-	insertion	UNP P04535
y	201E	ASP	-	insertion	UNP P04535
y	201F	GLU	-	insertion	UNP P04535
y	201G	ASP	-	insertion	UNP P04535
y	201H	GLU	-	insertion	UNP P04535
y	201I	ASP	-	insertion	UNP P04535
z	201A	ASP	-	insertion	UNP P04535
z	201B	GLU	-	insertion	UNP P04535
z	201C	ASP	-	insertion	UNP P04535
z	201D	GLU	-	insertion	UNP P04535
z	201E	ASP	-	insertion	UNP P04535
z	201F	GLU	-	insertion	UNP P04535
z	201G	ASP	-	insertion	UNP P04535
z	201H	GLU	-	insertion	UNP P04535
z	201I	ASP	-	insertion	UNP P04535

- Molecule 2 is a protein called Mature capsid vertex protein.

Mol	Chain	Residues	Atoms					AltConf	Trace
2	A	415	Total 3208	C 2039	N 521	O 640	S 8	0	0
2	B	415	Total 3208	C 2039	N 521	O 640	S 8	0	0
2	C	415	Total 3208	C 2039	N 521	O 640	S 8	0	0
2	D	415	Total 3208	C 2039	N 521	O 640	S 8	0	0
2	a	415	Total 3208	C 2039	N 521	O 640	S 8	0	0

For Manuscript Review

3 Residue-property plots ⓘ

These plots are drawn for all protein, RNA, DNA and oligosaccharide chains in the entry. The first graphic for a chain summarises the proportions of the various outlier classes displayed in the second graphic. The second graphic shows the sequence view annotated by issues in geometry and atom inclusion in map density. Residues are color-coded according to the number of geometric quality criteria for which they contain at least one outlier: green = 0, yellow = 1, orange = 2 and red = 3 or more. A red diamond above a residue indicates a poor fit to the EM map for this residue (all-atom inclusion < 40%). Stretches of 2 or more consecutive residues without any outlier are shown as a green connector. Residues present in the sample, but not in the model, are shown in grey.

- Molecule 1: Mature major capsid protein

Chain 0:

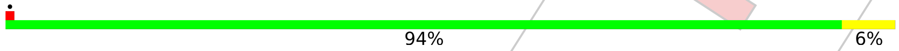

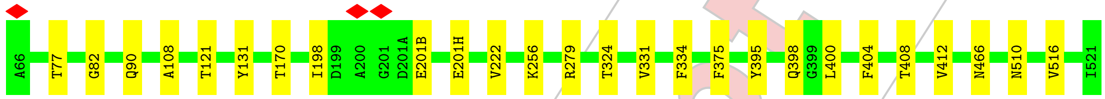

- Molecule 1: Mature major capsid protein

Chain 1:

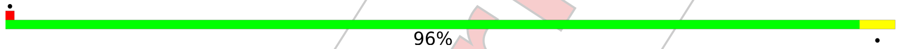

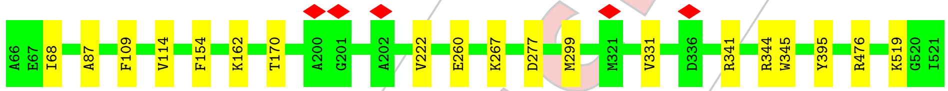

- Molecule 1: Mature major capsid protein

Chain 2:

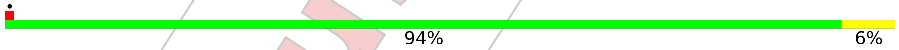

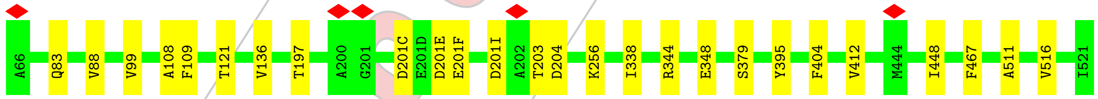

- Molecule 1: Mature major capsid protein

Chain 3:

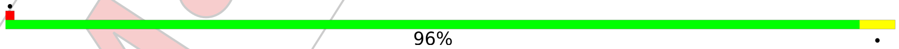

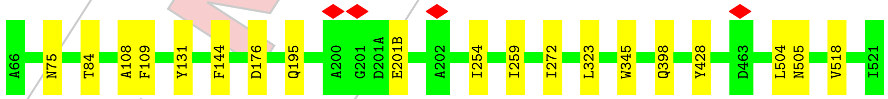

- Molecule 1: Mature major capsid protein

Chain 4:

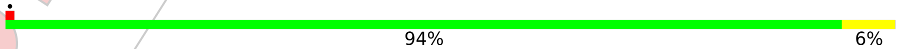

For Manuscript Review

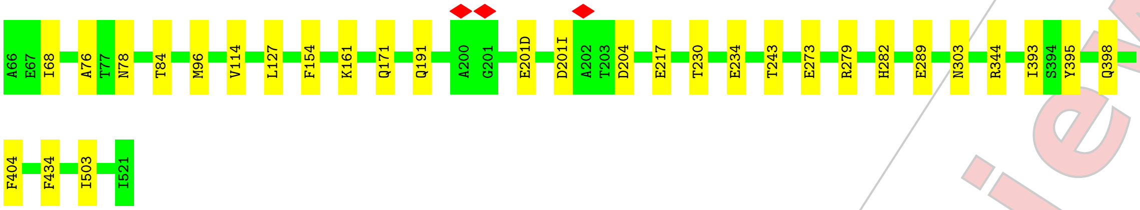

• Molecule 1: Mature major capsid protein

Chain 5:

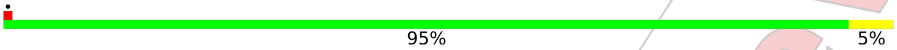

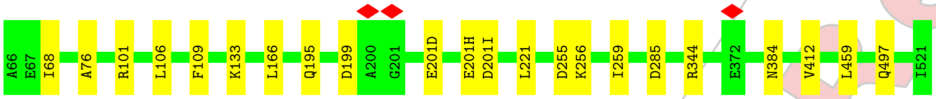

• Molecule 1: Mature major capsid protein

Chain 6:

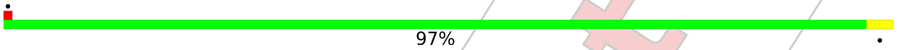

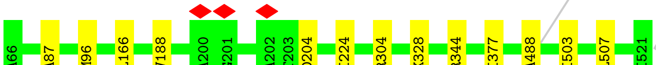

• Molecule 1: Mature major capsid protein

Chain 7:

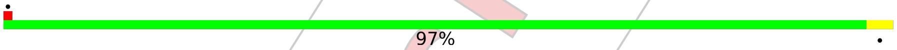

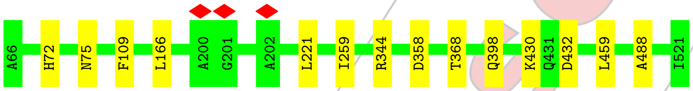

• Molecule 1: Mature major capsid protein

Chain 8:

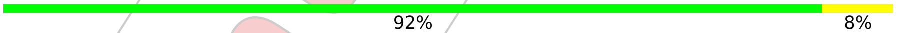

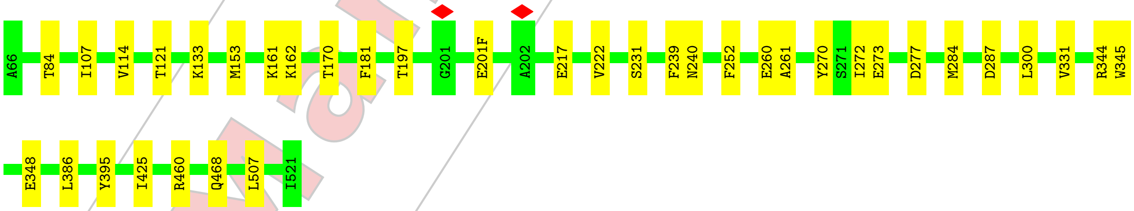

• Molecule 1: Mature major capsid protein

Chain 9:

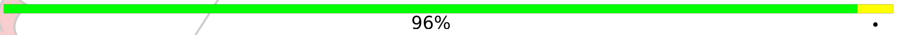

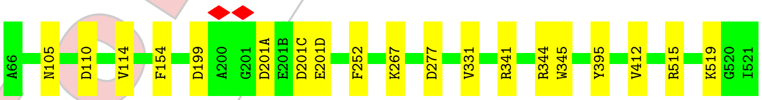

• Molecule 1: Mature major capsid protein

For Manuscript Review

Chain AA:

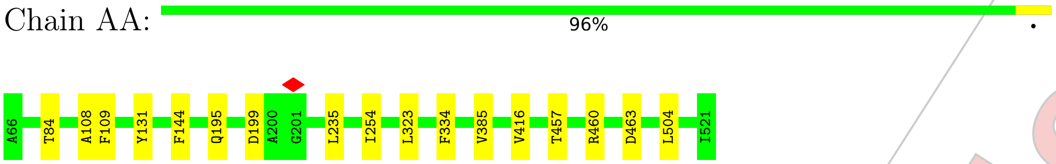

• Molecule 1: Mature major capsid protein

Chain AB:

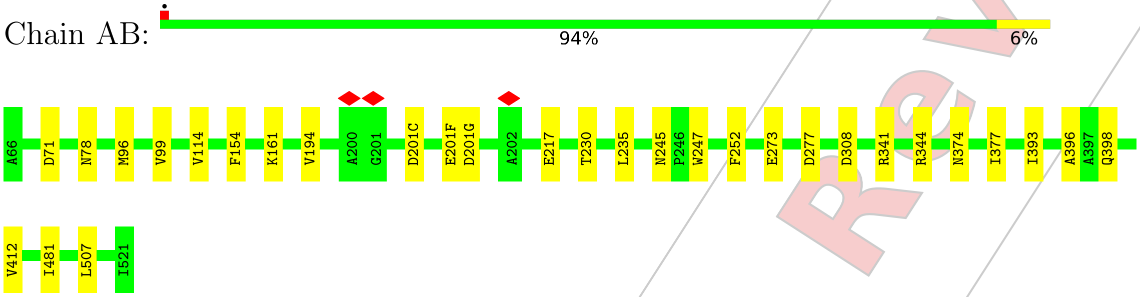

• Molecule 1: Mature major capsid protein

Chain AC:

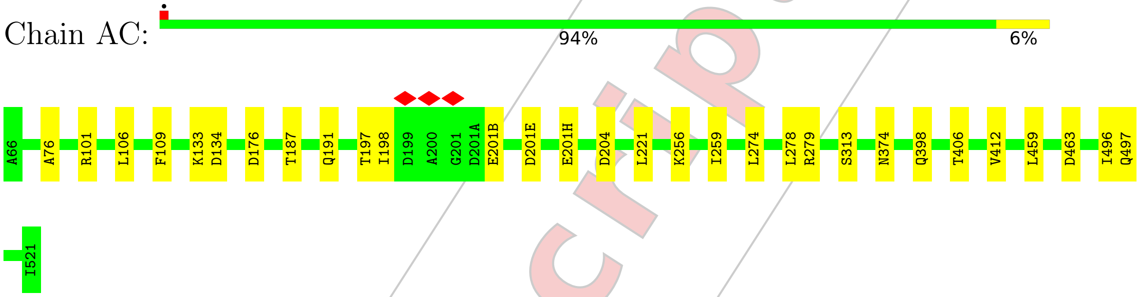

• Molecule 1: Mature major capsid protein

Chain AD:

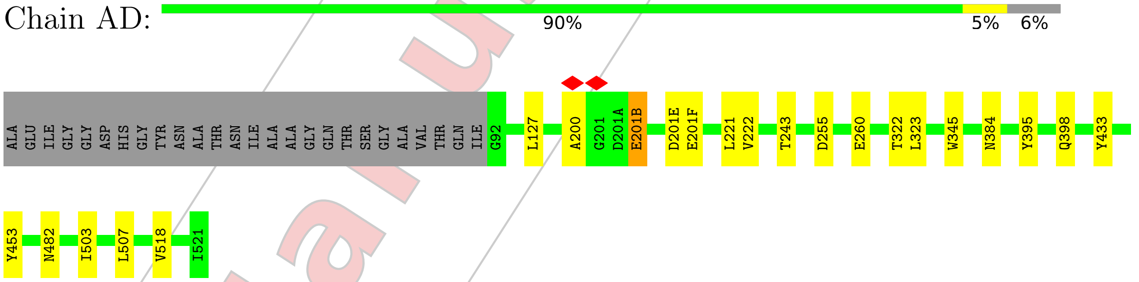

• Molecule 1: Mature major capsid protein

Chain AE:

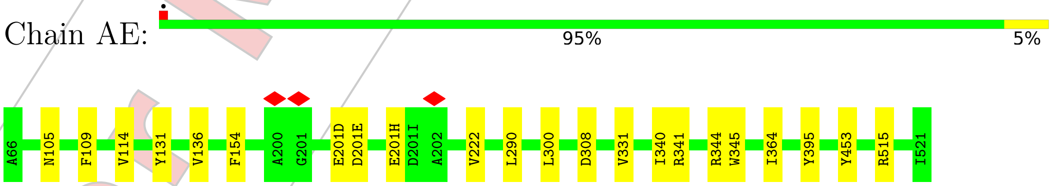

• Molecule 1: Mature major capsid protein

Chain AF:

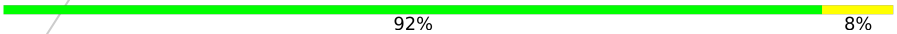

For Manuscript Review

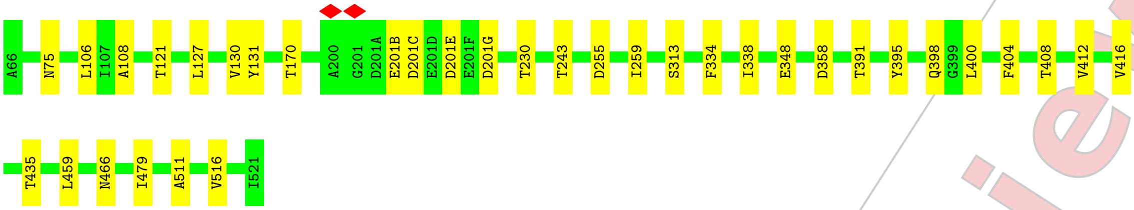

• Molecule 1: Mature major capsid protein

Chain AG:

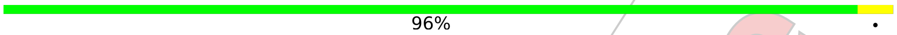

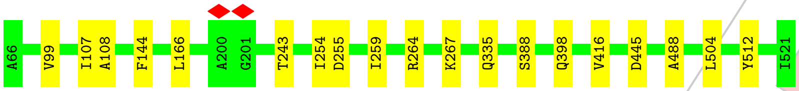

• Molecule 1: Mature major capsid protein

Chain AH:

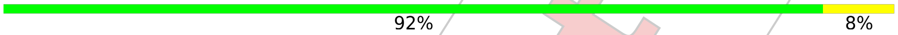

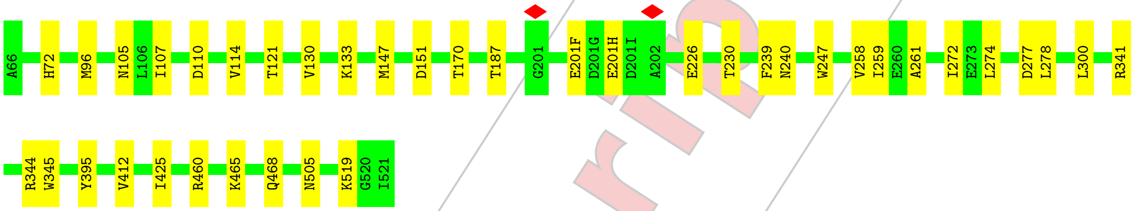

• Molecule 1: Mature major capsid protein

Chain AI:

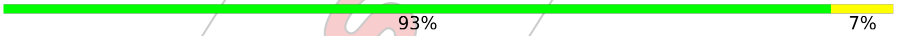

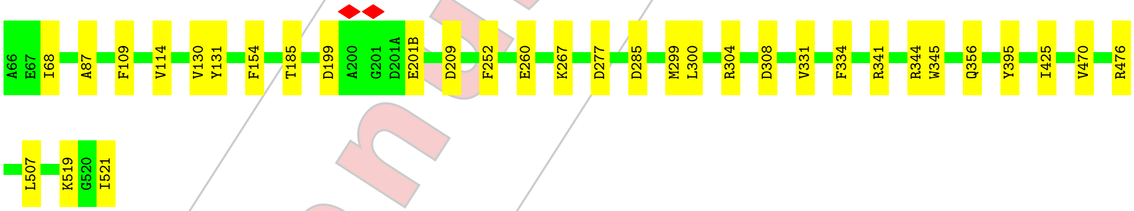

• Molecule 1: Mature major capsid protein

Chain AJ:

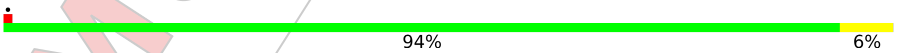

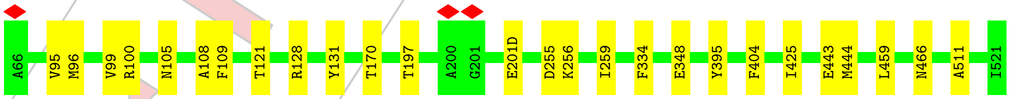

• Molecule 1: Mature major capsid protein

Chain AK:

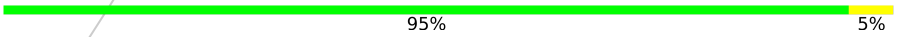

For Manuscript Review

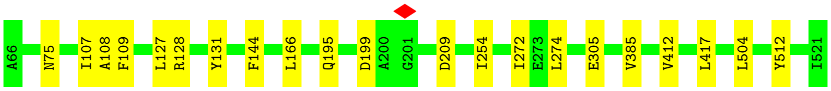

• Molecule 1: Mature major capsid protein

Chain AL:

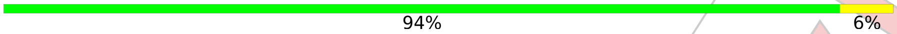

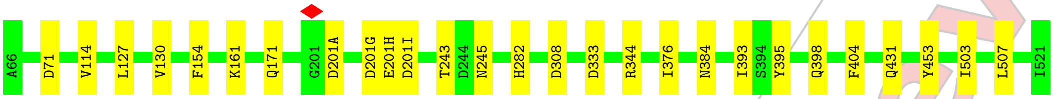

• Molecule 1: Mature major capsid protein

Chain AM:

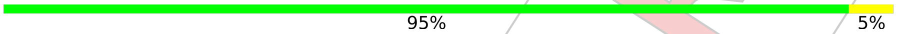

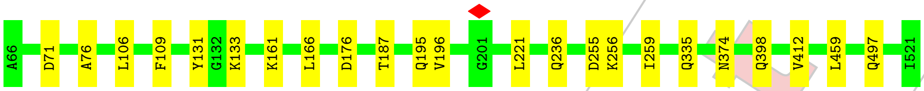

• Molecule 1: Mature major capsid protein

Chain AN:

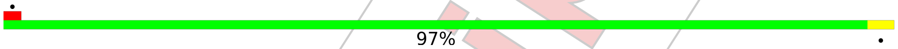

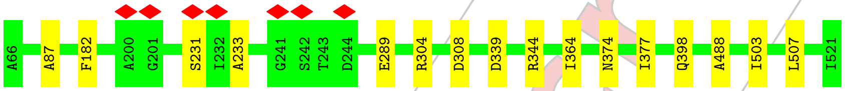

• Molecule 1: Mature major capsid protein

Chain AO:

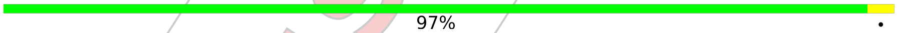

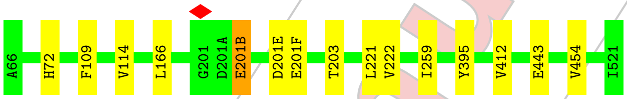

• Molecule 1: Mature major capsid protein

Chain AP:

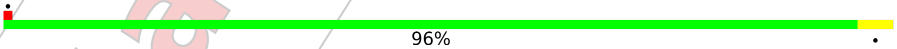

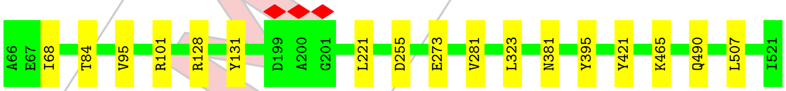

• Molecule 1: Mature major capsid protein

Chain AQ:

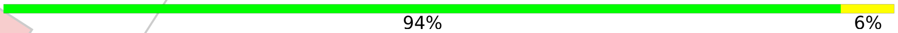

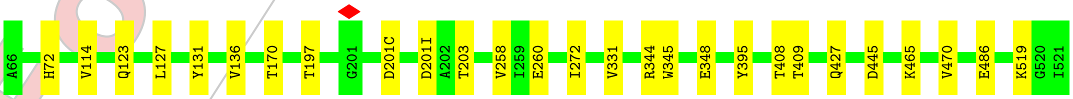

For Manuscript Review

- Molecule 1: Mature major capsid protein

Chain AR:

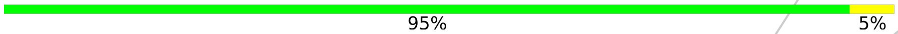

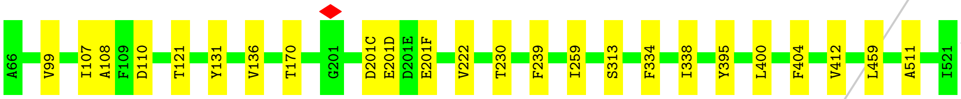

- Molecule 1: Mature major capsid protein

Chain AS:

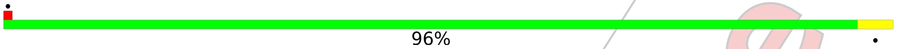

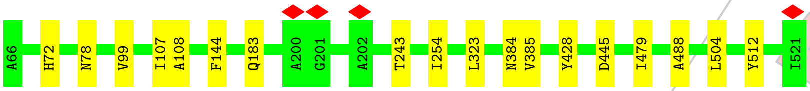

- Molecule 1: Mature major capsid protein

Chain AT:

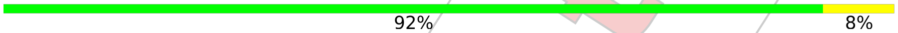

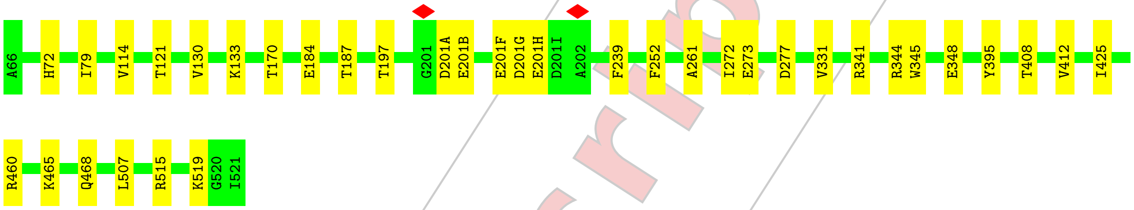

- Molecule 1: Mature major capsid protein

Chain AU:

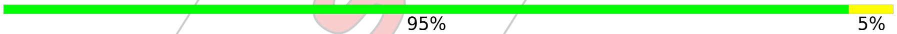

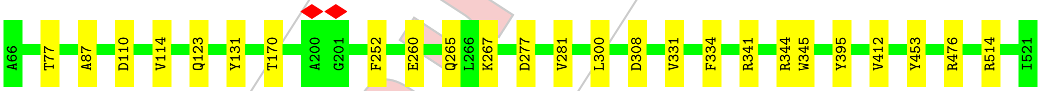

- Molecule 1: Mature major capsid protein

Chain AV:

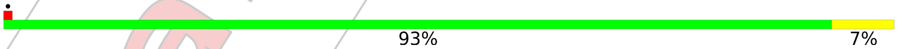

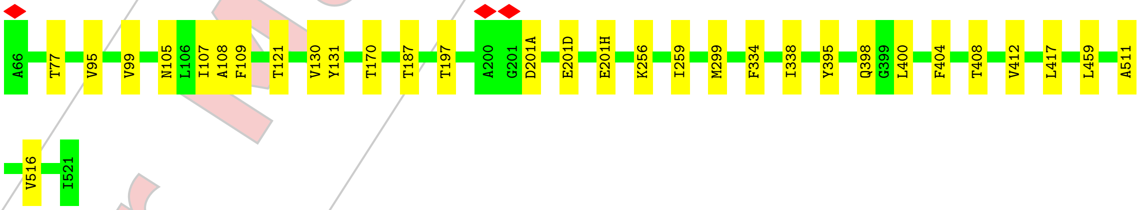

- Molecule 1: Mature major capsid protein

Chain AW:

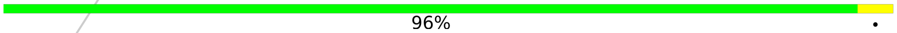

For Manuscript Review

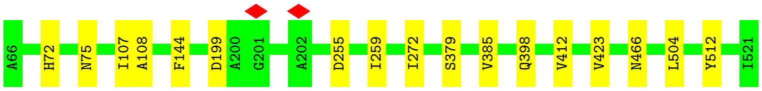

• Molecule 1: Mature major capsid protein

Chain AX:

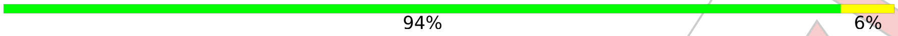

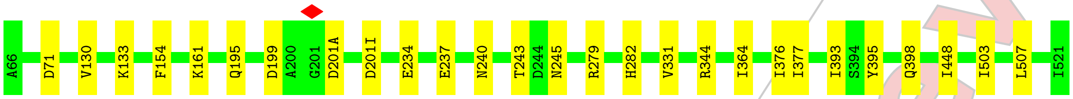

• Molecule 1: Mature major capsid protein

Chain AY:

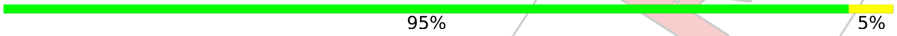

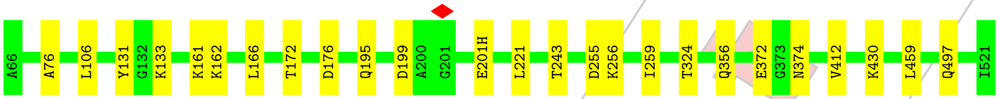

• Molecule 1: Mature major capsid protein

Chain AZ:

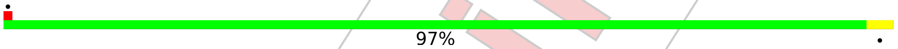

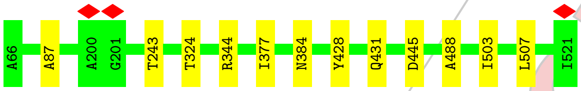

• Molecule 1: Mature major capsid protein

Chain Aa:

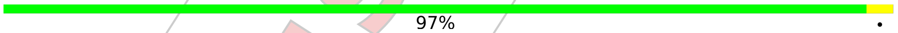

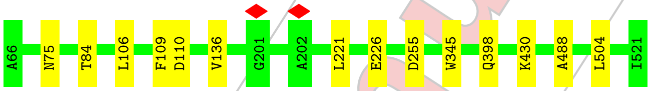

• Molecule 1: Mature major capsid protein

Chain Ab:

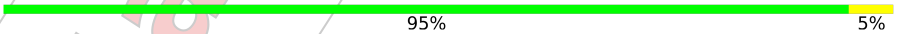

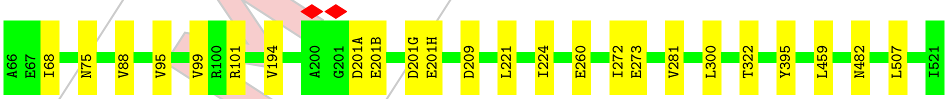

• Molecule 1: Mature major capsid protein

Chain Ac:

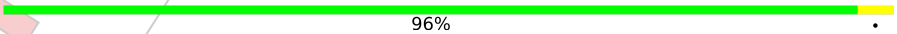

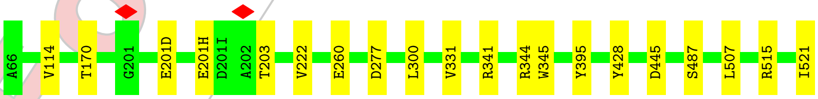

For Manuscript Review

- Molecule 1: Mature major capsid protein

Chain Ad:

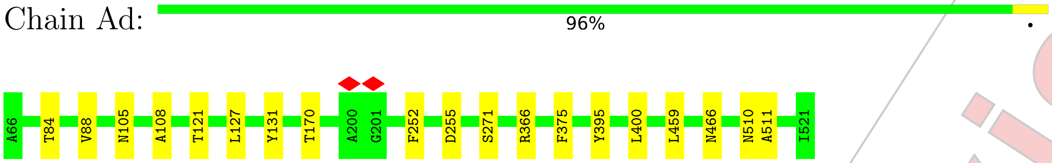

- Molecule 1: Mature major capsid protein

Chain Ae:

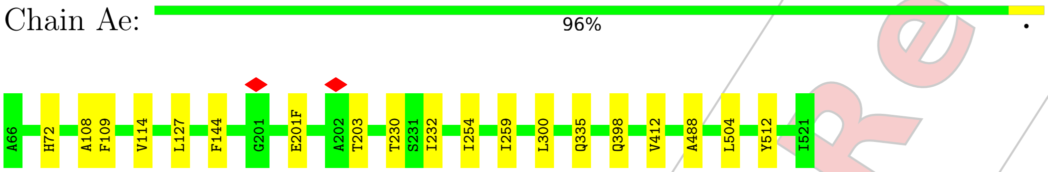

- Molecule 1: Mature major capsid protein

Chain Af:

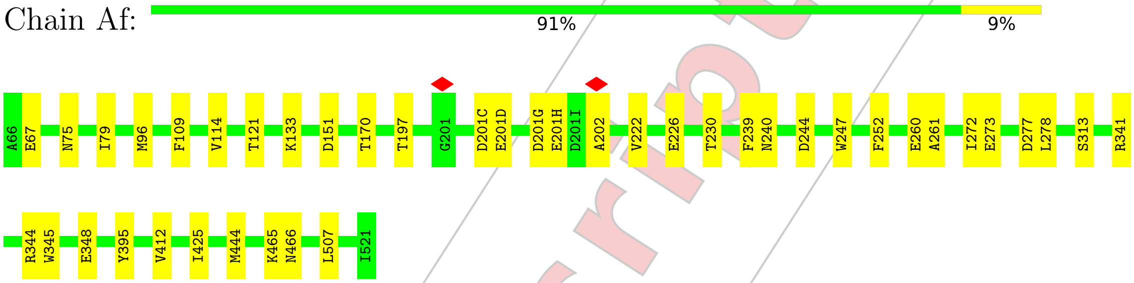

- Molecule 1: Mature major capsid protein

Chain Ag:

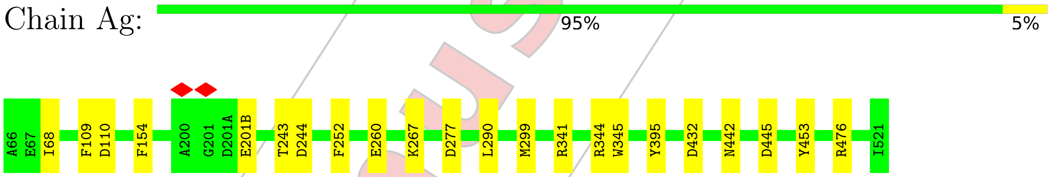

- Molecule 1: Mature major capsid protein

Chain Ah:

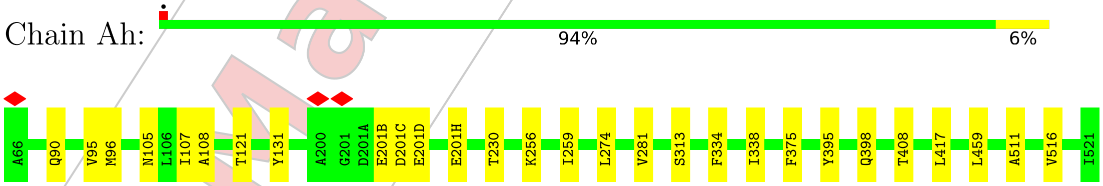

- Molecule 1: Mature major capsid protein

Chain Ai:

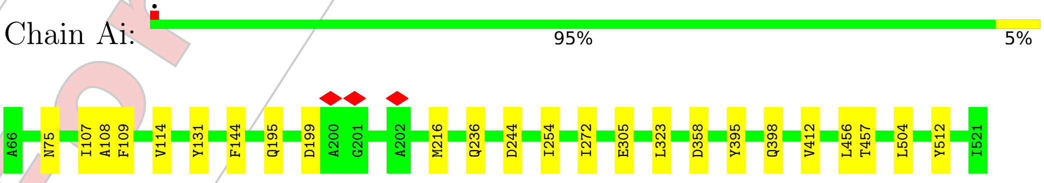

- Molecule 1: Mature major capsid protein

For Manuscript Review

Chain Aj:

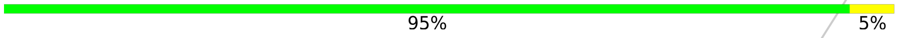

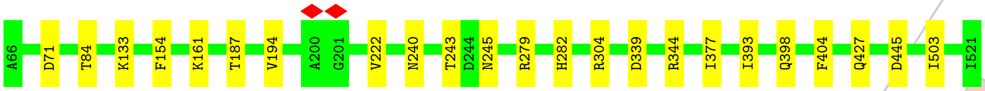

• Molecule 1: Mature major capsid protein

Chain Ak:

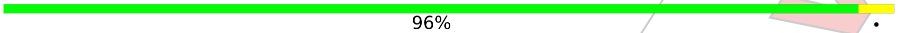

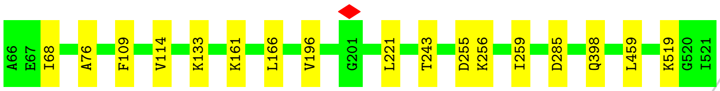

• Molecule 1: Mature major capsid protein

Chain Al:

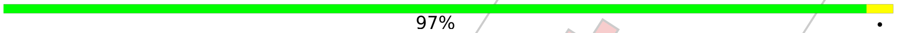

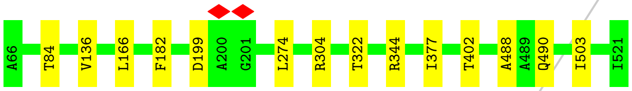

• Molecule 1: Mature major capsid protein

Chain Am:

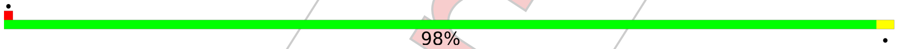

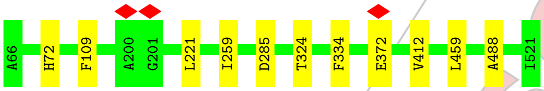

• Molecule 1: Mature major capsid protein

Chain G:

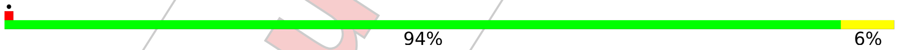

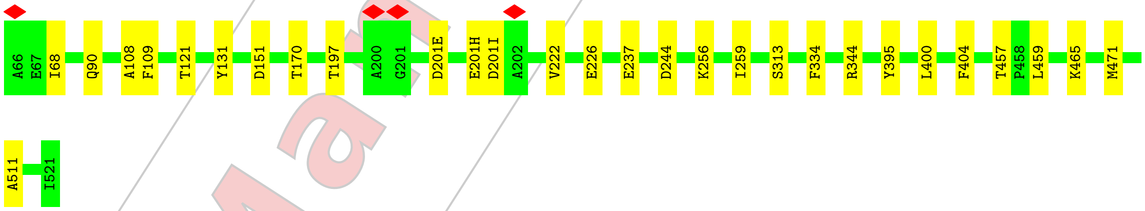

• Molecule 1: Mature major capsid protein

Chain H:

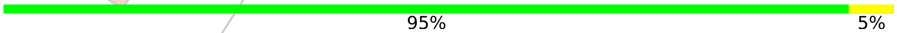

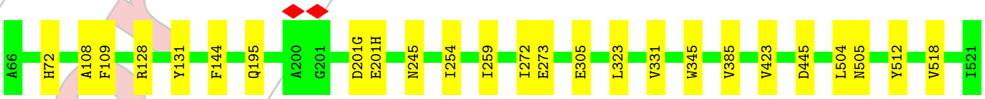

• Molecule 1: Mature major capsid protein

Chain J:

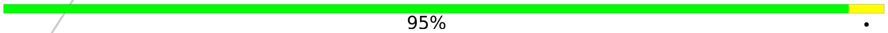

For Manuscript Review

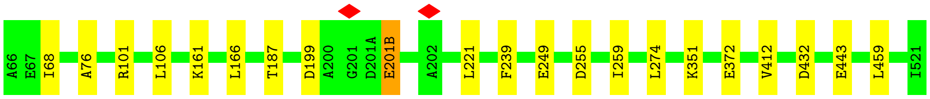

• Molecule 1: Mature major capsid protein

Chain K:

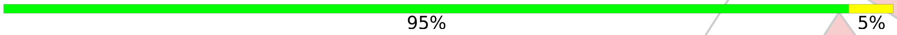

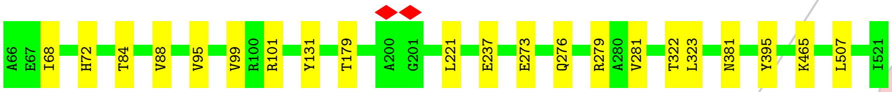

• Molecule 1: Mature major capsid protein

Chain L:

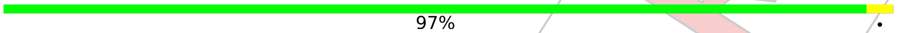

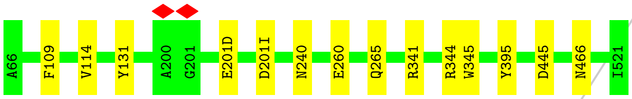

• Molecule 1: Mature major capsid protein

Chain M:

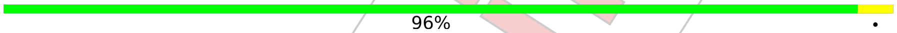

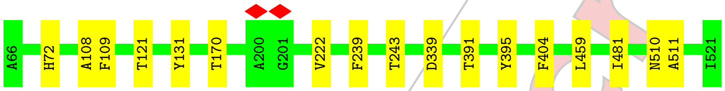

• Molecule 1: Mature major capsid protein

Chain N:

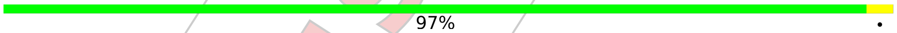

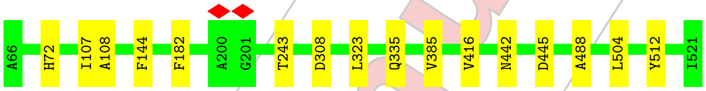

• Molecule 1: Mature major capsid protein

Chain O:

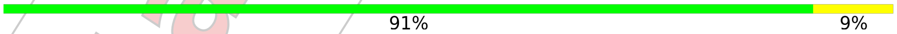

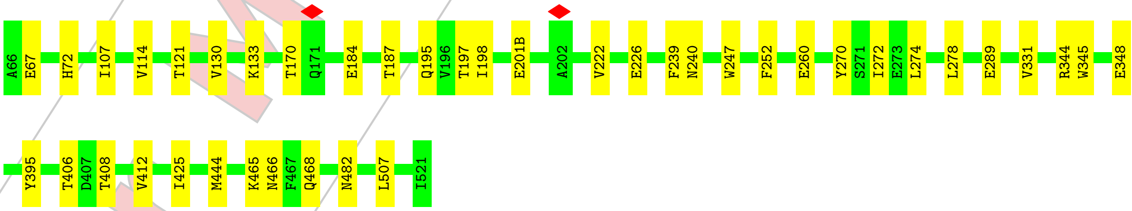

• Molecule 1: Mature major capsid protein

Chain P:

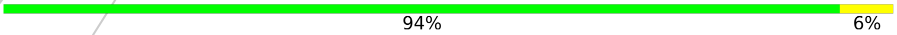

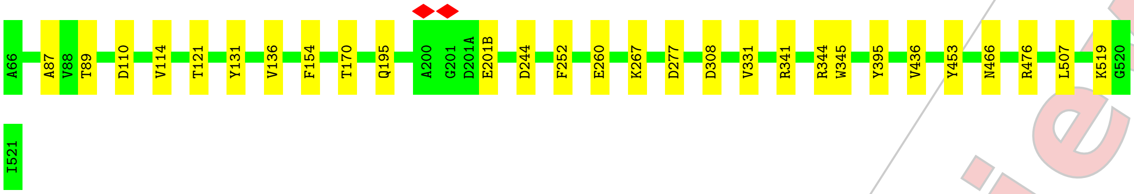

• Molecule 1: Mature major capsid protein

Chain Q:

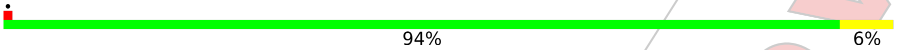

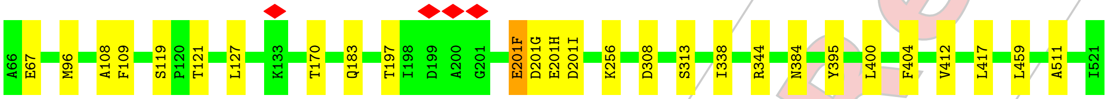

• Molecule 1: Mature major capsid protein

Chain R:

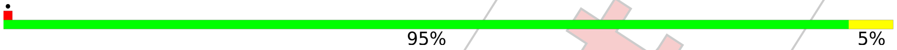

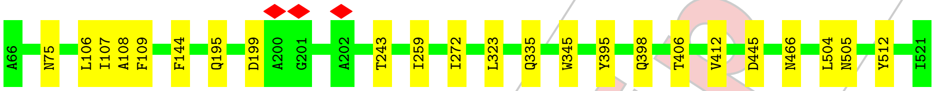

• Molecule 1: Mature major capsid protein

Chain S:

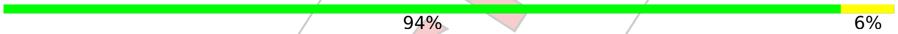

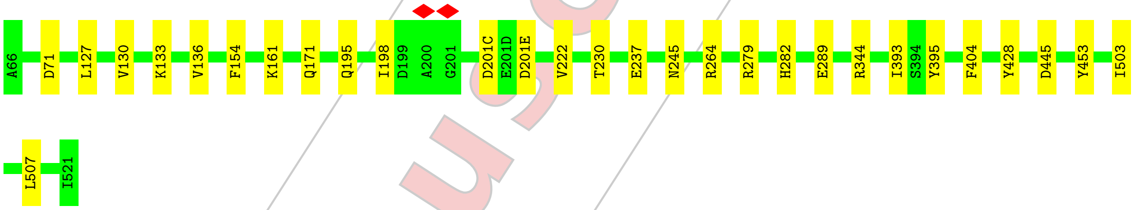

• Molecule 1: Mature major capsid protein

Chain T:

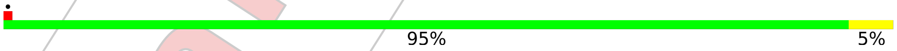

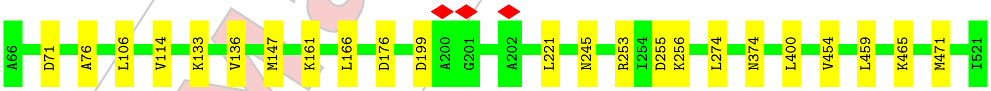

• Molecule 1: Mature major capsid protein

Chain U:

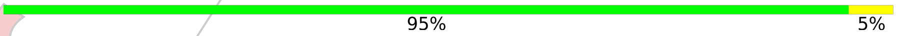

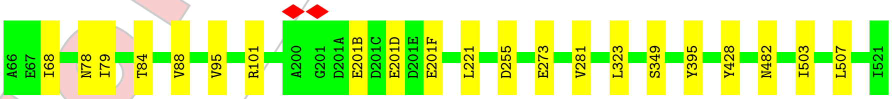

• Molecule 1: Mature major capsid protein

For Manuscript Review

Chain V:

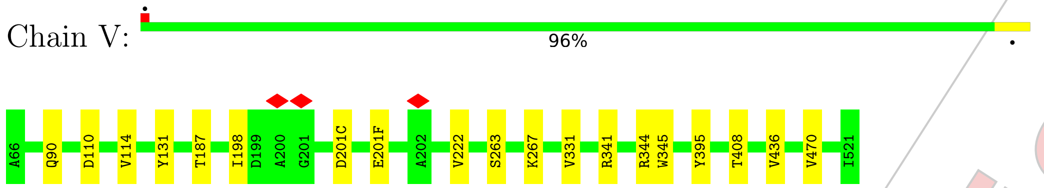

• Molecule 1: Mature major capsid protein

Chain W:

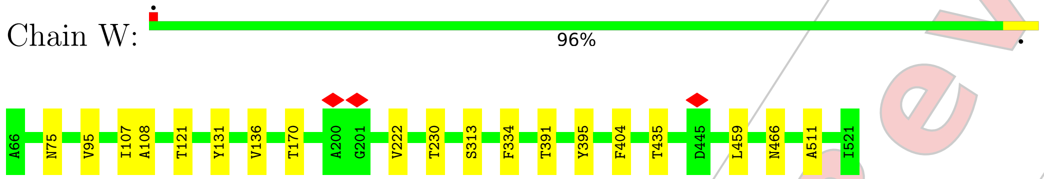

• Molecule 1: Mature major capsid protein

Chain X:

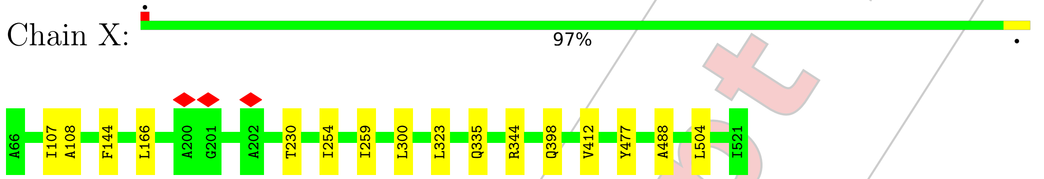

• Molecule 1: Mature major capsid protein

Chain Y:

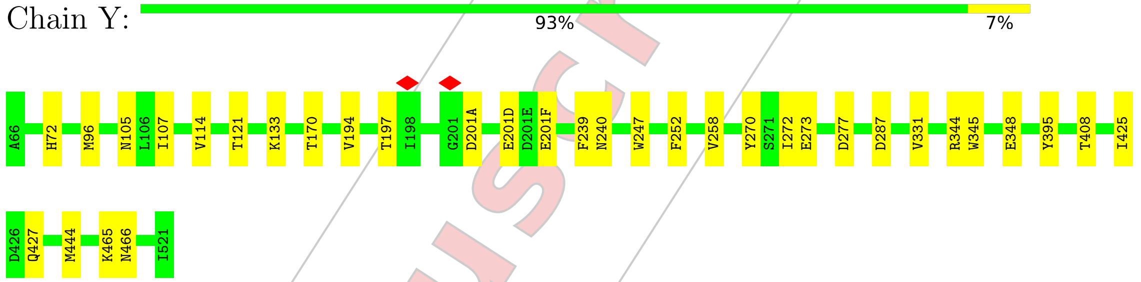

• Molecule 1: Mature major capsid protein

Chain Z:

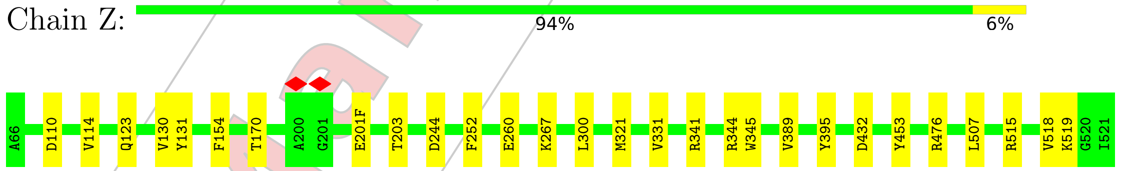

• Molecule 1: Mature major capsid protein

Chain b:

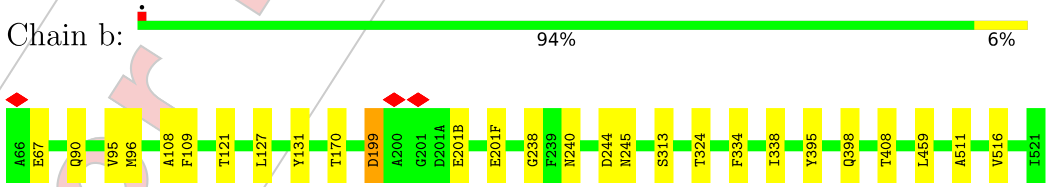

• Molecule 1: Mature major capsid protein

Chain c:

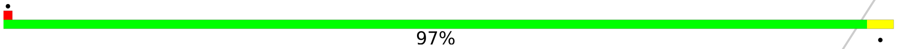

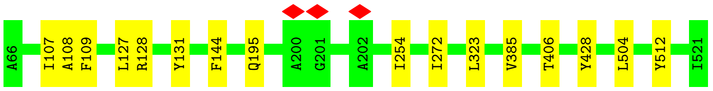

• Molecule 1: Mature major capsid protein

Chain d:

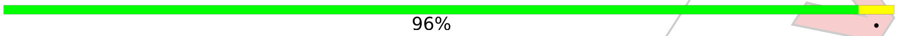

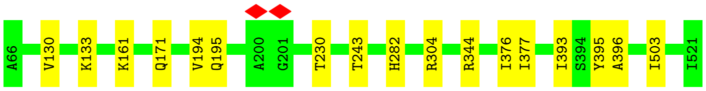

• Molecule 1: Mature major capsid protein

Chain e:

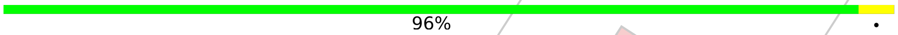

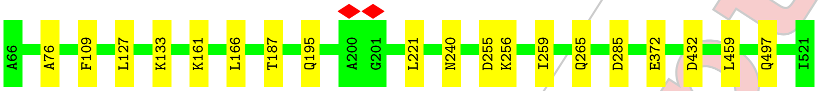

• Molecule 1: Mature major capsid protein

Chain f:

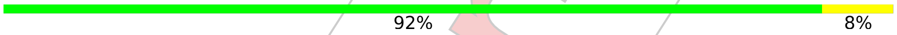

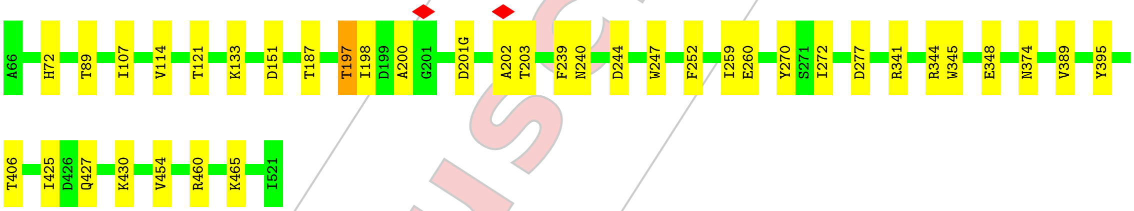

• Molecule 1: Mature major capsid protein

Chain g:

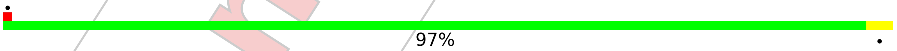

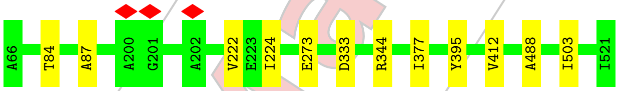

• Molecule 1: Mature major capsid protein

Chain h:

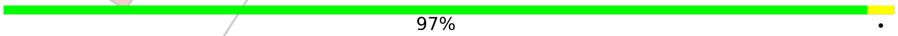

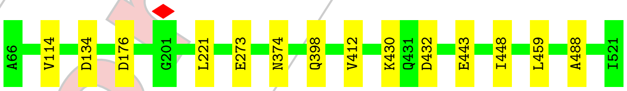

• Molecule 1: Mature major capsid protein

Chain i:

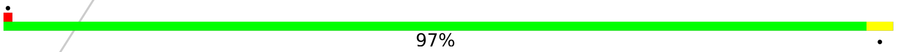

For Manuscript Review

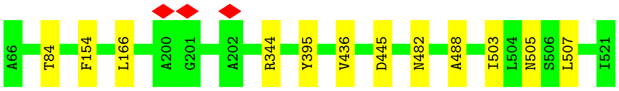

• Molecule 1: Mature major capsid protein

Chain j: 98%

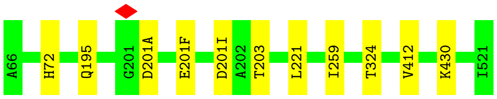

• Molecule 1: Mature major capsid protein

Chain k: 95% 5%

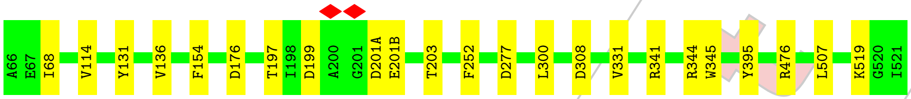

• Molecule 1: Mature major capsid protein

Chain l: 95% 5%

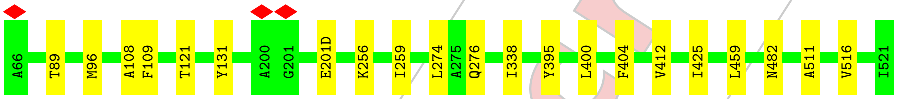

• Molecule 1: Mature major capsid protein

Chain m: 95% 5%

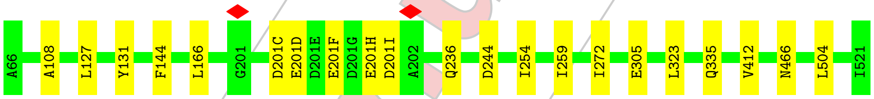

• Molecule 1: Mature major capsid protein

Chain n: 96%

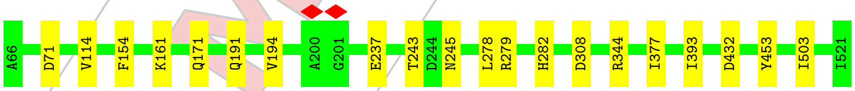

• Molecule 1: Mature major capsid protein

Chain o: 95% 5%

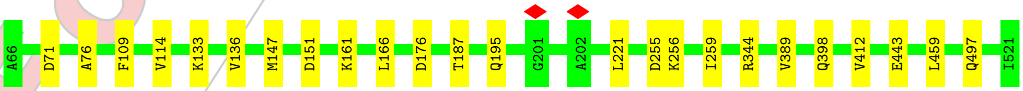

• Molecule 1: Mature major capsid protein

Chain p:

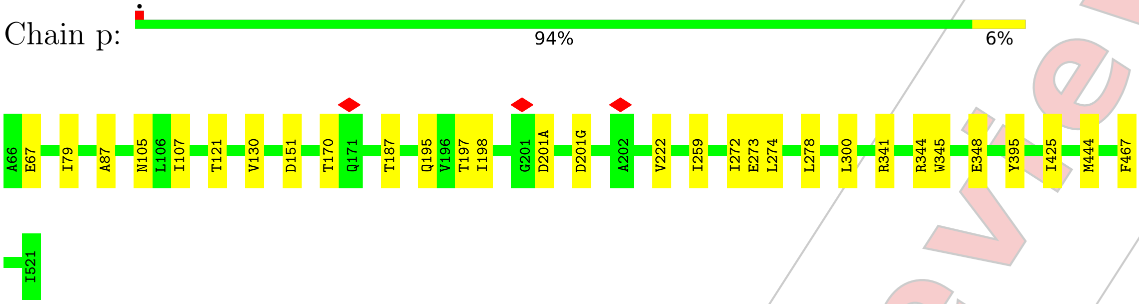

• Molecule 1: Mature major capsid protein

Chain q:

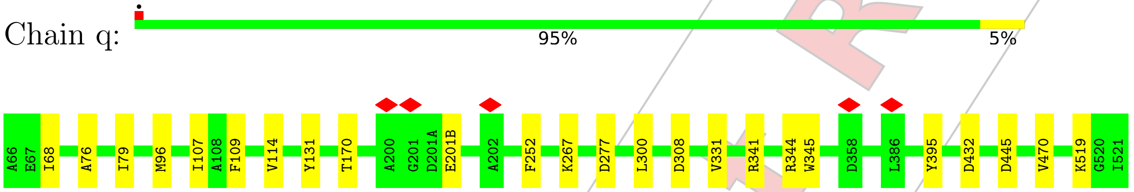

• Molecule 1: Mature major capsid protein

Chain r:

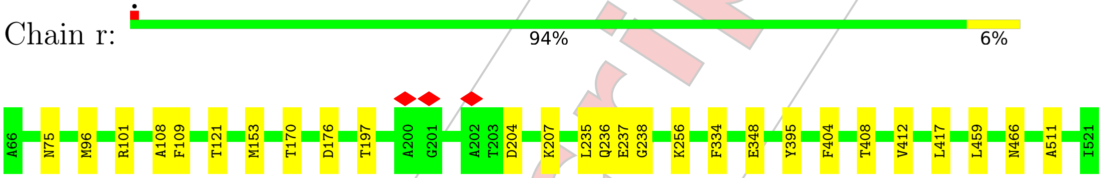

• Molecule 1: Mature major capsid protein

Chain s:

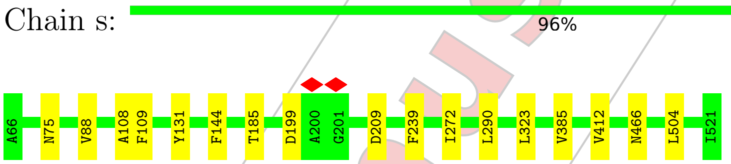

• Molecule 1: Mature major capsid protein

Chain t:

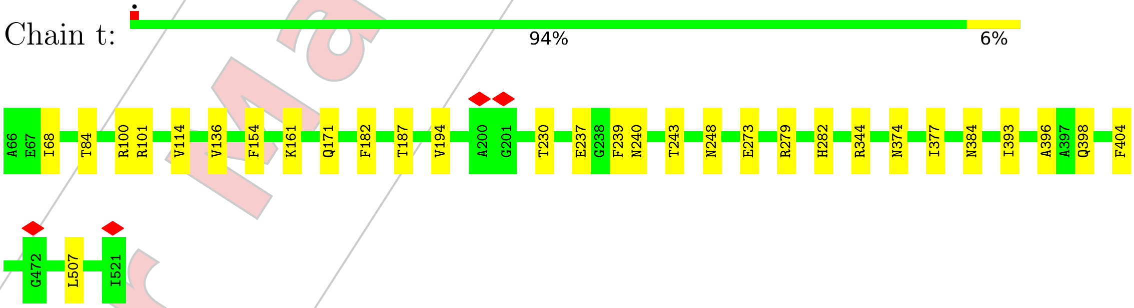

• Molecule 1: Mature major capsid protein

Chain u:

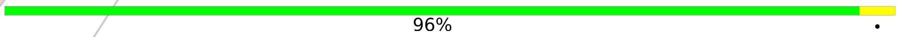

For Manuscript Review

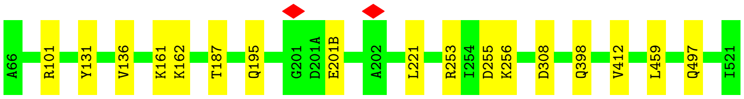

• Molecule 1: Mature major capsid protein

Chain v:

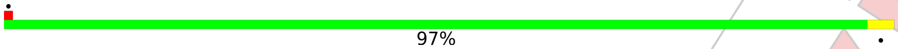

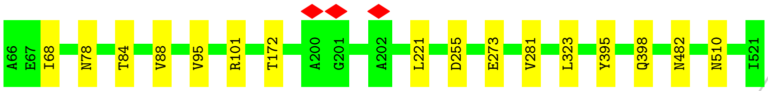

• Molecule 1: Mature major capsid protein

Chain w:

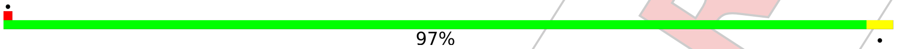

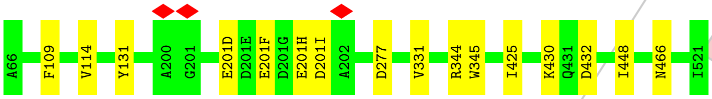

• Molecule 1: Mature major capsid protein

Chain x:

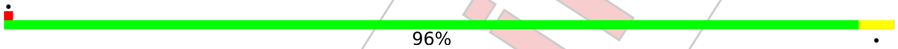

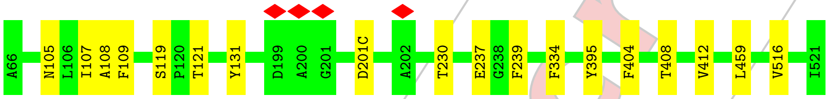

• Molecule 1: Mature major capsid protein

Chain y:

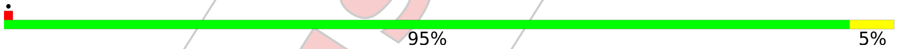

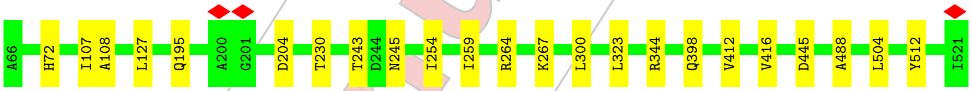

• Molecule 1: Mature major capsid protein

Chain z:

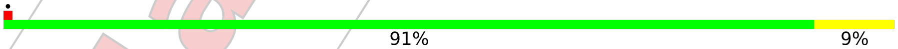

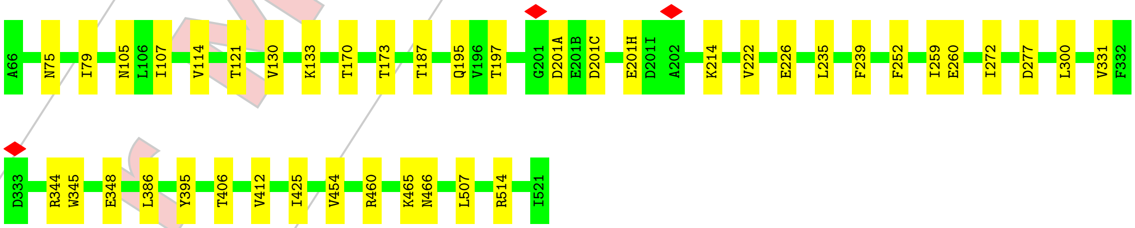

• Molecule 2: Mature capsid vertex protein

Chain A:

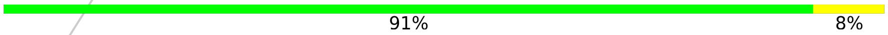

For Manuscript Review

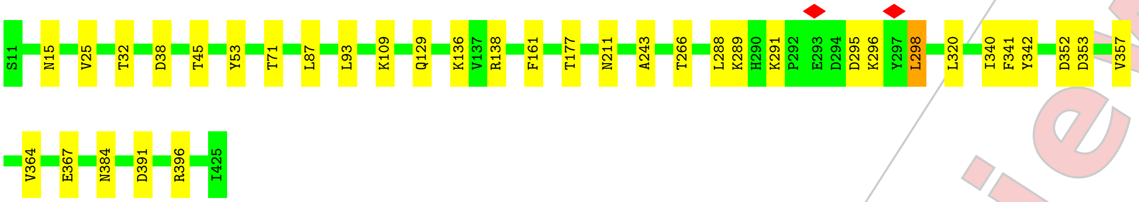

- Molecule 2: Mature capsid vertex protein

Chain B:

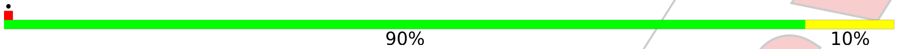

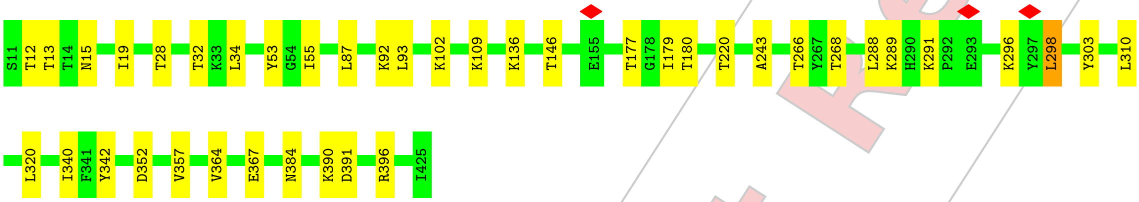

- Molecule 2: Mature capsid vertex protein

Chain C:

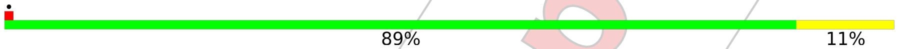

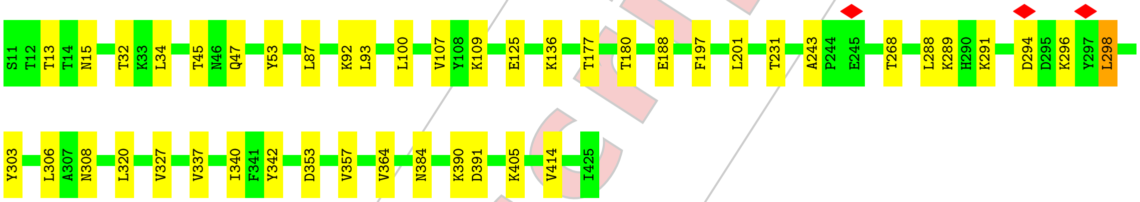

- Molecule 2: Mature capsid vertex protein

Chain D:

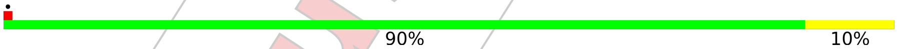

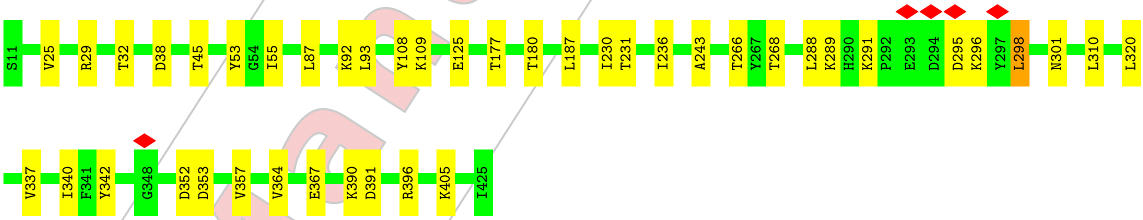

- Molecule 2: Mature capsid vertex protein

Chain a:

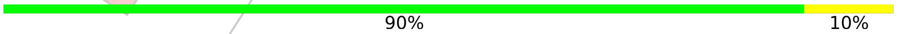

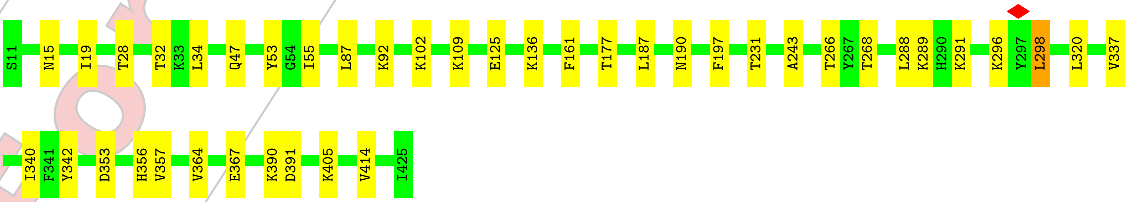

For Manuscript Review

4 Experimental information (i)

Property	Value	Source
EM reconstruction method	SINGLE PARTICLE	Depositor
Imposed symmetry	POINT, D5	Depositor
Number of particles used	70340	Depositor
Resolution determination method	FSC 0.143 CUT-OFF	Depositor
CTF correction method	PHASE FLIPPING AND AMPLITUDE CORRECTION	Depositor
Microscope	FEI TITAN KRIOS	Depositor
Voltage (kV)	300	Depositor
Electron dose $(e^-/\text{Å}^2)$	36	Depositor
Minimum defocus (nm)	800	Depositor
Maximum defocus (nm)	2500	Depositor
Magnification	64000	Depositor
Image detector	GATAN K3 (6k x 4k)	Depositor
Maximum map value	0.084	Depositor
Minimum map value	-0.048	Depositor
Average map value	0.000	Depositor
Map value standard deviation	0.005	Depositor
Recommended contour level	0.014	Depositor
Map size (Å)	1332.0, 1332.0, 1332.0	wwPDB
Map dimensions	720, 720, 720	wwPDB
Map angles (°)	90.0, 90.0, 90.0	wwPDB
Pixel spacing (Å)	1.85, 1.85, 1.85	Depositor

For Manuscript Review

5 Model quality (i)

5.1 Standard geometry (i)

The Z score for a bond length (or angle) is the number of standard deviations the observed value is removed from the expected value. A bond length (or angle) with $|Z| > 5$ is considered an outlier worth inspection. RMSZ is the root-mean-square of all Z scores of the bond lengths (or angles).

Mol	Chain	Bond lengths		Bond angles	
		RMSZ	#$\|Z\|$ >5	RMSZ	#$\|Z\|$ >5
1	0	0.28	0/3569	0.49	0/4834
1	1	0.28	0/3569	0.48	0/4834
1	2	0.27	0/3569	0.49	0/4834
1	3	0.27	0/3569	0.48	0/4834
1	4	0.27	0/3569	0.48	0/4834
1	5	0.27	0/3569	0.48	0/4834
1	6	0.28	0/3569	0.48	0/4834
1	7	0.28	0/3569	0.48	0/4834
1	8	0.28	0/3569	0.49	0/4834
1	9	0.29	0/3569	0.50	0/4834
1	AA	0.28	0/3569	0.49	0/4834
1	AB	0.27	0/3569	0.48	0/4834
1	AC	0.27	0/3569	0.49	0/4834
1	AD	0.30	0/3376	0.49	0/4570
1	AE	0.29	0/3569	0.48	0/4834
1	AF	0.29	0/3569	0.49	0/4834
1	AG	0.29	0/3569	0.49	0/4834
1	AH	0.30	0/3569	0.51	0/4834
1	AI	0.29	0/3569	0.49	0/4834
1	AJ	0.29	0/3569	0.50	0/4834
1	AK	0.29	0/3569	0.49	0/4834
1	AL	0.29	0/3569	0.48	0/4834
1	AM	0.29	0/3569	0.48	0/4834
1	AN	0.29	0/3569	0.48	0/4834
1	AO	0.29	0/3569	0.49	0/4834
1	AP	0.29	0/3554	0.48	0/4813
1	AQ	0.29	0/3569	0.49	0/4834
1	AR	0.28	0/3569	0.48	0/4834
1	AS	0.29	0/3569	0.48	0/4834
1	AT	0.28	0/3569	0.50	0/4834
1	AU	0.29	0/3569	0.48	0/4834
1	AV	0.29	0/3569	0.49	0/4834
1	AW	0.28	0/3569	0.49	0/4834
1	AX	0.28	0/3569	0.48	0/4834

For Manuscript Review

Mol	Chain	Bond lengths		Bond angles	
		RMSZ	#|Z| >5	RMSZ	#|Z| >5
1	AY	0.29	0/3569	0.48	0/4834
1	AZ	0.29	0/3569	0.47	0/4834
1	Aa	0.29	0/3569	0.49	0/4834
1	Ab	0.29	0/3554	0.48	0/4813
1	Ac	0.29	0/3569	0.49	0/4834
1	Ad	0.29	0/3569	0.49	0/4834
1	Ae	0.29	0/3569	0.48	0/4834
1	Af	0.29	0/3569	0.50	0/4834
1	Ag	0.29	0/3569	0.49	0/4834
1	Ah	0.30	0/3569	0.49	0/4834
1	Ai	0.29	0/3569	0.49	0/4834
1	Aj	0.28	0/3569	0.48	0/4834
1	Ak	0.29	0/3569	0.48	0/4834
1	Al	0.29	0/3569	0.48	0/4834
1	Am	0.28	0/3569	0.48	0/4834
1	G	0.27	0/3569	0.49	0/4834
1	H	0.27	0/3569	0.48	0/4834
1	J	0.27	0/3569	0.48	0/4834
1	K	0.29	0/3554	0.48	0/4813
1	L	0.29	0/3569	0.49	0/4834
1	M	0.28	0/3569	0.48	0/4834
1	N	0.28	0/3569	0.48	0/4834
1	O	0.28	0/3569	0.48	0/4834
1	P	0.28	0/3569	0.48	0/4834
1	Q	0.28	0/3569	0.49	0/4834
1	R	0.28	0/3569	0.48	0/4834
1	S	0.27	0/3569	0.48	0/4834
1	T	0.27	0/3569	0.48	0/4834
1	U	0.29	0/3554	0.48	0/4813
1	V	0.29	0/3569	0.49	0/4834
1	W	0.28	0/3569	0.48	0/4834
1	X	0.28	0/3569	0.48	0/4834
1	Y	0.28	0/3569	0.49	0/4834
1	Z	0.28	0/3569	0.49	0/4834
1	b	0.31	0/3569	0.50	0/4834
1	c	0.28	0/3569	0.49	0/4834
1	d	0.28	0/3569	0.48	0/4834
1	e	0.28	0/3569	0.48	0/4834
1	f	0.30	0/3569	0.51	0/4834
1	g	0.28	0/3569	0.48	0/4834
1	h	0.28	0/3569	0.48	0/4834
1	i	0.29	0/3569	0.48	0/4834
1	j	0.29	0/3569	0.48	0/4834

For Manuscript Review

Mol	Chain	Bond lengths		Bond angles	
		RMSZ	#|Z| >5	RMSZ	#|Z| >5
1	k	0.27	0/3569	0.49	0/4834
1	l	0.28	0/3569	0.49	0/4834
1	m	0.27	0/3569	0.49	0/4834
1	n	0.27	0/3569	0.48	0/4834
1	o	0.28	0/3569	0.48	0/4834
1	p	0.27	0/3569	0.49	0/4834
1	q	0.27	0/3569	0.49	0/4834
1	r	0.30	0/3569	0.50	0/4834
1	s	0.27	0/3569	0.49	0/4834
1	t	0.27	0/3569	0.48	0/4834
1	u	0.27	0/3569	0.49	0/4834
1	v	0.28	0/3554	0.48	0/4813
1	w	0.29	0/3569	0.49	0/4834
1	x	0.28	0/3569	0.49	0/4834
1	y	0.28	0/3569	0.48	0/4834
1	z	0.27	0/3569	0.48	0/4834
2	A	0.28	0/3263	0.49	0/4430
2	B	0.28	0/3263	0.48	0/4430
2	C	0.28	0/3263	0.48	0/4430
2	D	0.28	0/3263	0.48	0/4430
2	a	0.28	0/3263	0.49	0/4430
All	All	0.28	0/347964	0.49	0/471343

There are no bond length outliers.

There are no bond angle outliers.

There are no chirality outliers.

There are no planarity outliers.

5.2 Too-close contacts (i)

Due to software issues we are unable to calculate clashes - this section is therefore empty.

5.3 Torsion angles (i)

5.3.1 Protein backbone (i)

In the following table, the Percentiles column shows the percent Ramachandran outliers of the chain as a percentile score with respect to all PDB entries followed by that with respect to all EM entries.

For Manuscript Review

The Analysed column shows the number of residues for which the backbone conformation was analysed, and the total number of residues.

Mol	Chain	Analysed	Favoured	Allowed	Outliers	Percentiles	
1	0	463/465 (100%)	408 (88%)	53 (11%)	2 (0%)	34	71
1	1	463/465 (100%)	408 (88%)	54 (12%)	1 (0%)	47	79
1	2	463/465 (100%)	409 (88%)	50 (11%)	4 (1%)	17	54
1	3	463/465 (100%)	410 (89%)	51 (11%)	2 (0%)	34	71
1	4	463/465 (100%)	405 (88%)	56 (12%)	2 (0%)	34	71
1	5	463/465 (100%)	403 (87%)	59 (13%)	1 (0%)	47	79
1	6	463/465 (100%)	415 (90%)	46 (10%)	2 (0%)	34	71
1	7	463/465 (100%)	416 (90%)	46 (10%)	1 (0%)	47	79
1	8	463/465 (100%)	397 (86%)	63 (14%)	3 (1%)	25	63
1	9	463/465 (100%)	409 (88%)	53 (11%)	1 (0%)	47	79
1	AA	463/465 (100%)	404 (87%)	56 (12%)	3 (1%)	25	63
1	AB	463/465 (100%)	405 (88%)	56 (12%)	2 (0%)	34	71
1	AC	463/465 (100%)	408 (88%)	53 (11%)	2 (0%)	34	71
1	AD	437/465 (94%)	395 (90%)	40 (9%)	2 (0%)	29	67
1	AE	463/465 (100%)	412 (89%)	51 (11%)	0	100	100
1	AF	463/465 (100%)	404 (87%)	57 (12%)	2 (0%)	34	71
1	AG	463/465 (100%)	411 (89%)	49 (11%)	3 (1%)	25	63
1	AH	463/465 (100%)	400 (86%)	59 (13%)	4 (1%)	17	54
1	AI	463/465 (100%)	403 (87%)	59 (13%)	1 (0%)	47	79
1	AJ	463/465 (100%)	404 (87%)	57 (12%)	2 (0%)	34	71
1	AK	463/465 (100%)	401 (87%)	59 (13%)	3 (1%)	25	63
1	AL	463/465 (100%)	399 (86%)	63 (14%)	1 (0%)	47	79
1	AM	463/465 (100%)	404 (87%)	58 (12%)	1 (0%)	47	79
1	AN	463/465 (100%)	405 (88%)	54 (12%)	4 (1%)	17	54
1	AO	463/465 (100%)	409 (88%)	53 (11%)	1 (0%)	47	79
1	AP	463/465 (100%)	425 (92%)	37 (8%)	1 (0%)	47	79
1	AQ	463/465 (100%)	412 (89%)	49 (11%)	2 (0%)	34	71
1	AR	463/465 (100%)	410 (89%)	50 (11%)	3 (1%)	25	63
1	AS	463/465 (100%)	415 (90%)	45 (10%)	3 (1%)	25	63
1	AT	463/465 (100%)	396 (86%)	63 (14%)	4 (1%)	17	54

Continued on next page...

For Manuscript Review

Continued from previous page...

Mol	Chain	Analysed	Favoured	Allowed	Outliers	Percentiles	
1	AU	463/465 (100%)	414 (89%)	48 (10%)	1 (0%)	47	79
1	AV	463/465 (100%)	405 (88%)	55 (12%)	3 (1%)	25	63
1	AW	463/465 (100%)	406 (88%)	54 (12%)	3 (1%)	25	63
1	AX	463/465 (100%)	406 (88%)	56 (12%)	1 (0%)	47	79
1	AY	463/465 (100%)	405 (88%)	57 (12%)	1 (0%)	47	79
1	AZ	463/465 (100%)	413 (89%)	48 (10%)	2 (0%)	34	71
1	Aa	463/465 (100%)	414 (89%)	48 (10%)	1 (0%)	47	79
1	Ab	463/465 (100%)	420 (91%)	40 (9%)	3 (1%)	25	63
1	Ac	463/465 (100%)	413 (89%)	50 (11%)	0	100	100
1	Ad	463/465 (100%)	413 (89%)	48 (10%)	2 (0%)	34	71
1	Ae	463/465 (100%)	410 (89%)	50 (11%)	3 (1%)	25	63
1	Af	463/465 (100%)	402 (87%)	56 (12%)	5 (1%)	14	51
1	Ag	463/465 (100%)	403 (87%)	60 (13%)	0	100	100
1	Ah	463/465 (100%)	407 (88%)	54 (12%)	2 (0%)	34	71
1	Ai	463/465 (100%)	400 (86%)	60 (13%)	3 (1%)	25	63
1	Aj	463/465 (100%)	406 (88%)	56 (12%)	1 (0%)	47	79
1	Ak	463/465 (100%)	404 (87%)	58 (12%)	1 (0%)	47	79
1	Al	463/465 (100%)	409 (88%)	53 (11%)	1 (0%)	47	79
1	Am	463/465 (100%)	411 (89%)	51 (11%)	1 (0%)	47	79
1	G	463/465 (100%)	407 (88%)	52 (11%)	4 (1%)	17	54
1	H	463/465 (100%)	406 (88%)	54 (12%)	3 (1%)	25	63
1	J	463/465 (100%)	403 (87%)	57 (12%)	3 (1%)	25	63
1	K	463/465 (100%)	422 (91%)	39 (8%)	2 (0%)	34	71
1	L	463/465 (100%)	408 (88%)	55 (12%)	0	100	100
1	M	463/465 (100%)	409 (88%)	52 (11%)	2 (0%)	34	71
1	N	463/465 (100%)	414 (89%)	46 (10%)	3 (1%)	25	63
1	O	463/465 (100%)	398 (86%)	63 (14%)	2 (0%)	34	71
1	P	463/465 (100%)	405 (88%)	56 (12%)	2 (0%)	34	71
1	Q	463/465 (100%)	404 (87%)	53 (11%)	6 (1%)	12	48
1	R	463/465 (100%)	408 (88%)	52 (11%)	3 (1%)	25	63
1	S	463/465 (100%)	398 (86%)	64 (14%)	1 (0%)	47	79

Continued on next page...

For Manuscript Review

Continued from previous page...

Mol	Chain	Analysed	Favoured	Allowed	Outliers	Percentiles	
1	T	463/465 (100%)	412 (89%)	50 (11%)	1 (0%)	47	79
1	U	463/465 (100%)	426 (92%)	34 (7%)	3 (1%)	25	63
1	V	463/465 (100%)	413 (89%)	50 (11%)	0	100	100
1	W	463/465 (100%)	413 (89%)	48 (10%)	2 (0%)	34	71
1	X	463/465 (100%)	409 (88%)	51 (11%)	3 (1%)	25	63
1	Y	463/465 (100%)	405 (88%)	56 (12%)	2 (0%)	34	71
1	Z	463/465 (100%)	414 (89%)	49 (11%)	0	100	100
1	b	463/465 (100%)	408 (88%)	48 (10%)	7 (2%)	10	45
1	c	463/465 (100%)	410 (89%)	51 (11%)	2 (0%)	34	71
1	d	463/465 (100%)	405 (88%)	56 (12%)	2 (0%)	34	71
1	e	463/465 (100%)	403 (87%)	59 (13%)	1 (0%)	47	79
1	f	463/465 (100%)	399 (86%)	57 (12%)	7 (2%)	10	45
1	g	463/465 (100%)	414 (89%)	47 (10%)	2 (0%)	34	71
1	h	463/465 (100%)	411 (89%)	51 (11%)	1 (0%)	47	79
1	i	463/465 (100%)	405 (88%)	57 (12%)	1 (0%)	47	79
1	j	463/465 (100%)	404 (87%)	58 (12%)	1 (0%)	47	79
1	k	463/465 (100%)	411 (89%)	52 (11%)	0	100	100
1	l	463/465 (100%)	401 (87%)	60 (13%)	2 (0%)	34	71
1	m	463/465 (100%)	398 (86%)	63 (14%)	2 (0%)	34	71
1	n	463/465 (100%)	401 (87%)	61 (13%)	1 (0%)	47	79
1	o	463/465 (100%)	408 (88%)	54 (12%)	1 (0%)	47	79
1	p	463/465 (100%)	404 (87%)	56 (12%)	3 (1%)	25	63
1	q	463/465 (100%)	411 (89%)	51 (11%)	1 (0%)	47	79
1	r	463/465 (100%)	397 (86%)	61 (13%)	5 (1%)	14	51
1	s	463/465 (100%)	405 (88%)	54 (12%)	4 (1%)	17	54
1	t	463/465 (100%)	405 (88%)	55 (12%)	3 (1%)	25	63
1	u	463/465 (100%)	391 (84%)	72 (16%)	0	100	100
1	v	463/465 (100%)	421 (91%)	40 (9%)	2 (0%)	34	71
1	w	463/465 (100%)	420 (91%)	43 (9%)	0	100	100
1	x	463/465 (100%)	415 (90%)	47 (10%)	1 (0%)	47	79
1	y	463/465 (100%)	412 (89%)	48 (10%)	3 (1%)	25	63

Continued on next page...

For Manuscript Review

Continued from previous page...

Mol	Chain	Analysed	Favoured	Allowed	Outliers	Percentiles	
1	z	463/465 (100%)	410 (89%)	51 (11%)	2 (0%)	34	71
2	A	413/415 (100%)	364 (88%)	42 (10%)	7 (2%)	9	43
2	B	413/415 (100%)	361 (87%)	46 (11%)	6 (2%)	10	45
2	C	413/415 (100%)	362 (88%)	45 (11%)	6 (2%)	10	45
2	D	413/415 (100%)	362 (88%)	44 (11%)	7 (2%)	9	43
2	a	413/415 (100%)	365 (88%)	41 (10%)	7 (2%)	9	43
All	All	45098/45320 (100%)	39720 (88%)	5152 (11%)	226 (0%)	32	67

All (226) Ramachandran outliers are listed below:

Mol	Chain	Res	Type
1	2	88	VAL
1	2	108	ALA
1	2	201(I)	ASP
1	3	108	ALA
1	6	488	ALA
1	7	488	ALA
1	8	272	ILE
1	9	201(C)	ASP
2	A	298	LEU
1	AA	108	ALA
1	AG	108	ALA
1	AG	488	ALA
1	AH	226	GLU
1	AH	272	ILE
1	AK	108	ALA
1	AK	199	ASP
1	AN	488	ALA
1	AO	201(B)	GLU
1	AS	108	ALA
1	AS	488	ALA
1	AT	272	ILE
1	AW	108	ALA
1	AW	199	ASP
1	AW	504	LEU
1	AZ	488	ALA
1	Aa	488	ALA
1	Ab	201(H)	GLU
1	Ae	108	ALA
1	Ae	488	ALA

Continued on next page...

For Manuscript Review

Continued from previous page...

Mol	Chain	Res	Type
1	Af	226	GLU
1	Af	272	ILE
1	Ai	108	ALA
1	Ai	199	ASP
1	Ai	504	LEU
1	Al	488	ALA
1	Am	488	ALA
2	B	298	LEU
2	C	298	LEU
2	D	298	LEU
1	G	237	GLU
1	G	465	LYS
1	H	108	ALA
1	J	199	ASP
1	N	108	ALA
1	N	488	ALA
1	O	272	ILE
1	Q	201(G)	ASP
1	Q	201(I)	ASP
1	R	199	ASP
1	X	488	ALA
1	Y	272	ILE
2	a	298	LEU
1	b	201(B)	GLU
1	c	108	ALA
1	c	504	LEU
1	f	198	ILE
1	f	272	ILE
1	g	488	ALA
1	h	488	ALA
1	i	488	ALA
1	m	108	ALA
1	m	504	LEU
1	p	272	ILE
1	s	88	VAL
1	s	108	ALA
1	s	199	ASP
1	s	504	LEU
1	y	108	ALA
1	y	488	ALA
1	z	272	ILE
1	1	87	ALA

Continued on next page...

For Manuscript Review

Continued from previous page...

Mol	Chain	Res	Type
1	2	511	ALA
1	3	504	LEU
1	6	87	ALA
1	AA	199	ASP
1	AA	504	LEU
1	AC	198	ILE
1	AF	108	ALA
1	AG	504	LEU
1	AI	87	ALA
1	AJ	108	ALA
1	AJ	511	ALA
1	AK	504	LEU
1	AN	87	ALA
1	AN	231	SER
1	AP	281	VAL
1	AQ	201(I)	ASP
1	AR	201(F)	GLU
1	AR	511	ALA
1	AS	504	LEU
1	AT	201(G)	ASP
1	AT	261	ALA
1	AU	87	ALA
1	AV	108	ALA
1	AV	201(A)	ASP
1	AV	511	ALA
1	AZ	87	ALA
1	Ab	281	VAL
1	Ad	108	ALA
1	Ad	511	ALA
1	Ae	504	LEU
1	Af	202	ALA
1	Af	261	ALA
1	Ah	108	ALA
1	Ah	511	ALA
1	G	108	ALA
1	G	511	ALA
1	H	504	LEU
1	J	201(B)	GLU
1	K	281	VAL
1	M	108	ALA
1	N	504	LEU
1	Q	108	ALA

Continued on next page...

For Manuscript Review

Continued from previous page...

Mol	Chain	Res	Type
1	Q	511	ALA
1	R	108	ALA
1	R	504	LEU
1	U	281	VAL
1	W	108	ALA
1	W	511	ALA
1	X	108	ALA
1	X	504	LEU
1	b	108	ALA
1	b	199	ASP
1	b	238	GLY
1	b	511	ALA
1	e	76	ALA
1	f	203	THR
1	j	201(A)	ASP
1	l	108	ALA
1	l	511	ALA
1	p	87	ALA
1	r	108	ALA
1	r	235	LEU
1	r	238	GLY
1	v	281	VAL
1	x	108	ALA
1	y	504	LEU
1	0	108	ALA
1	8	121	THR
1	8	261	ALA
1	AC	76	ALA
1	AD	200	ALA
1	AF	511	ALA
1	AH	261	ALA
1	AM	76	ALA
1	AN	233	ALA
1	AQ	201(C)	ASP
1	AR	108	ALA
1	AY	76	ALA
1	Ak	76	ALA
1	M	511	ALA
1	P	87	ALA
1	Q	201(H)	GLU
1	T	76	ALA
2	a	342	TYR

Continued on next page...

For Manuscript Review

Continued from previous page...

Mol	Chain	Res	Type
1	b	245	ASN
1	f	121	THR
1	g	87	ALA
1	o	76	ALA
1	r	511	ALA
1	z	121	THR
1	5	76	ALA
2	A	353	ASP
1	AD	201(B)	GLU
1	AH	121	THR
1	Af	121	THR
2	D	342	TYR
1	J	76	ALA
1	O	121	THR
1	Y	121	THR
1	f	197	THR
1	f	200	ALA
1	r	237	GLU
1	4	76	ALA
1	4	393	ILE
2	A	340	ILE
2	A	342	TYR
1	AB	393	ILE
1	AL	393	ILE
1	AX	393	ILE
1	Aj	393	ILE
2	B	243	ALA
2	B	340	ILE
2	B	342	TYR
2	B	364	VAL
2	C	243	ALA
2	C	340	ILE
2	C	364	VAL
2	D	243	ALA
2	D	340	ILE
2	D	353	ASP
1	H	201(G)	ASP
1	Q	201(F)	GLU
1	S	393	ILE
1	U	201(B)	GLU
2	a	243	ALA
2	a	340	ILE

Continued on next page...

For Manuscript Review

Continued from previous page...

Mol	Chain	Res	Type
2	a	353	ASP
2	a	364	VAL
1	b	244	ASP
1	d	393	ILE
1	d	396	ALA
1	f	202	ALA
1	n	393	ILE
1	t	239	PHE
1	t	393	ILE
1	t	396	ALA
2	A	364	VAL
1	AB	396	ALA
1	AT	121	THR
2	C	342	TYR
2	D	364	VAL
1	P	121	THR
1	p	121	THR
1	q	76	ALA
1	K	88	VAL
1	U	88	VAL
2	A	243	ALA
2	C	357	VAL
2	A	357	VAL
1	Ab	88	VAL
2	D	357	VAL
2	a	357	VAL
1	v	88	VAL
1	0	82	GLY
2	B	357	VAL

5.3.2 Protein sidechains (i)

In the following table, the Percentiles column shows the percent sidechain outliers of the chain as a percentile score with respect to all PDB entries followed by that with respect to all EM entries.

The Analysed column shows the number of residues for which the sidechain conformation was analysed, and the total number of residues.

Mol	Chain	Analysed	Rotameric	Outliers	Percentiles	
1	0	354/354 (100%)	330 (93%)	24 (7%)	16	45
1	1	354/354 (100%)	336 (95%)	18 (5%)	24	53

Continued on next page...

For Manuscript Review

Continued from previous page...

Mol	Chain	Analysed	Rotameric	Outliers	Percentiles	
1	2	354/354 (100%)	332 (94%)	22 (6%)	18	48
1	3	354/354 (100%)	337 (95%)	17 (5%)	25	54
1	4	354/354 (100%)	326 (92%)	28 (8%)	12	41
1	5	354/354 (100%)	333 (94%)	21 (6%)	19	49
1	6	354/354 (100%)	343 (97%)	11 (3%)	40	64
1	7	354/354 (100%)	341 (96%)	13 (4%)	34	60
1	8	354/354 (100%)	320 (90%)	34 (10%)	8	32
1	9	354/354 (100%)	336 (95%)	18 (5%)	24	53
1	AA	354/354 (100%)	340 (96%)	14 (4%)	31	58
1	AB	354/354 (100%)	326 (92%)	28 (8%)	12	41
1	AC	354/354 (100%)	326 (92%)	28 (8%)	12	41
1	AD	336/354 (95%)	315 (94%)	21 (6%)	18	47
1	AE	354/354 (100%)	332 (94%)	22 (6%)	18	48
1	AF	354/354 (100%)	321 (91%)	33 (9%)	9	34
1	AG	354/354 (100%)	338 (96%)	16 (4%)	27	56
1	AH	354/354 (100%)	319 (90%)	35 (10%)	8	31
1	AI	354/354 (100%)	322 (91%)	32 (9%)	9	36
1	AJ	354/354 (100%)	330 (93%)	24 (7%)	16	45
1	AK	354/354 (100%)	336 (95%)	18 (5%)	24	53
1	AL	354/354 (100%)	328 (93%)	26 (7%)	14	43
1	AM	354/354 (100%)	332 (94%)	22 (6%)	18	48
1	AN	354/354 (100%)	342 (97%)	12 (3%)	37	62
1	AO	354/354 (100%)	339 (96%)	15 (4%)	30	57
1	AP	352/354 (99%)	336 (96%)	16 (4%)	27	56
1	AQ	354/354 (100%)	329 (93%)	25 (7%)	14	44
1	AR	354/354 (100%)	333 (94%)	21 (6%)	19	49
1	AS	354/354 (100%)	339 (96%)	15 (4%)	30	57
1	AT	354/354 (100%)	322 (91%)	32 (9%)	9	36
1	AU	354/354 (100%)	330 (93%)	24 (7%)	16	45
1	AV	354/354 (100%)	326 (92%)	28 (8%)	12	41
1	AW	354/354 (100%)	340 (96%)	14 (4%)	31	58

Continued on next page...

For Manuscript Review

Continued from previous page...

Mol	Chain	Analysed	Rotameric	Outliers	Percentiles	
1	AX	354/354 (100%)	328 (93%)	26 (7%)	14	43
1	AY	354/354 (100%)	330 (93%)	24 (7%)	16	45
1	AZ	354/354 (100%)	344 (97%)	10 (3%)	43	66
1	Aa	354/354 (100%)	341 (96%)	13 (4%)	34	60
1	Ab	352/354 (99%)	331 (94%)	21 (6%)	19	49
1	Ac	354/354 (100%)	334 (94%)	20 (6%)	21	51
1	Ad	354/354 (100%)	337 (95%)	17 (5%)	25	54
1	Ae	354/354 (100%)	338 (96%)	16 (4%)	27	56
1	Af	354/354 (100%)	317 (90%)	37 (10%)	7	29
1	Ag	354/354 (100%)	332 (94%)	22 (6%)	18	48
1	Ah	354/354 (100%)	328 (93%)	26 (7%)	14	43
1	Ai	354/354 (100%)	333 (94%)	21 (6%)	19	49
1	Aj	354/354 (100%)	332 (94%)	22 (6%)	18	48
1	Ak	354/354 (100%)	338 (96%)	16 (4%)	27	56
1	Al	354/354 (100%)	341 (96%)	13 (4%)	34	60
1	Am	354/354 (100%)	344 (97%)	10 (3%)	43	66
1	G	354/354 (100%)	329 (93%)	25 (7%)	14	44
1	H	354/354 (100%)	332 (94%)	22 (6%)	18	48
1	J	354/354 (100%)	335 (95%)	19 (5%)	22	52
1	K	352/354 (99%)	333 (95%)	19 (5%)	22	52
1	L	354/354 (100%)	340 (96%)	14 (4%)	31	58
1	M	354/354 (100%)	339 (96%)	15 (4%)	30	57
1	N	354/354 (100%)	341 (96%)	13 (4%)	34	60
1	O	354/354 (100%)	315 (89%)	39 (11%)	6	28
1	P	354/354 (100%)	328 (93%)	26 (7%)	14	43
1	Q	354/354 (100%)	332 (94%)	22 (6%)	18	48
1	R	354/354 (100%)	334 (94%)	20 (6%)	21	51
1	S	354/354 (100%)	326 (92%)	28 (8%)	12	41
1	T	354/354 (100%)	332 (94%)	22 (6%)	18	48
1	U	352/354 (99%)	334 (95%)	18 (5%)	24	53
1	V	354/354 (100%)	335 (95%)	19 (5%)	22	52

Continued on next page...

For Manuscript Review

Continued from previous page...

Mol	Chain	Analysed	Rotameric	Outliers	Percentiles	
1	W	354/354 (100%)	337 (95%)	17 (5%)	25	54
1	X	354/354 (100%)	341 (96%)	13 (4%)	34	60
1	Y	354/354 (100%)	322 (91%)	32 (9%)	9	36
1	Z	354/354 (100%)	326 (92%)	28 (8%)	12	41
1	b	354/354 (100%)	333 (94%)	21 (6%)	19	49
1	c	354/354 (100%)	340 (96%)	14 (4%)	31	58
1	d	354/354 (100%)	339 (96%)	15 (4%)	30	57
1	e	354/354 (100%)	336 (95%)	18 (5%)	24	53
1	f	354/354 (100%)	322 (91%)	32 (9%)	9	36
1	g	354/354 (100%)	344 (97%)	10 (3%)	43	66
1	h	354/354 (100%)	341 (96%)	13 (4%)	34	60
1	i	354/354 (100%)	343 (97%)	11 (3%)	40	64
1	j	354/354 (100%)	344 (97%)	10 (3%)	43	66
1	k	354/354 (100%)	331 (94%)	23 (6%)	17	46
1	l	354/354 (100%)	335 (95%)	19 (5%)	22	52
1	m	354/354 (100%)	335 (95%)	19 (5%)	22	52
1	n	354/354 (100%)	335 (95%)	19 (5%)	22	52
1	o	354/354 (100%)	331 (94%)	23 (6%)	17	46
1	p	354/354 (100%)	327 (92%)	27 (8%)	13	42
1	q	354/354 (100%)	331 (94%)	23 (6%)	17	46
1	r	354/354 (100%)	332 (94%)	22 (6%)	18	48
1	s	354/354 (100%)	341 (96%)	13 (4%)	34	60
1	t	354/354 (100%)	327 (92%)	27 (8%)	13	42
1	u	354/354 (100%)	337 (95%)	17 (5%)	25	54
1	v	352/354 (99%)	338 (96%)	14 (4%)	31	58
1	w	354/354 (100%)	338 (96%)	16 (4%)	27	56
1	x	354/354 (100%)	337 (95%)	17 (5%)	25	54
1	y	354/354 (100%)	334 (94%)	20 (6%)	21	51
1	z	354/354 (100%)	314 (89%)	40 (11%)	6	27
2	A	350/350 (100%)	320 (91%)	30 (9%)	10	38
2	B	350/350 (100%)	314 (90%)	36 (10%)	7	30

Continued on next page...

For Manuscript Review

Continued from previous page...

Mol	Chain	Analysed	Rotameric	Outliers	Percentiles	
2	C	350/350 (100%)	310 (89%)	40 (11%)	5	26
2	D	350/350 (100%)	313 (89%)	37 (11%)	6	29
2	a	350/350 (100%)	315 (90%)	35 (10%)	7	30
All	All	34644/34672 (100%)	32531 (94%)	2113 (6%)	22	48

All (2113) residues with a non-rotameric sidechain are listed below:

Mol	Chain	Res	Type
1	0	77	THR
1	0	90	GLN
1	0	121	THR
1	0	131	TYR
1	0	170	THR
1	0	198	ILE
1	0	201(B)	GLU
1	0	201(H)	GLU
1	0	222	VAL
1	0	256	LYS
1	0	279	ARG
1	0	324	THR
1	0	331	VAL
1	0	334	PHE
1	0	375	PHE
1	0	395	TYR
1	0	398	GLN
1	0	400	LEU
1	0	404	PHE
1	0	408	THR
1	0	412	VAL
1	0	466	ASN
1	0	510	ASN
1	0	516	VAL
1	1	68	ILE
1	1	109	PHE
1	1	114	VAL
1	1	154	PHE
1	1	162	LYS
1	1	170	THR
1	1	222	VAL
1	1	260	GLU
1	1	267	LYS

Continued on next page...

For Manuscript Review

Continued from previous page...

Mol	Chain	Res	Type
1	1	277	ASP
1	1	299	MET
1	1	331	VAL
1	1	341	ARG
1	1	344	ARG
1	1	345	TRP
1	1	395	TYR
1	1	476	ARG
1	1	519	LYS
1	2	83	GLN
1	2	99	VAL
1	2	109	PHE
1	2	121	THR
1	2	136	VAL
1	2	197	THR
1	2	201(C)	ASP
1	2	201(E)	ASP
1	2	201(F)	GLU
1	2	203	THR
1	2	204	ASP
1	2	256	LYS
1	2	338	ILE
1	2	344	ARG
1	2	348	GLU
1	2	379	SER
1	2	395	TYR
1	2	404	PHE
1	2	412	VAL
1	2	448	ILE
1	2	467	PHE
1	2	516	VAL
1	3	75	ASN
1	3	84	THR
1	3	109	PHE
1	3	131	TYR
1	3	144	PHE
1	3	176	ASP
1	3	195	GLN
1	3	201(B)	GLU
1	3	254	ILE
1	3	259	ILE
1	3	272	ILE

Continued on next page...

For Manuscript Review

Continued from previous page...

Mol	Chain	Res	Type
1	3	323	LEU
1	3	345	TRP
1	3	398	GLN
1	3	428	TYR
1	3	505	ASN
1	3	518	VAL
1	4	68	ILE
1	4	78	ASN
1	4	84	THR
1	4	96	MET
1	4	114	VAL
1	4	127	LEU
1	4	154	PHE
1	4	161	LYS
1	4	171	GLN
1	4	191	GLN
1	4	201(D)	GLU
1	4	201(I)	ASP
1	4	204	ASP
1	4	217	GLU
1	4	230	THR
1	4	234	GLU
1	4	243	THR
1	4	273	GLU
1	4	279	ARG
1	4	282	HIS
1	4	289	GLU
1	4	303	ASN
1	4	344	ARG
1	4	395	TYR
1	4	398	GLN
1	4	404	PHE
1	4	434	PHE
1	4	503	ILE
1	5	68	ILE
1	5	101	ARG
1	5	106	LEU
1	5	109	PHE
1	5	133	LYS
1	5	166	LEU
1	5	195	GLN
1	5	199	ASP

Continued on next page...

For Manuscript Review

Continued from previous page...

Mol	Chain	Res	Type
1	5	201(D)	GLU
1	5	201(H)	GLU
1	5	201(I)	ASP
1	5	221	LEU
1	5	255	ASP
1	5	256	LYS
1	5	259	ILE
1	5	285	ASP
1	5	344	ARG
1	5	384	ASN
1	5	412	VAL
1	5	459	LEU
1	5	497	GLN
1	6	96	MET
1	6	166	LEU
1	6	188	VAL
1	6	204	ASP
1	6	224	ILE
1	6	304	ARG
1	6	328	LYS
1	6	344	ARG
1	6	377	ILE
1	6	503	ILE
1	6	507	LEU
1	7	72	HIS
1	7	75	ASN
1	7	109	PHE
1	7	166	LEU
1	7	221	LEU
1	7	259	ILE
1	7	344	ARG
1	7	358	ASP
1	7	368	THR
1	7	398	GLN
1	7	430	LYS
1	7	432	ASP
1	7	459	LEU
1	8	84	THR
1	8	107	ILE
1	8	114	VAL
1	8	133	LYS
1	8	153	MET

Continued on next page...

For Manuscript Review

Continued from previous page...

Mol	Chain	Res	Type
1	8	161	LYS
1	8	162	LYS
1	8	170	THR
1	8	181	PHE
1	8	197	THR
1	8	201(F)	GLU
1	8	217	GLU
1	8	222	VAL
1	8	231	SER
1	8	239	PHE
1	8	240	ASN
1	8	252	PHE
1	8	260	GLU
1	8	270	TYR
1	8	273	GLU
1	8	277	ASP
1	8	284	MET
1	8	287	ASP
1	8	300	LEU
1	8	331	VAL
1	8	344	ARG
1	8	345	TRP
1	8	348	GLU
1	8	386	LEU
1	8	395	TYR
1	8	425	ILE
1	8	460	ARG
1	8	468	GLN
1	8	507	LEU
1	9	105	ASN
1	9	110	ASP
1	9	114	VAL
1	9	154	PHE
1	9	199	ASP
1	9	201(A)	ASP
1	9	201(D)	GLU
1	9	252	PHE
1	9	267	LYS
1	9	277	ASP
1	9	331	VAL
1	9	341	ARG
1	9	344	ARG

Continued on next page...

For Manuscript Review

Continued from previous page...

Mol	Chain	Res	Type
1	9	345	TRP
1	9	395	TYR
1	9	412	VAL
1	9	515	ARG
1	9	519	LYS
2	A	15	ASN
2	A	25	VAL
2	A	32	THR
2	A	38	ASP
2	A	45	THR
2	A	53	TYR
2	A	71	THR
2	A	87	LEU
2	A	93	LEU
2	A	109	LYS
2	A	129	GLN
2	A	136	LYS
2	A	138	ARG
2	A	161	PHE
2	A	177	THR
2	A	211	ASN
2	A	266	THR
2	A	288	LEU
2	A	289	LYS
2	A	291	LYS
2	A	295	ASP
2	A	296	LYS
2	A	298	LEU
2	A	320	LEU
2	A	341	PHE
2	A	352	ASP
2	A	367	GLU
2	A	384	ASN
2	A	391	ASP
2	A	396	ARG
1	AA	84	THR
1	AA	109	PHE
1	AA	131	TYR
1	AA	144	PHE
1	AA	195	GLN
1	AA	235	LEU
1	AA	254	ILE

Continued on next page...

For Manuscript Review

Continued from previous page...

Mol	Chain	Res	Type
1	AA	323	LEU
1	AA	334	PHE
1	AA	385	VAL
1	AA	416	VAL
1	AA	457	THR
1	AA	460	ARG
1	AA	463	ASP
1	AB	71	ASP
1	AB	78	ASN
1	AB	96	MET
1	AB	99	VAL
1	AB	114	VAL
1	AB	154	PHE
1	AB	161	LYS
1	AB	194	VAL
1	AB	201(C)	ASP
1	AB	201(F)	GLU
1	AB	201(G)	ASP
1	AB	217	GLU
1	AB	230	THR
1	AB	235	LEU
1	AB	245	ASN
1	AB	247	TRP
1	AB	252	PHE
1	AB	273	GLU
1	AB	277	ASP
1	AB	308	ASP
1	AB	341	ARG
1	AB	344	ARG
1	AB	374	ASN
1	AB	377	ILE
1	AB	398	GLN
1	AB	412	VAL
1	AB	481	ILE
1	AB	507	LEU
1	AC	101	ARG
1	AC	106	LEU
1	AC	109	PHE
1	AC	133	LYS
1	AC	134	ASP
1	AC	176	ASP
1	AC	187	THR

Continued on next page...

For Manuscript Review

Continued from previous page...

Mol	Chain	Res	Type
1	AC	191	GLN
1	AC	197	THR
1	AC	201(B)	GLU
1	AC	201(E)	ASP
1	AC	201(H)	GLU
1	AC	204	ASP
1	AC	221	LEU
1	AC	256	LYS
1	AC	259	ILE
1	AC	274	LEU
1	AC	278	LEU
1	AC	279	ARG
1	AC	313	SER
1	AC	374	ASN
1	AC	398	GLN
1	AC	406	THR
1	AC	412	VAL
1	AC	459	LEU
1	AC	463	ASP
1	AC	496	ILE
1	AC	497	GLN
1	AD	127	LEU
1	AD	201(B)	GLU
1	AD	201(E)	ASP
1	AD	201(F)	GLU
1	AD	221	LEU
1	AD	222	VAL
1	AD	243	THR
1	AD	255	ASP
1	AD	260	GLU
1	AD	322	THR
1	AD	323	LEU
1	AD	345	TRP
1	AD	384	ASN
1	AD	395	TYR
1	AD	398	GLN
1	AD	433	TYR
1	AD	453	TYR
1	AD	482	ASN
1	AD	503	ILE
1	AD	507	LEU
1	AD	518	VAL

Continued on next page...

For Manuscript Review

Continued from previous page...

Mol	Chain	Res	Type
1	AE	105	ASN
1	AE	109	PHE
1	AE	114	VAL
1	AE	131	TYR
1	AE	136	VAL
1	AE	154	PHE
1	AE	201(D)	GLU
1	AE	201(E)	ASP
1	AE	201(H)	GLU
1	AE	222	VAL
1	AE	290	LEU
1	AE	300	LEU
1	AE	308	ASP
1	AE	331	VAL
1	AE	340	ILE
1	AE	341	ARG
1	AE	344	ARG
1	AE	345	TRP
1	AE	364	ILE
1	AE	395	TYR
1	AE	453	TYR
1	AE	515	ARG
1	AF	75	ASN
1	AF	106	LEU
1	AF	121	THR
1	AF	127	LEU
1	AF	130	VAL
1	AF	131	TYR
1	AF	170	THR
1	AF	201(B)	GLU
1	AF	201(C)	ASP
1	AF	201(E)	ASP
1	AF	201(G)	ASP
1	AF	230	THR
1	AF	243	THR
1	AF	255	ASP
1	AF	259	ILE
1	AF	313	SER
1	AF	334	PHE
1	AF	338	ILE
1	AF	348	GLU
1	AF	358	ASP

Continued on next page...

For Manuscript Review

Continued from previous page...

Mol	Chain	Res	Type
1	AF	391	THR
1	AF	395	TYR
1	AF	398	GLN
1	AF	400	LEU
1	AF	404	PHE
1	AF	408	THR
1	AF	412	VAL
1	AF	416	VAL
1	AF	435	THR
1	AF	459	LEU
1	AF	466	ASN
1	AF	479	ILE
1	AF	516	VAL
1	AG	99	VAL
1	AG	107	ILE
1	AG	144	PHE
1	AG	166	LEU
1	AG	243	THR
1	AG	254	ILE
1	AG	255	ASP
1	AG	259	ILE
1	AG	264	ARG
1	AG	267	LYS
1	AG	335	GLN
1	AG	388	SER
1	AG	398	GLN
1	AG	416	VAL
1	AG	445	ASP
1	AG	512	TYR
1	AH	72	HIS
1	AH	96	MET
1	AH	105	ASN
1	AH	107	ILE
1	AH	110	ASP
1	AH	114	VAL
1	AH	130	VAL
1	AH	133	LYS
1	AH	147	MET
1	AH	151	ASP
1	AH	170	THR
1	AH	187	THR
1	AH	201(F)	GLU

Continued on next page...

For Manuscript Review

Continued from previous page...

Mol	Chain	Res	Type
1	AH	201(H)	GLU
1	AH	230	THR
1	AH	239	PHE
1	AH	240	ASN
1	AH	247	TRP
1	AH	258	VAL
1	AH	259	ILE
1	AH	274	LEU
1	AH	277	ASP
1	AH	278	LEU
1	AH	300	LEU
1	AH	341	ARG
1	AH	344	ARG
1	AH	345	TRP
1	AH	395	TYR
1	AH	412	VAL
1	AH	425	ILE
1	AH	460	ARG
1	AH	465	LYS
1	AH	468	GLN
1	AH	505	ASN
1	AH	519	LYS
1	AI	68	ILE
1	AI	109	PHE
1	AI	114	VAL
1	AI	130	VAL
1	AI	131	TYR
1	AI	154	PHE
1	AI	185	THR
1	AI	199	ASP
1	AI	201(B)	GLU
1	AI	209	ASP
1	AI	252	PHE
1	AI	260	GLU
1	AI	267	LYS
1	AI	277	ASP
1	AI	285	ASP
1	AI	299	MET
1	AI	300	LEU
1	AI	304	ARG
1	AI	308	ASP
1	AI	331	VAL

Continued on next page...

For Manuscript Review

Continued from previous page...

Mol	Chain	Res	Type
1	AI	334	PHE
1	AI	341	ARG
1	AI	344	ARG
1	AI	345	TRP
1	AI	356	GLN
1	AI	395	TYR
1	AI	425	ILE
1	AI	470	VAL
1	AI	476	ARG
1	AI	507	LEU
1	AI	519	LYS
1	AI	521	ILE
1	AJ	95	VAL
1	AJ	96	MET
1	AJ	99	VAL
1	AJ	100	ARG
1	AJ	105	ASN
1	AJ	109	PHE
1	AJ	121	THR
1	AJ	128	ARG
1	AJ	131	TYR
1	AJ	170	THR
1	AJ	197	THR
1	AJ	201(D)	GLU
1	AJ	255	ASP
1	AJ	256	LYS
1	AJ	259	ILE
1	AJ	334	PHE
1	AJ	348	GLU
1	AJ	395	TYR
1	AJ	404	PHE
1	AJ	425	ILE
1	AJ	443	GLU
1	AJ	444	MET
1	AJ	459	LEU
1	AJ	466	ASN
1	AK	75	ASN
1	AK	107	ILE
1	AK	109	PHE
1	AK	127	LEU
1	AK	128	ARG
1	AK	131	TYR

Continued on next page...

For Manuscript Review

Continued from previous page...

Mol	Chain	Res	Type
1	AK	144	PHE
1	AK	166	LEU
1	AK	195	GLN
1	AK	209	ASP
1	AK	254	ILE
1	AK	272	ILE
1	AK	274	LEU
1	AK	305	GLU
1	AK	385	VAL
1	AK	412	VAL
1	AK	417	LEU
1	AK	512	TYR
1	AL	71	ASP
1	AL	114	VAL
1	AL	127	LEU
1	AL	130	VAL
1	AL	154	PHE
1	AL	161	LYS
1	AL	171	GLN
1	AL	201(A)	ASP
1	AL	201(G)	ASP
1	AL	201(H)	GLU
1	AL	201(I)	ASP
1	AL	243	THR
1	AL	245	ASN
1	AL	282	HIS
1	AL	308	ASP
1	AL	333	ASP
1	AL	344	ARG
1	AL	376	ILE
1	AL	384	ASN
1	AL	395	TYR
1	AL	398	GLN
1	AL	404	PHE
1	AL	431	GLN
1	AL	453	TYR
1	AL	503	ILE
1	AL	507	LEU
1	AM	71	ASP
1	AM	106	LEU
1	AM	109	PHE
1	AM	131	TYR

Continued on next page...

For Manuscript Review

Continued from previous page...

Mol	Chain	Res	Type
1	AM	133	LYS
1	AM	161	LYS
1	AM	166	LEU
1	AM	176	ASP
1	AM	187	THR
1	AM	195	GLN
1	AM	196	VAL
1	AM	221	LEU
1	AM	236	GLN
1	AM	255	ASP
1	AM	256	LYS
1	AM	259	ILE
1	AM	335	GLN
1	AM	374	ASN
1	AM	398	GLN
1	AM	412	VAL
1	AM	459	LEU
1	AM	497	GLN
1	AN	182	PHE
1	AN	289	GLU
1	AN	304	ARG
1	AN	308	ASP
1	AN	339	ASP
1	AN	344	ARG
1	AN	364	ILE
1	AN	374	ASN
1	AN	377	ILE
1	AN	398	GLN
1	AN	503	ILE
1	AN	507	LEU
1	AO	72	HIS
1	AO	109	PHE
1	AO	114	VAL
1	AO	166	LEU
1	AO	201(B)	GLU
1	AO	201(E)	ASP
1	AO	201(F)	GLU
1	AO	203	THR
1	AO	221	LEU
1	AO	222	VAL
1	AO	259	ILE
1	AO	395	TYR

Continued on next page...

For Manuscript Review

Continued from previous page...

Mol	Chain	Res	Type
1	AO	412	VAL
1	AO	443	GLU
1	AO	454	VAL
1	AP	68	ILE
1	AP	84	THR
1	AP	95	VAL
1	AP	101	ARG
1	AP	128	ARG
1	AP	131	TYR
1	AP	221	LEU
1	AP	255	ASP
1	AP	273	GLU
1	AP	323	LEU
1	AP	381	ASN
1	AP	395	TYR
1	AP	421	TYR
1	AP	465	LYS
1	AP	490	GLN
1	AP	507	LEU
1	AQ	72	HIS
1	AQ	114	VAL
1	AQ	123	GLN
1	AQ	127	LEU
1	AQ	131	TYR
1	AQ	136	VAL
1	AQ	170	THR
1	AQ	197	THR
1	AQ	203	THR
1	AQ	258	VAL
1	AQ	260	GLU
1	AQ	272	ILE
1	AQ	331	VAL
1	AQ	344	ARG
1	AQ	345	TRP
1	AQ	348	GLU
1	AQ	395	TYR
1	AQ	408	THR
1	AQ	409	THR
1	AQ	427	GLN
1	AQ	445	ASP
1	AQ	465	LYS
1	AQ	470	VAL

Continued on next page...

For Manuscript Review

Continued from previous page...

Mol	Chain	Res	Type
1	AQ	486	GLU
1	AQ	519	LYS
1	AR	99	VAL
1	AR	107	ILE
1	AR	110	ASP
1	AR	121	THR
1	AR	131	TYR
1	AR	136	VAL
1	AR	170	THR
1	AR	201(C)	ASP
1	AR	201(D)	GLU
1	AR	222	VAL
1	AR	230	THR
1	AR	239	PHE
1	AR	259	ILE
1	AR	313	SER
1	AR	334	PHE
1	AR	338	ILE
1	AR	395	TYR
1	AR	400	LEU
1	AR	404	PHE
1	AR	412	VAL
1	AR	459	LEU
1	AS	72	HIS
1	AS	78	ASN
1	AS	99	VAL
1	AS	107	ILE
1	AS	144	PHE
1	AS	183	GLN
1	AS	243	THR
1	AS	254	ILE
1	AS	323	LEU
1	AS	384	ASN
1	AS	385	VAL
1	AS	428	TYR
1	AS	445	ASP
1	AS	479	ILE
1	AS	512	TYR
1	AT	72	HIS
1	AT	79	ILE
1	AT	114	VAL
1	AT	130	VAL

Continued on next page...

For Manuscript Review

Continued from previous page...

Mol	Chain	Res	Type
1	AT	133	LYS
1	AT	170	THR
1	AT	184	GLU
1	AT	187	THR
1	AT	197	THR
1	AT	201(A)	ASP
1	AT	201(B)	GLU
1	AT	201(F)	GLU
1	AT	201(H)	GLU
1	AT	239	PHE
1	AT	252	PHE
1	AT	273	GLU
1	AT	277	ASP
1	AT	331	VAL
1	AT	341	ARG
1	AT	344	ARG
1	AT	345	TRP
1	AT	348	GLU
1	AT	395	TYR
1	AT	408	THR
1	AT	412	VAL
1	AT	425	ILE
1	AT	460	ARG
1	AT	465	LYS
1	AT	468	GLN
1	AT	507	LEU
1	AT	515	ARG
1	AT	519	LYS
1	AU	77	THR
1	AU	110	ASP
1	AU	114	VAL
1	AU	123	GLN
1	AU	131	TYR
1	AU	170	THR
1	AU	252	PHE
1	AU	260	GLU
1	AU	265	GLN
1	AU	267	LYS
1	AU	277	ASP
1	AU	281	VAL
1	AU	300	LEU
1	AU	308	ASP

Continued on next page...

For Manuscript Review

Continued from previous page...

Mol	Chain	Res	Type
1	AU	331	VAL
1	AU	334	PHE
1	AU	341	ARG
1	AU	344	ARG
1	AU	345	TRP
1	AU	395	TYR
1	AU	412	VAL
1	AU	453	TYR
1	AU	476	ARG
1	AU	514	ARG
1	AV	77	THR
1	AV	95	VAL
1	AV	99	VAL
1	AV	105	ASN
1	AV	107	ILE
1	AV	109	PHE
1	AV	121	THR
1	AV	130	VAL
1	AV	131	TYR
1	AV	170	THR
1	AV	187	THR
1	AV	197	THR
1	AV	201(D)	GLU
1	AV	201(H)	GLU
1	AV	256	LYS
1	AV	259	ILE
1	AV	299	MET
1	AV	334	PHE
1	AV	338	ILE
1	AV	395	TYR
1	AV	398	GLN
1	AV	400	LEU
1	AV	404	PHE
1	AV	408	THR
1	AV	412	VAL
1	AV	417	LEU
1	AV	459	LEU
1	AV	516	VAL
1	AW	72	HIS
1	AW	75	ASN
1	AW	107	ILE
1	AW	144	PHE

Continued on next page...

For Manuscript Review

Continued from previous page...

Mol	Chain	Res	Type
1	AW	255	ASP
1	AW	259	ILE
1	AW	272	ILE
1	AW	379	SER
1	AW	385	VAL
1	AW	398	GLN
1	AW	412	VAL
1	AW	423	VAL
1	AW	466	ASN
1	AW	512	TYR
1	AX	71	ASP
1	AX	130	VAL
1	AX	133	LYS
1	AX	154	PHE
1	AX	161	LYS
1	AX	195	GLN
1	AX	199	ASP
1	AX	201(A)	ASP
1	AX	201(I)	ASP
1	AX	234	GLU
1	AX	237	GLU
1	AX	240	ASN
1	AX	243	THR
1	AX	245	ASN
1	AX	279	ARG
1	AX	282	HIS
1	AX	331	VAL
1	AX	344	ARG
1	AX	364	ILE
1	AX	376	ILE
1	AX	377	ILE
1	AX	395	TYR
1	AX	398	GLN
1	AX	448	ILE
1	AX	503	ILE
1	AX	507	LEU
1	AY	106	LEU
1	AY	131	TYR
1	AY	133	LYS
1	AY	161	LYS
1	AY	162	LYS
1	AY	166	LEU

Continued on next page...

For Manuscript Review

Continued from previous page...

Mol	Chain	Res	Type
1	AY	172	THR
1	AY	176	ASP
1	AY	195	GLN
1	AY	199	ASP
1	AY	201(H)	GLU
1	AY	221	LEU
1	AY	243	THR
1	AY	255	ASP
1	AY	256	LYS
1	AY	259	ILE
1	AY	324	THR
1	AY	356	GLN
1	AY	372	GLU
1	AY	374	ASN
1	AY	412	VAL
1	AY	430	LYS
1	AY	459	LEU
1	AY	497	GLN
1	AZ	243	THR
1	AZ	324	THR
1	AZ	344	ARG
1	AZ	377	ILE
1	AZ	384	ASN
1	AZ	428	TYR
1	AZ	431	GLN
1	AZ	445	ASP
1	AZ	503	ILE
1	AZ	507	LEU
1	Aa	75	ASN
1	Aa	84	THR
1	Aa	106	LEU
1	Aa	109	PHE
1	Aa	110	ASP
1	Aa	136	VAL
1	Aa	221	LEU
1	Aa	226	GLU
1	Aa	255	ASP
1	Aa	345	TRP
1	Aa	398	GLN
1	Aa	430	LYS
1	Aa	504	LEU
1	Ab	68	ILE

Continued on next page...

For Manuscript Review

Continued from previous page...

Mol	Chain	Res	Type
1	Ab	75	ASN
1	Ab	95	VAL
1	Ab	99	VAL
1	Ab	101	ARG
1	Ab	194	VAL
1	Ab	201(A)	ASP
1	Ab	201(B)	GLU
1	Ab	201(G)	ASP
1	Ab	209	ASP
1	Ab	221	LEU
1	Ab	224	ILE
1	Ab	260	GLU
1	Ab	272	ILE
1	Ab	273	GLU
1	Ab	300	LEU
1	Ab	322	THR
1	Ab	395	TYR
1	Ab	459	LEU
1	Ab	482	ASN
1	Ab	507	LEU
1	Ac	114	VAL
1	Ac	170	THR
1	Ac	201(D)	GLU
1	Ac	201(H)	GLU
1	Ac	203	THR
1	Ac	222	VAL
1	Ac	260	GLU
1	Ac	277	ASP
1	Ac	300	LEU
1	Ac	331	VAL
1	Ac	341	ARG
1	Ac	344	ARG
1	Ac	345	TRP
1	Ac	395	TYR
1	Ac	428	TYR
1	Ac	445	ASP
1	Ac	487	SER
1	Ac	507	LEU
1	Ac	515	ARG
1	Ac	521	ILE
1	Ad	84	THR
1	Ad	88	VAL

Continued on next page...

For Manuscript Review

Continued from previous page...

Mol	Chain	Res	Type
1	Ad	105	ASN
1	Ad	121	THR
1	Ad	127	LEU
1	Ad	131	TYR
1	Ad	170	THR
1	Ad	252	PHE
1	Ad	255	ASP
1	Ad	271	SER
1	Ad	366	ARG
1	Ad	375	PHE
1	Ad	395	TYR
1	Ad	400	LEU
1	Ad	459	LEU
1	Ad	466	ASN
1	Ad	510	ASN
1	Ae	72	HIS
1	Ae	109	PHE
1	Ae	114	VAL
1	Ae	127	LEU
1	Ae	144	PHE
1	Ae	201(F)	GLU
1	Ae	203	THR
1	Ae	230	THR
1	Ae	232	ILE
1	Ae	254	ILE
1	Ae	259	ILE
1	Ae	300	LEU
1	Ae	335	GLN
1	Ae	398	GLN
1	Ae	412	VAL
1	Ae	512	TYR
1	Af	67	GLU
1	Af	75	ASN
1	Af	79	ILE
1	Af	96	MET
1	Af	109	PHE
1	Af	114	VAL
1	Af	133	LYS
1	Af	151	ASP
1	Af	170	THR
1	Af	197	THR
1	Af	201(C)	ASP

Continued on next page...

For Manuscript Review

Continued from previous page...

Mol	Chain	Res	Type
1	Af	201(D)	GLU
1	Af	201(G)	ASP
1	Af	201(H)	GLU
1	Af	222	VAL
1	Af	230	THR
1	Af	239	PHE
1	Af	240	ASN
1	Af	244	ASP
1	Af	247	TRP
1	Af	252	PHE
1	Af	260	GLU
1	Af	273	GLU
1	Af	277	ASP
1	Af	278	LEU
1	Af	313	SER
1	Af	341	ARG
1	Af	344	ARG
1	Af	345	TRP
1	Af	348	GLU
1	Af	395	TYR
1	Af	412	VAL
1	Af	425	ILE
1	Af	444	MET
1	Af	465	LYS
1	Af	466	ASN
1	Af	507	LEU
1	Ag	68	ILE
1	Ag	109	PHE
1	Ag	110	ASP
1	Ag	154	PHE
1	Ag	201(B)	GLU
1	Ag	243	THR
1	Ag	244	ASP
1	Ag	252	PHE
1	Ag	260	GLU
1	Ag	267	LYS
1	Ag	277	ASP
1	Ag	290	LEU
1	Ag	299	MET
1	Ag	341	ARG
1	Ag	344	ARG
1	Ag	345	TRP

Continued on next page...

For Manuscript Review

Continued from previous page...

Mol	Chain	Res	Type
1	Ag	395	TYR
1	Ag	432	ASP
1	Ag	442	ASN
1	Ag	445	ASP
1	Ag	453	TYR
1	Ag	476	ARG
1	Ah	90	GLN
1	Ah	95	VAL
1	Ah	96	MET
1	Ah	105	ASN
1	Ah	107	ILE
1	Ah	121	THR
1	Ah	131	TYR
1	Ah	201(B)	GLU
1	Ah	201(C)	ASP
1	Ah	201(D)	GLU
1	Ah	201(H)	GLU
1	Ah	230	THR
1	Ah	256	LYS
1	Ah	259	ILE
1	Ah	274	LEU
1	Ah	281	VAL
1	Ah	313	SER
1	Ah	334	PHE
1	Ah	338	ILE
1	Ah	375	PHE
1	Ah	395	TYR
1	Ah	398	GLN
1	Ah	408	THR
1	Ah	417	LEU
1	Ah	459	LEU
1	Ah	516	VAL
1	Ai	75	ASN
1	Ai	107	ILE
1	Ai	109	PHE
1	Ai	114	VAL
1	Ai	131	TYR
1	Ai	144	PHE
1	Ai	195	GLN
1	Ai	216	MET
1	Ai	236	GLN
1	Ai	244	ASP

Continued on next page...

For Manuscript Review

Continued from previous page...

Mol	Chain	Res	Type
1	Ai	254	ILE
1	Ai	272	ILE
1	Ai	305	GLU
1	Ai	323	LEU
1	Ai	358	ASP
1	Ai	395	TYR
1	Ai	398	GLN
1	Ai	412	VAL
1	Ai	456	LEU
1	Ai	457	THR
1	Ai	512	TYR
1	Aj	71	ASP
1	Aj	84	THR
1	Aj	133	LYS
1	Aj	154	PHE
1	Aj	161	LYS
1	Aj	187	THR
1	Aj	194	VAL
1	Aj	222	VAL
1	Aj	240	ASN
1	Aj	243	THR
1	Aj	245	ASN
1	Aj	279	ARG
1	Aj	282	HIS
1	Aj	304	ARG
1	Aj	339	ASP
1	Aj	344	ARG
1	Aj	377	ILE
1	Aj	398	GLN
1	Aj	404	PHE
1	Aj	427	GLN
1	Aj	445	ASP
1	Aj	503	ILE
1	Ak	68	ILE
1	Ak	109	PHE
1	Ak	114	VAL
1	Ak	133	LYS
1	Ak	161	LYS
1	Ak	166	LEU
1	Ak	196	VAL
1	Ak	221	LEU
1	Ak	243	THR

Continued on next page...

For Manuscript Review

Continued from previous page...

Mol	Chain	Res	Type
1	Ak	255	ASP
1	Ak	256	LYS
1	Ak	259	ILE
1	Ak	285	ASP
1	Ak	398	GLN
1	Ak	459	LEU
1	Ak	519	LYS
1	Al	84	THR
1	Al	136	VAL
1	Al	166	LEU
1	Al	182	PHE
1	Al	199	ASP
1	Al	274	LEU
1	Al	304	ARG
1	Al	322	THR
1	Al	344	ARG
1	Al	377	ILE
1	Al	402	THR
1	Al	490	GLN
1	Al	503	ILE
1	Am	72	HIS
1	Am	109	PHE
1	Am	221	LEU
1	Am	259	ILE
1	Am	285	ASP
1	Am	324	THR
1	Am	334	PHE
1	Am	372	GLU
1	Am	412	VAL
1	Am	459	LEU
2	B	12	THR
2	B	13	THR
2	B	15	ASN
2	B	19	ILE
2	B	28	THR
2	B	32	THR
2	B	34	LEU
2	B	53	TYR
2	B	55	ILE
2	B	87	LEU
2	B	92	LYS
2	B	93	LEU

Continued on next page...

For Manuscript Review

Continued from previous page...

Mol	Chain	Res	Type
2	B	102	LYS
2	B	109	LYS
2	B	136	LYS
2	B	146	THR
2	B	177	THR
2	B	179	ILE
2	B	180	THR
2	B	220	THR
2	B	266	THR
2	B	268	THR
2	B	288	LEU
2	B	289	LYS
2	B	291	LYS
2	B	296	LYS
2	B	298	LEU
2	B	303	TYR
2	B	310	LEU
2	B	320	LEU
2	B	352	ASP
2	B	367	GLU
2	B	384	ASN
2	B	390	LYS
2	B	391	ASP
2	B	396	ARG
2	C	13	THR
2	C	15	ASN
2	C	32	THR
2	C	34	LEU
2	C	45	THR
2	C	47	GLN
2	C	53	TYR
2	C	87	LEU
2	C	92	LYS
2	C	93	LEU
2	C	100	LEU
2	C	107	VAL
2	C	109	LYS
2	C	125	GLU
2	C	136	LYS
2	C	177	THR
2	C	180	THR
2	C	188	GLU

Continued on next page...

For Manuscript Review

Continued from previous page...

Mol	Chain	Res	Type
2	C	197	PHE
2	C	201	LEU
2	C	231	THR
2	C	268	THR
2	C	288	LEU
2	C	289	LYS
2	C	291	LYS
2	C	294	ASP
2	C	296	LYS
2	C	298	LEU
2	C	303	TYR
2	C	306	LEU
2	C	308	ASN
2	C	320	LEU
2	C	327	VAL
2	C	337	VAL
2	C	353	ASP
2	C	384	ASN
2	C	390	LYS
2	C	391	ASP
2	C	405	LYS
2	C	414	VAL
2	D	25	VAL
2	D	29	ARG
2	D	32	THR
2	D	38	ASP
2	D	45	THR
2	D	53	TYR
2	D	55	ILE
2	D	87	LEU
2	D	92	LYS
2	D	93	LEU
2	D	108	TYR
2	D	109	LYS
2	D	125	GLU
2	D	177	THR
2	D	180	THR
2	D	187	LEU
2	D	230	ILE
2	D	231	THR
2	D	236	ILE
2	D	266	THR

Continued on next page...

For Manuscript Review

Continued from previous page...

Mol	Chain	Res	Type
2	D	268	THR
2	D	288	LEU
2	D	289	LYS
2	D	291	LYS
2	D	295	ASP
2	D	296	LYS
2	D	298	LEU
2	D	301	ASN
2	D	310	LEU
2	D	320	LEU
2	D	337	VAL
2	D	352	ASP
2	D	367	GLU
2	D	390	LYS
2	D	391	ASP
2	D	396	ARG
2	D	405	LYS
1	G	68	ILE
1	G	90	GLN
1	G	109	PHE
1	G	121	THR
1	G	131	TYR
1	G	151	ASP
1	G	170	THR
1	G	197	THR
1	G	201(E)	ASP
1	G	201(H)	GLU
1	G	201(I)	ASP
1	G	222	VAL
1	G	226	GLU
1	G	244	ASP
1	G	256	LYS
1	G	259	ILE
1	G	313	SER
1	G	334	PHE
1	G	344	ARG
1	G	395	TYR
1	G	400	LEU
1	G	404	PHE
1	G	457	THR
1	G	459	LEU
1	G	471	MET

Continued on next page...

For Manuscript Review

Continued from previous page...

Mol	Chain	Res	Type
1	H	72	HIS
1	H	109	PHE
1	H	128	ARG
1	H	131	TYR
1	H	144	PHE
1	H	195	GLN
1	H	201(H)	GLU
1	H	245	ASN
1	H	254	ILE
1	H	259	ILE
1	H	272	ILE
1	H	273	GLU
1	H	305	GLU
1	H	323	LEU
1	H	331	VAL
1	H	345	TRP
1	H	385	VAL
1	H	423	VAL
1	H	445	ASP
1	H	505	ASN
1	H	512	TYR
1	H	518	VAL
1	J	68	ILE
1	J	101	ARG
1	J	106	LEU
1	J	161	LYS
1	J	166	LEU
1	J	187	THR
1	J	201(B)	GLU
1	J	221	LEU
1	J	239	PHE
1	J	249	GLU
1	J	255	ASP
1	J	259	ILE
1	J	274	LEU
1	J	351	LYS
1	J	372	GLU
1	J	412	VAL
1	J	432	ASP
1	J	443	GLU
1	J	459	LEU
1	K	68	ILE

Continued on next page...

For Manuscript Review

Continued from previous page...

Mol	Chain	Res	Type
1	K	72	HIS
1	K	84	THR
1	K	95	VAL
1	K	99	VAL
1	K	101	ARG
1	K	131	TYR
1	K	179	THR
1	K	221	LEU
1	K	237	GLU
1	K	273	GLU
1	K	276	GLN
1	K	279	ARG
1	K	322	THR
1	K	323	LEU
1	K	381	ASN
1	K	395	TYR
1	K	465	LYS
1	K	507	LEU
1	L	109	PHE
1	L	114	VAL
1	L	131	TYR
1	L	201(D)	GLU
1	L	201(I)	ASP
1	L	240	ASN
1	L	260	GLU
1	L	265	GLN
1	L	341	ARG
1	L	344	ARG
1	L	345	TRP
1	L	395	TYR
1	L	445	ASP
1	L	466	ASN
1	M	72	HIS
1	M	109	PHE
1	M	121	THR
1	M	131	TYR
1	M	170	THR
1	M	222	VAL
1	M	239	PHE
1	M	243	THR
1	M	339	ASP
1	M	391	THR

Continued on next page...

For Manuscript Review

Continued from previous page...

Mol	Chain	Res	Type
1	M	395	TYR
1	M	404	PHE
1	M	459	LEU
1	M	481	ILE
1	M	510	ASN
1	N	72	HIS
1	N	107	ILE
1	N	144	PHE
1	N	182	PHE
1	N	243	THR
1	N	308	ASP
1	N	323	LEU
1	N	335	GLN
1	N	385	VAL
1	N	416	VAL
1	N	442	ASN
1	N	445	ASP
1	N	512	TYR
1	O	67	GLU
1	O	72	HIS
1	O	107	ILE
1	O	114	VAL
1	O	130	VAL
1	O	133	LYS
1	O	170	THR
1	O	184	GLU
1	O	187	THR
1	O	195	GLN
1	O	197	THR
1	O	198	ILE
1	O	201(B)	GLU
1	O	222	VAL
1	O	226	GLU
1	O	239	PHE
1	O	240	ASN
1	O	247	TRP
1	O	252	PHE
1	O	260	GLU
1	O	270	TYR
1	O	274	LEU
1	O	278	LEU
1	O	289	GLU

Continued on next page...

For Manuscript Review

Continued from previous page...

Mol	Chain	Res	Type
1	O	331	VAL
1	O	344	ARG
1	O	345	TRP
1	O	348	GLU
1	O	395	TYR
1	O	406	THR
1	O	408	THR
1	O	412	VAL
1	O	425	ILE
1	O	444	MET
1	O	465	LYS
1	O	466	ASN
1	O	468	GLN
1	O	482	ASN
1	O	507	LEU
1	P	89	THR
1	P	110	ASP
1	P	114	VAL
1	P	131	TYR
1	P	136	VAL
1	P	154	PHE
1	P	170	THR
1	P	195	GLN
1	P	201(B)	GLU
1	P	244	ASP
1	P	252	PHE
1	P	260	GLU
1	P	267	LYS
1	P	277	ASP
1	P	308	ASP
1	P	331	VAL
1	P	341	ARG
1	P	344	ARG
1	P	345	TRP
1	P	395	TYR
1	P	436	VAL
1	P	453	TYR
1	P	466	ASN
1	P	476	ARG
1	P	507	LEU
1	P	519	LYS
1	Q	67	GLU

Continued on next page...

For Manuscript Review

Continued from previous page...

Mol	Chain	Res	Type
1	Q	96	MET
1	Q	109	PHE
1	Q	119	SER
1	Q	121	THR
1	Q	127	LEU
1	Q	170	THR
1	Q	183	GLN
1	Q	197	THR
1	Q	201(F)	GLU
1	Q	256	LYS
1	Q	308	ASP
1	Q	313	SER
1	Q	338	ILE
1	Q	344	ARG
1	Q	384	ASN
1	Q	395	TYR
1	Q	400	LEU
1	Q	404	PHE
1	Q	412	VAL
1	Q	417	LEU
1	Q	459	LEU
1	R	75	ASN
1	R	106	LEU
1	R	107	ILE
1	R	109	PHE
1	R	144	PHE
1	R	195	GLN
1	R	243	THR
1	R	259	ILE
1	R	272	ILE
1	R	323	LEU
1	R	335	GLN
1	R	345	TRP
1	R	395	TYR
1	R	398	GLN
1	R	406	THR
1	R	412	VAL
1	R	445	ASP
1	R	466	ASN
1	R	505	ASN
1	R	512	TYR
1	S	71	ASP

Continued on next page...

For Manuscript Review

Continued from previous page...

Mol	Chain	Res	Type
1	S	127	LEU
1	S	130	VAL
1	S	133	LYS
1	S	136	VAL
1	S	154	PHE
1	S	161	LYS
1	S	171	GLN
1	S	195	GLN
1	S	198	ILE
1	S	201(C)	ASP
1	S	201(E)	ASP
1	S	222	VAL
1	S	230	THR
1	S	237	GLU
1	S	245	ASN
1	S	264	ARG
1	S	279	ARG
1	S	282	HIS
1	S	289	GLU
1	S	344	ARG
1	S	395	TYR
1	S	404	PHE
1	S	428	TYR
1	S	445	ASP
1	S	453	TYR
1	S	503	ILE
1	S	507	LEU
1	T	71	ASP
1	T	106	LEU
1	T	114	VAL
1	T	133	LYS
1	T	136	VAL
1	T	147	MET
1	T	161	LYS
1	T	166	LEU
1	T	176	ASP
1	T	199	ASP
1	T	221	LEU
1	T	245	ASN
1	T	253	ARG
1	T	255	ASP
1	T	256	LYS

Continued on next page...

For Manuscript Review

Continued from previous page...

Mol	Chain	Res	Type
1	T	274	LEU
1	T	374	ASN
1	T	400	LEU
1	T	454	VAL
1	T	459	LEU
1	T	465	LYS
1	T	471	MET
1	U	68	ILE
1	U	78	ASN
1	U	79	ILE
1	U	84	THR
1	U	95	VAL
1	U	101	ARG
1	U	201(D)	GLU
1	U	201(F)	GLU
1	U	221	LEU
1	U	255	ASP
1	U	273	GLU
1	U	323	LEU
1	U	349	SER
1	U	395	TYR
1	U	428	TYR
1	U	482	ASN
1	U	503	ILE
1	U	507	LEU
1	V	90	GLN
1	V	110	ASP
1	V	114	VAL
1	V	131	TYR
1	V	187	THR
1	V	198	ILE
1	V	201(C)	ASP
1	V	201(F)	GLU
1	V	222	VAL
1	V	263	SER
1	V	267	LYS
1	V	331	VAL
1	V	341	ARG
1	V	344	ARG
1	V	345	TRP
1	V	395	TYR
1	V	408	THR

Continued on next page...

For Manuscript Review

Continued from previous page...

Mol	Chain	Res	Type
1	V	436	VAL
1	V	470	VAL
1	W	75	ASN
1	W	95	VAL
1	W	107	ILE
1	W	121	THR
1	W	131	TYR
1	W	136	VAL
1	W	170	THR
1	W	222	VAL
1	W	230	THR
1	W	313	SER
1	W	334	PHE
1	W	391	THR
1	W	395	TYR
1	W	404	PHE
1	W	435	THR
1	W	459	LEU
1	W	466	ASN
1	X	107	ILE
1	X	144	PHE
1	X	166	LEU
1	X	230	THR
1	X	254	ILE
1	X	259	ILE
1	X	300	LEU
1	X	323	LEU
1	X	335	GLN
1	X	344	ARG
1	X	398	GLN
1	X	412	VAL
1	X	477	TYR
1	Y	72	HIS
1	Y	96	MET
1	Y	105	ASN
1	Y	107	ILE
1	Y	114	VAL
1	Y	133	LYS
1	Y	170	THR
1	Y	194	VAL
1	Y	197	THR
1	Y	201(A)	ASP

Continued on next page...

For Manuscript Review

Continued from previous page...

Mol	Chain	Res	Type
1	Y	201(D)	GLU
1	Y	201(F)	GLU
1	Y	239	PHE
1	Y	240	ASN
1	Y	247	TRP
1	Y	252	PHE
1	Y	258	VAL
1	Y	270	TYR
1	Y	273	GLU
1	Y	277	ASP
1	Y	287	ASP
1	Y	331	VAL
1	Y	344	ARG
1	Y	345	TRP
1	Y	348	GLU
1	Y	395	TYR
1	Y	408	THR
1	Y	425	ILE
1	Y	427	GLN
1	Y	444	MET
1	Y	465	LYS
1	Y	466	ASN
1	Z	110	ASP
1	Z	114	VAL
1	Z	123	GLN
1	Z	130	VAL
1	Z	131	TYR
1	Z	154	PHE
1	Z	170	THR
1	Z	201(F)	GLU
1	Z	203	THR
1	Z	244	ASP
1	Z	252	PHE
1	Z	260	GLU
1	Z	267	LYS
1	Z	300	LEU
1	Z	321	MET
1	Z	331	VAL
1	Z	341	ARG
1	Z	344	ARG
1	Z	345	TRP
1	Z	389	VAL

Continued on next page...

For Manuscript Review

Continued from previous page...

Mol	Chain	Res	Type
1	Z	395	TYR
1	Z	432	ASP
1	Z	453	TYR
1	Z	476	ARG
1	Z	507	LEU
1	Z	515	ARG
1	Z	518	VAL
1	Z	519	LYS
2	a	15	ASN
2	a	19	ILE
2	a	28	THR
2	a	32	THR
2	a	34	LEU
2	a	47	GLN
2	a	53	TYR
2	a	55	ILE
2	a	87	LEU
2	a	92	LYS
2	a	102	LYS
2	a	109	LYS
2	a	125	GLU
2	a	136	LYS
2	a	161	PHE
2	a	177	THR
2	a	187	LEU
2	a	190	ASN
2	a	197	PHE
2	a	231	THR
2	a	266	THR
2	a	268	THR
2	a	288	LEU
2	a	289	LYS
2	a	291	LYS
2	a	296	LYS
2	a	298	LEU
2	a	320	LEU
2	a	337	VAL
2	a	356	HIS
2	a	367	GLU
2	a	390	LYS
2	a	391	ASP
2	a	405	LYS

Continued on next page...

For Manuscript Review

Continued from previous page...

Mol	Chain	Res	Type
2	a	414	VAL
1	b	67	GLU
1	b	90	GLN
1	b	95	VAL
1	b	96	MET
1	b	109	PHE
1	b	121	THR
1	b	127	LEU
1	b	131	TYR
1	b	170	THR
1	b	199	ASP
1	b	201(F)	GLU
1	b	240	ASN
1	b	313	SER
1	b	324	THR
1	b	334	PHE
1	b	338	ILE
1	b	395	TYR
1	b	398	GLN
1	b	408	THR
1	b	459	LEU
1	b	516	VAL
1	c	107	ILE
1	c	109	PHE
1	c	127	LEU
1	c	128	ARG
1	c	131	TYR
1	c	144	PHE
1	c	195	GLN
1	c	254	ILE
1	c	272	ILE
1	c	323	LEU
1	c	385	VAL
1	c	406	THR
1	c	428	TYR
1	c	512	TYR
1	d	130	VAL
1	d	133	LYS
1	d	161	LYS
1	d	171	GLN
1	d	194	VAL
1	d	195	GLN

Continued on next page...

For Manuscript Review

Continued from previous page...

Mol	Chain	Res	Type
1	d	230	THR
1	d	243	THR
1	d	282	HIS
1	d	304	ARG
1	d	344	ARG
1	d	376	ILE
1	d	377	ILE
1	d	395	TYR
1	d	503	ILE
1	e	109	PHE
1	e	127	LEU
1	e	133	LYS
1	e	161	LYS
1	e	166	LEU
1	e	187	THR
1	e	195	GLN
1	e	221	LEU
1	e	240	ASN
1	e	255	ASP
1	e	256	LYS
1	e	259	ILE
1	e	265	GLN
1	e	285	ASP
1	e	372	GLU
1	e	432	ASP
1	e	459	LEU
1	e	497	GLN
1	f	72	HIS
1	f	89	THR
1	f	107	ILE
1	f	114	VAL
1	f	133	LYS
1	f	151	ASP
1	f	187	THR
1	f	197	THR
1	f	201(G)	ASP
1	f	239	PHE
1	f	240	ASN
1	f	244	ASP
1	f	247	TRP
1	f	252	PHE
1	f	259	ILE

Continued on next page...

For Manuscript Review

Continued from previous page...

Mol	Chain	Res	Type
1	f	260	GLU
1	f	270	TYR
1	f	277	ASP
1	f	341	ARG
1	f	344	ARG
1	f	345	TRP
1	f	348	GLU
1	f	374	ASN
1	f	389	VAL
1	f	395	TYR
1	f	406	THR
1	f	425	ILE
1	f	427	GLN
1	f	430	LYS
1	f	454	VAL
1	f	460	ARG
1	f	465	LYS
1	g	84	THR
1	g	222	VAL
1	g	224	ILE
1	g	273	GLU
1	g	333	ASP
1	g	344	ARG
1	g	377	ILE
1	g	395	TYR
1	g	412	VAL
1	g	503	ILE
1	h	114	VAL
1	h	134	ASP
1	h	176	ASP
1	h	221	LEU
1	h	273	GLU
1	h	374	ASN
1	h	398	GLN
1	h	412	VAL
1	h	430	LYS
1	h	432	ASP
1	h	443	GLU
1	h	448	ILE
1	h	459	LEU
1	i	84	THR
1	i	154	PHE

Continued on next page...

For Manuscript Review

Continued from previous page...

Mol	Chain	Res	Type
1	i	166	LEU
1	i	344	ARG
1	i	395	TYR
1	i	436	VAL
1	i	445	ASP
1	i	482	ASN
1	i	503	ILE
1	i	505	ASN
1	i	507	LEU
1	j	72	HIS
1	j	195	GLN
1	j	201(F)	GLU
1	j	201(I)	ASP
1	j	203	THR
1	j	221	LEU
1	j	259	ILE
1	j	324	THR
1	j	412	VAL
1	j	430	LYS
1	k	68	ILE
1	k	114	VAL
1	k	131	TYR
1	k	136	VAL
1	k	154	PHE
1	k	176	ASP
1	k	197	THR
1	k	199	ASP
1	k	201(A)	ASP
1	k	201(B)	GLU
1	k	203	THR
1	k	252	PHE
1	k	277	ASP
1	k	300	LEU
1	k	308	ASP
1	k	331	VAL
1	k	341	ARG
1	k	344	ARG
1	k	345	TRP
1	k	395	TYR
1	k	476	ARG
1	k	507	LEU
1	k	519	LYS

Continued on next page...

For Manuscript Review

Continued from previous page...

Mol	Chain	Res	Type
1	l	89	THR
1	l	96	MET
1	l	109	PHE
1	l	121	THR
1	l	131	TYR
1	l	201(D)	GLU
1	l	256	LYS
1	l	259	ILE
1	l	274	LEU
1	l	276	GLN
1	l	338	ILE
1	l	395	TYR
1	l	400	LEU
1	l	404	PHE
1	l	412	VAL
1	l	425	ILE
1	l	459	LEU
1	l	482	ASN
1	l	516	VAL
1	m	127	LEU
1	m	131	TYR
1	m	144	PHE
1	m	166	LEU
1	m	201(C)	ASP
1	m	201(D)	GLU
1	m	201(F)	GLU
1	m	201(H)	GLU
1	m	201(I)	ASP
1	m	236	GLN
1	m	244	ASP
1	m	254	ILE
1	m	259	ILE
1	m	272	ILE
1	m	305	GLU
1	m	323	LEU
1	m	335	GLN
1	m	412	VAL
1	m	466	ASN
1	n	71	ASP
1	n	114	VAL
1	n	154	PHE
1	n	161	LYS

Continued on next page...

For Manuscript Review

Continued from previous page...

Mol	Chain	Res	Type
1	n	171	GLN
1	n	191	GLN
1	n	194	VAL
1	n	237	GLU
1	n	243	THR
1	n	245	ASN
1	n	278	LEU
1	n	279	ARG
1	n	282	HIS
1	n	308	ASP
1	n	344	ARG
1	n	377	ILE
1	n	432	ASP
1	n	453	TYR
1	n	503	ILE
1	o	71	ASP
1	o	109	PHE
1	o	114	VAL
1	o	133	LYS
1	o	136	VAL
1	o	147	MET
1	o	151	ASP
1	o	161	LYS
1	o	166	LEU
1	o	176	ASP
1	o	187	THR
1	o	195	GLN
1	o	221	LEU
1	o	255	ASP
1	o	256	LYS
1	o	259	ILE
1	o	344	ARG
1	o	389	VAL
1	o	398	GLN
1	o	412	VAL
1	o	443	GLU
1	o	459	LEU
1	o	497	GLN
1	p	67	GLU
1	p	79	ILE
1	p	105	ASN
1	p	107	ILE

Continued on next page...

For Manuscript Review

Continued from previous page...

Mol	Chain	Res	Type
1	p	130	VAL
1	p	151	ASP
1	p	170	THR
1	p	187	THR
1	p	195	GLN
1	p	197	THR
1	p	198	ILE
1	p	201(A)	ASP
1	p	201(G)	ASP
1	p	222	VAL
1	p	259	ILE
1	p	273	GLU
1	p	274	LEU
1	p	278	LEU
1	p	300	LEU
1	p	341	ARG
1	p	344	ARG
1	p	345	TRP
1	p	348	GLU
1	p	395	TYR
1	p	425	ILE
1	p	444	MET
1	p	467	PHE
1	q	68	ILE
1	q	79	ILE
1	q	96	MET
1	q	107	ILE
1	q	109	PHE
1	q	114	VAL
1	q	131	TYR
1	q	170	THR
1	q	201(B)	GLU
1	q	252	PHE
1	q	267	LYS
1	q	277	ASP
1	q	300	LEU
1	q	308	ASP
1	q	331	VAL
1	q	341	ARG
1	q	344	ARG
1	q	345	TRP
1	q	395	TYR

Continued on next page...

For Manuscript Review

Continued from previous page...

Mol	Chain	Res	Type
1	q	432	ASP
1	q	445	ASP
1	q	470	VAL
1	q	519	LYS
1	r	75	ASN
1	r	96	MET
1	r	101	ARG
1	r	109	PHE
1	r	121	THR
1	r	153	MET
1	r	170	THR
1	r	176	ASP
1	r	197	THR
1	r	204	ASP
1	r	207	LYS
1	r	236	GLN
1	r	256	LYS
1	r	334	PHE
1	r	348	GLU
1	r	395	TYR
1	r	404	PHE
1	r	408	THR
1	r	412	VAL
1	r	417	LEU
1	r	459	LEU
1	r	466	ASN
1	s	75	ASN
1	s	109	PHE
1	s	131	TYR
1	s	144	PHE
1	s	185	THR
1	s	209	ASP
1	s	239	PHE
1	s	272	ILE
1	s	290	LEU
1	s	323	LEU
1	s	385	VAL
1	s	412	VAL
1	s	466	ASN
1	t	68	ILE
1	t	84	THR
1	t	100	ARG

Continued on next page...

For Manuscript Review

Continued from previous page...

Mol	Chain	Res	Type
1	t	101	ARG
1	t	114	VAL
1	t	136	VAL
1	t	154	PHE
1	t	161	LYS
1	t	171	GLN
1	t	182	PHE
1	t	187	THR
1	t	194	VAL
1	t	230	THR
1	t	237	GLU
1	t	240	ASN
1	t	243	THR
1	t	248	ASN
1	t	273	GLU
1	t	279	ARG
1	t	282	HIS
1	t	344	ARG
1	t	374	ASN
1	t	377	ILE
1	t	384	ASN
1	t	398	GLN
1	t	404	PHE
1	t	507	LEU
1	u	101	ARG
1	u	131	TYR
1	u	136	VAL
1	u	161	LYS
1	u	162	LYS
1	u	187	THR
1	u	195	GLN
1	u	201(B)	GLU
1	u	221	LEU
1	u	253	ARG
1	u	255	ASP
1	u	256	LYS
1	u	308	ASP
1	u	398	GLN
1	u	412	VAL
1	u	459	LEU
1	u	497	GLN
1	v	68	ILE

Continued on next page...

For Manuscript Review

Continued from previous page...

Mol	Chain	Res	Type
1	v	78	ASN
1	v	84	THR
1	v	95	VAL
1	v	101	ARG
1	v	172	THR
1	v	221	LEU
1	v	255	ASP
1	v	273	GLU
1	v	323	LEU
1	v	395	TYR
1	v	398	GLN
1	v	482	ASN
1	v	510	ASN
1	w	109	PHE
1	w	114	VAL
1	w	131	TYR
1	w	201(D)	GLU
1	w	201(F)	GLU
1	w	201(H)	GLU
1	w	201(I)	ASP
1	w	277	ASP
1	w	331	VAL
1	w	344	ARG
1	w	345	TRP
1	w	425	ILE
1	w	430	LYS
1	w	432	ASP
1	w	448	ILE
1	w	466	ASN
1	x	105	ASN
1	x	107	ILE
1	x	109	PHE
1	x	119	SER
1	x	121	THR
1	x	131	TYR
1	x	201(C)	ASP
1	x	230	THR
1	x	237	GLU
1	x	239	PHE
1	x	334	PHE
1	x	395	TYR
1	x	404	PHE

Continued on next page...

For Manuscript Review

Continued from previous page...

Mol	Chain	Res	Type
1	x	408	THR
1	x	412	VAL
1	x	459	LEU
1	x	516	VAL
1	y	72	HIS
1	y	107	ILE
1	y	127	LEU
1	y	195	GLN
1	y	204	ASP
1	y	230	THR
1	y	243	THR
1	y	245	ASN
1	y	254	ILE
1	y	259	ILE
1	y	264	ARG
1	y	267	LYS
1	y	300	LEU
1	y	323	LEU
1	y	344	ARG
1	y	398	GLN
1	y	412	VAL
1	y	416	VAL
1	y	445	ASP
1	y	512	TYR
1	z	75	ASN
1	z	79	ILE
1	z	105	ASN
1	z	107	ILE
1	z	114	VAL
1	z	130	VAL
1	z	133	LYS
1	z	170	THR
1	z	173	THR
1	z	187	THR
1	z	195	GLN
1	z	197	THR
1	z	201(A)	ASP
1	z	201(C)	ASP
1	z	201(H)	GLU
1	z	214	LYS
1	z	222	VAL
1	z	226	GLU

Continued on next page...

For Manuscript Review

Continued from previous page...

Mol	Chain	Res	Type
1	z	235	LEU
1	z	239	PHE
1	z	252	PHE
1	z	259	ILE
1	z	260	GLU
1	z	277	ASP
1	z	300	LEU
1	z	331	VAL
1	z	344	ARG
1	z	345	TRP
1	z	348	GLU
1	z	386	LEU
1	z	395	TYR
1	z	406	THR
1	z	412	VAL
1	z	425	ILE
1	z	454	VAL
1	z	460	ARG
1	z	465	LYS
1	z	466	ASN
1	z	507	LEU
1	z	514	ARG

Sometimes sidechains can be flipped to improve hydrogen bonding and reduce clashes. All (141) such sidechains are listed below:

Mol	Chain	Res	Type
1	0	90	GLN
1	0	265	GLN
1	0	356	GLN
1	1	145	HIS
1	1	171	GLN
1	1	195	GLN
1	1	265	GLN
1	1	468	GLN
1	2	236	GLN
1	2	257	GLN
1	2	497	GLN
1	4	245	ASN
1	5	505	ASN
1	6	257	GLN
1	7	171	GLN
1	7	195	GLN

Continued on next page...

For Manuscript Review

Continued from previous page...

Mol	Chain	Res	Type
1	8	245	ASN
1	8	257	GLN
1	8	468	GLN
1	8	497	GLN
1	9	72	HIS
1	9	303	ASN
2	A	384	ASN
1	AA	171	GLN
1	AA	195	GLN
1	AA	240	ASN
1	AA	245	ASN
1	AB	245	ASN
1	AB	265	GLN
1	AB	482	ASN
1	AC	236	GLN
1	AC	265	GLN
1	AC	505	ASN
1	AE	90	GLN
1	AE	236	GLN
1	AE	315	GLN
1	AF	236	GLN
1	AF	398	GLN
1	AG	240	ASN
1	AH	145	HIS
1	AH	195	GLN
1	AI	265	GLN
1	AJ	75	ASN
1	AK	171	GLN
1	AK	195	GLN
1	AL	245	ASN
1	AM	265	GLN
1	AN	265	GLN
1	AN	276	GLN
1	AO	236	GLN
1	AO	240	ASN
1	AP	72	HIS
1	AP	115	GLN
1	AP	282	HIS
1	AQ	265	GLN
1	AS	240	ASN
1	AT	195	GLN
1	AU	171	GLN

Continued on next page...

For Manuscript Review

Continued from previous page...

Mol	Chain	Res	Type
1	AU	195	GLN
1	AU	265	GLN
1	AU	315	GLN
1	AU	482	ASN
1	AV	72	HIS
1	AW	257	GLN
1	AX	115	GLN
1	AX	482	ASN
1	Aa	171	GLN
1	Aa	195	GLN
1	Ab	105	ASN
1	Ab	257	GLN
1	Ab	282	HIS
1	Ac	171	GLN
1	Ac	195	GLN
1	Ac	482	ASN
1	Ae	171	GLN
1	Ae	195	GLN
1	Ae	240	ASN
1	Af	195	GLN
1	Af	482	ASN
1	Ah	265	GLN
1	Ai	236	GLN
1	Aj	115	GLN
1	Aj	245	ASN
1	Ak	265	GLN
2	B	384	ASN
2	C	190	ASN
2	C	384	ASN
2	D	384	ASN
1	G	236	GLN
1	G	245	ASN
1	J	123	GLN
1	J	265	GLN
1	K	431	GLN
1	K	482	ASN
1	L	171	GLN
1	L	195	GLN
1	L	276	GLN
1	L	303	ASN
1	N	115	GLN
1	N	240	ASN

Continued on next page...

For Manuscript Review

Continued from previous page...

Mol	Chain	Res	Type
1	P	265	GLN
1	Q	183	GLN
1	Q	265	GLN
1	S	245	ASN
1	S	431	GLN
1	T	505	ASN
1	V	171	GLN
1	V	195	GLN
1	V	303	ASN
1	W	482	ASN
1	Y	482	ASN
2	a	384	ASN
1	b	265	GLN
1	c	482	ASN
1	d	191	GLN
1	e	171	GLN
1	e	195	GLN
1	e	236	GLN
1	e	265	GLN
1	f	195	GLN
1	f	315	GLN
1	g	195	GLN
1	h	265	GLN
1	i	497	GLN
1	j	123	GLN
1	k	303	ASN
1	l	236	GLN
1	l	265	GLN
1	m	236	GLN
1	m	265	GLN
1	o	236	GLN
1	p	115	GLN
1	p	466	ASN
1	p	482	ASN
1	r	90	GLN
1	r	257	GLN
1	s	240	ASN
1	t	497	GLN
1	v	282	HIS
1	w	236	GLN
1	x	265	GLN

For Manuscript Review

5.3.3 RNA (i)

There are no RNA molecules in this entry.

5.4 Non-standard residues in protein, DNA, RNA chains (i)

There are no non-standard protein/DNA/RNA residues in this entry.

5.5 Carbohydrates (i)

There are no monosaccharides in this entry.

5.6 Ligand geometry (i)

There are no ligands in this entry.

5.7 Other polymers (i)

There are no such residues in this entry.

5.8 Polymer linkage issues (i)

There are no chain breaks in this entry.

For Manuscript Review

6 Map visualisation (i)

This section contains visualisations of the EMDB entry EMD-40228. These allow visual inspection of the internal detail of the map and identification of artifacts.

Images derived from a raw map, generated by summing the deposited half-maps, are presented below the corresponding image components of the primary map to allow further visual inspection and comparison with those of the primary map.

6.1 Orthogonal projections (i)

6.1.1 Primary map

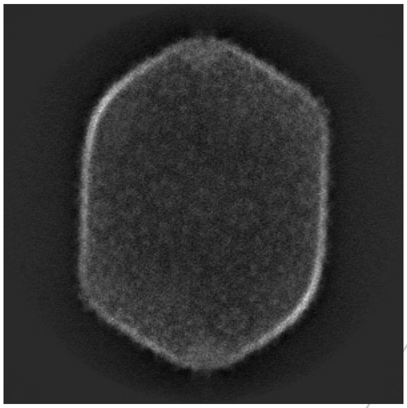
X

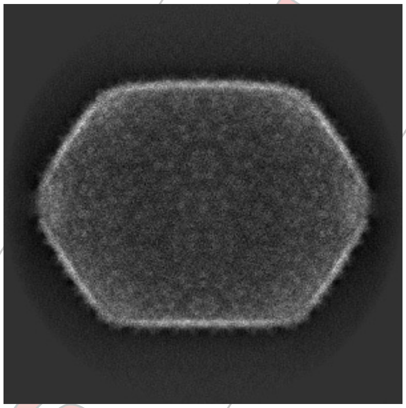
Y

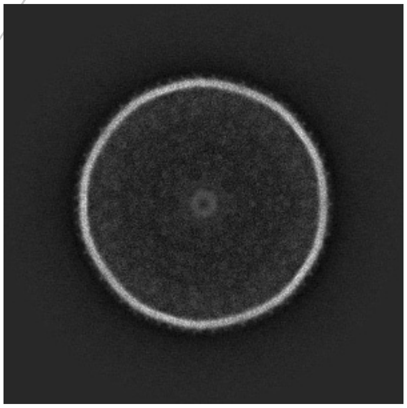
Z

6.1.2 Raw map

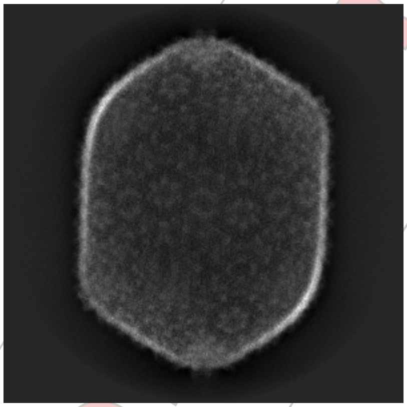
X

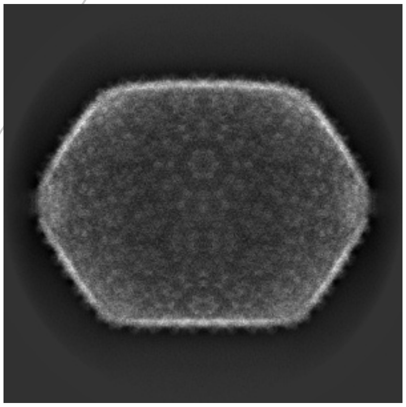
Y

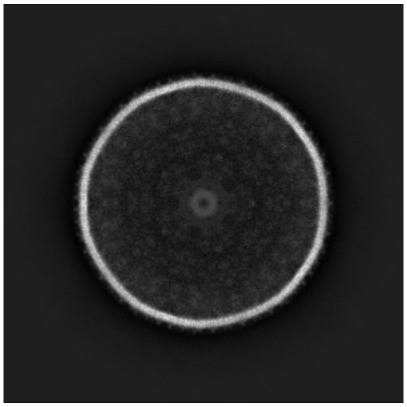
Z

The images above show the map projected in three orthogonal directions.

For Manuscript Review

6.2 Central slices (i)

6.2.1 Primary map

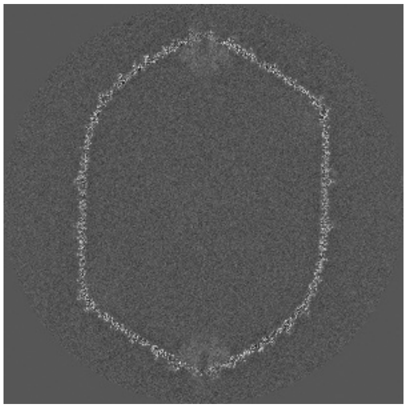

X Index: 360

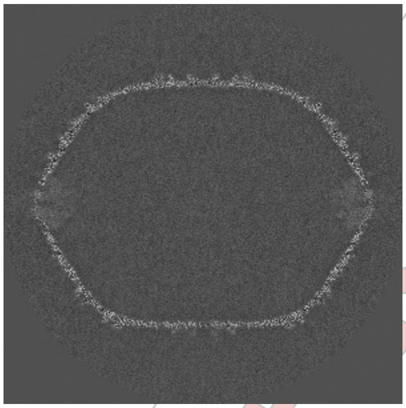

Y Index: 360

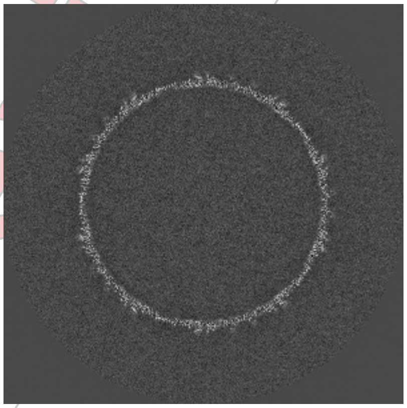

Z Index: 360

6.2.2 Raw map

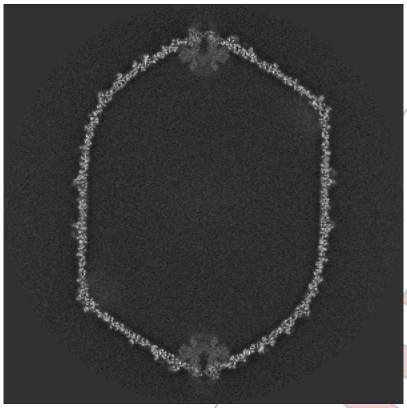

X Index: 360

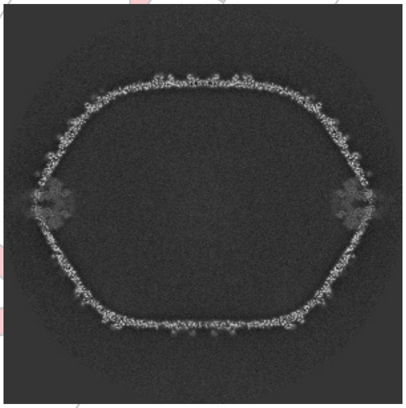

Y Index: 360

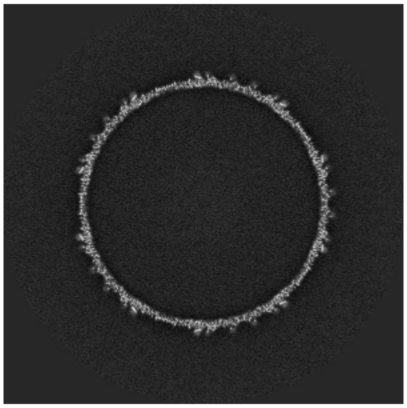

Z Index: 360

The images above show central slices of the map in three orthogonal directions.

For Manuscript Review

6.3 Largest variance slices (i)

6.3.1 Primary map

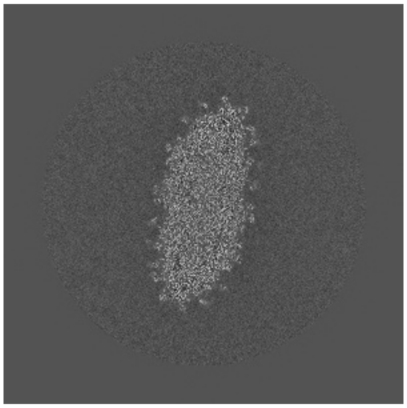
X Index: 147

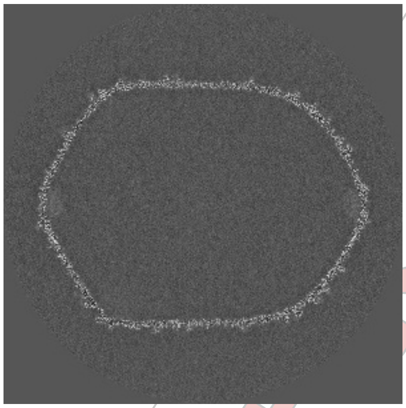
Y Index: 323

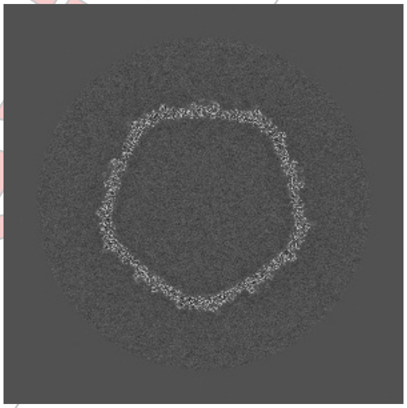
Z Index: 157

6.3.2 Raw map

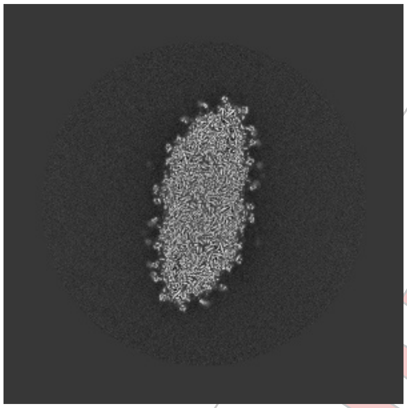
X Index: 147

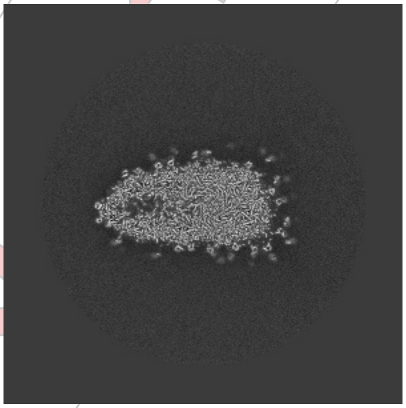
Y Index: 147

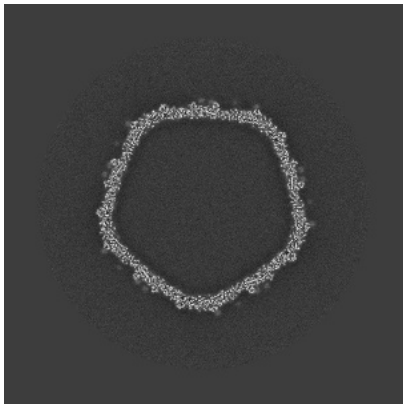
Z Index: 157

The images above show the largest variance slices of the map in three orthogonal directions.

For Manuscript Review

6.4 Orthogonal standard-deviation projections (False-color) (i)

6.4.1 Primary map

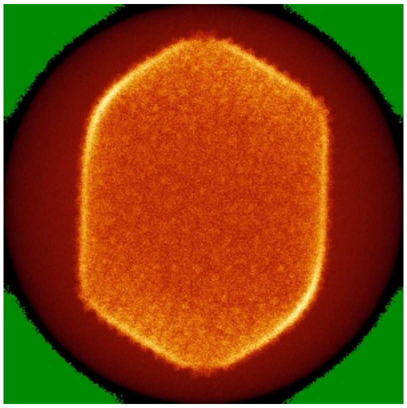
X

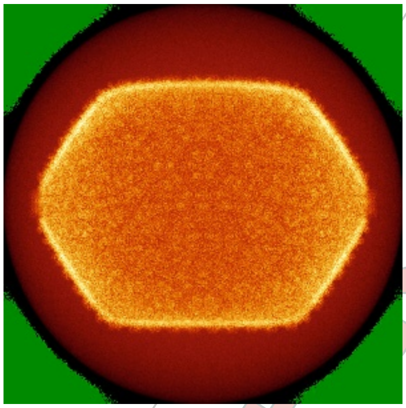
Y

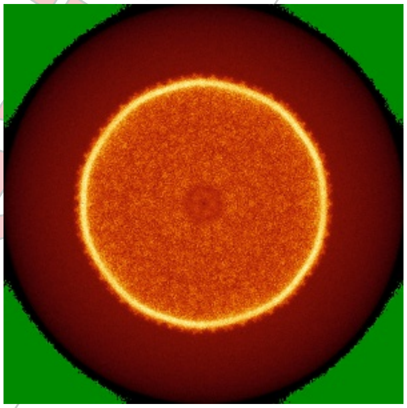
Z

6.4.2 Raw map

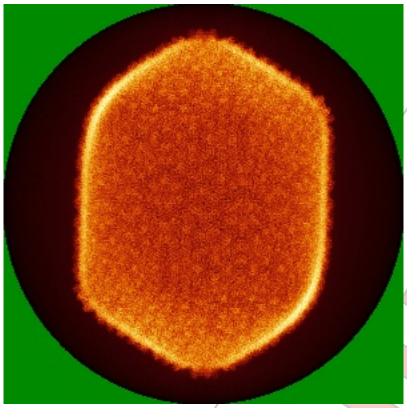
X

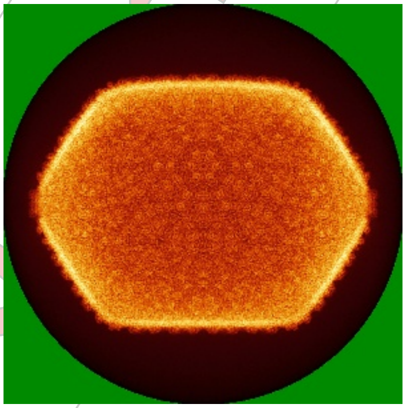
Y

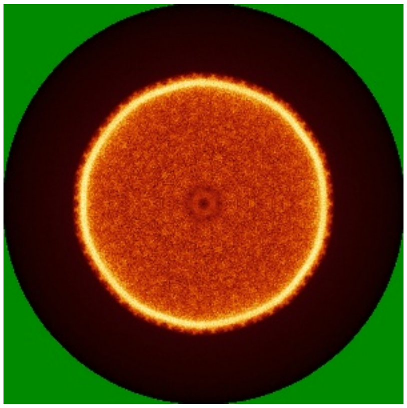
Z

The images above show the map standard deviation projections with false color in three orthogonal directions. Minimum values are shown in green, max in blue, and dark to light orange shades represent small to large values respectively.

For Manuscript Review

6.5 Orthogonal surface views (i)

6.5.1 Primary map

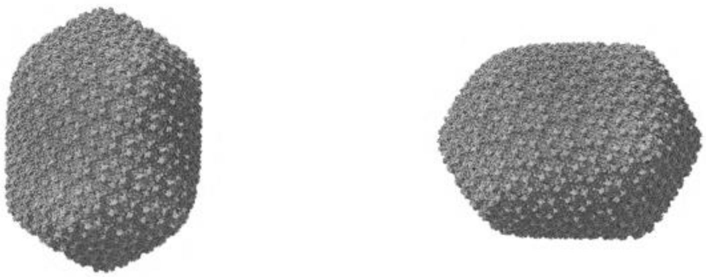

X Y Z

The images above show the 3D surface view of the map at the recommended contour level 0.014. These images, in conjunction with the slice images, may facilitate assessment of whether an appropriate contour level has been provided.

6.5.2 Raw map

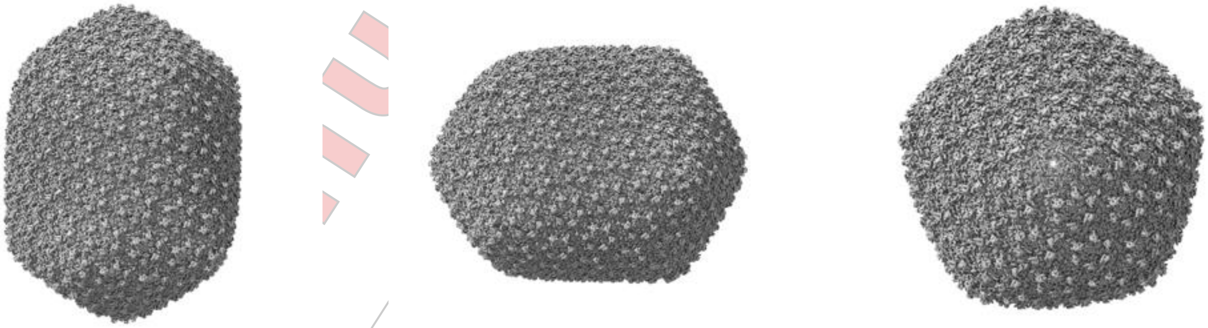

X Y Z

These images show the 3D surface of the raw map. The raw map's contour level was selected so that its surface encloses the same volume as the primary map does at its recommended contour level.

For Manuscript Review

6.6 Mask visualisation (i)

This section shows the 3D surface view of the primary map at 50% transparency overlaid with the specified mask at 0% transparency

A mask typically either:

- Encompasses the whole structure
- Separates out a domain, a functional unit, a monomer or an area of interest from a larger structure

6.6.1 D_1000273196_em-mask-volume_P1.map.V3 (i)

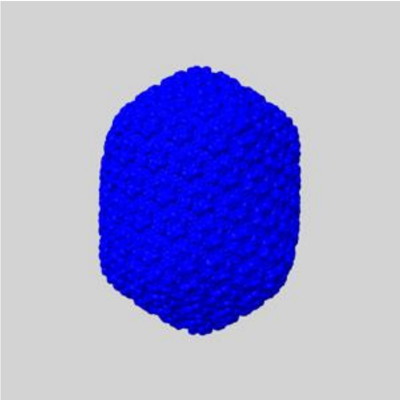

X

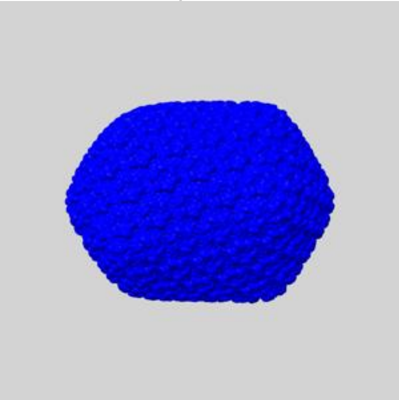

Y

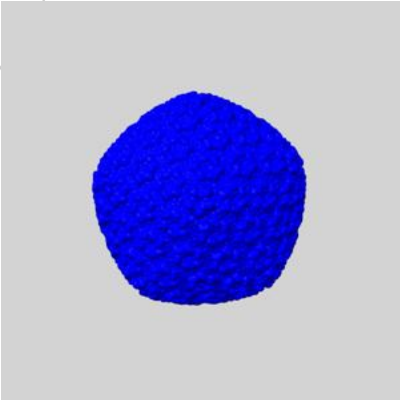

Z

For Manuscript Review

7 Map analysis (i)

This section contains the results of statistical analysis of the map.

7.1 Map-value distribution (i)

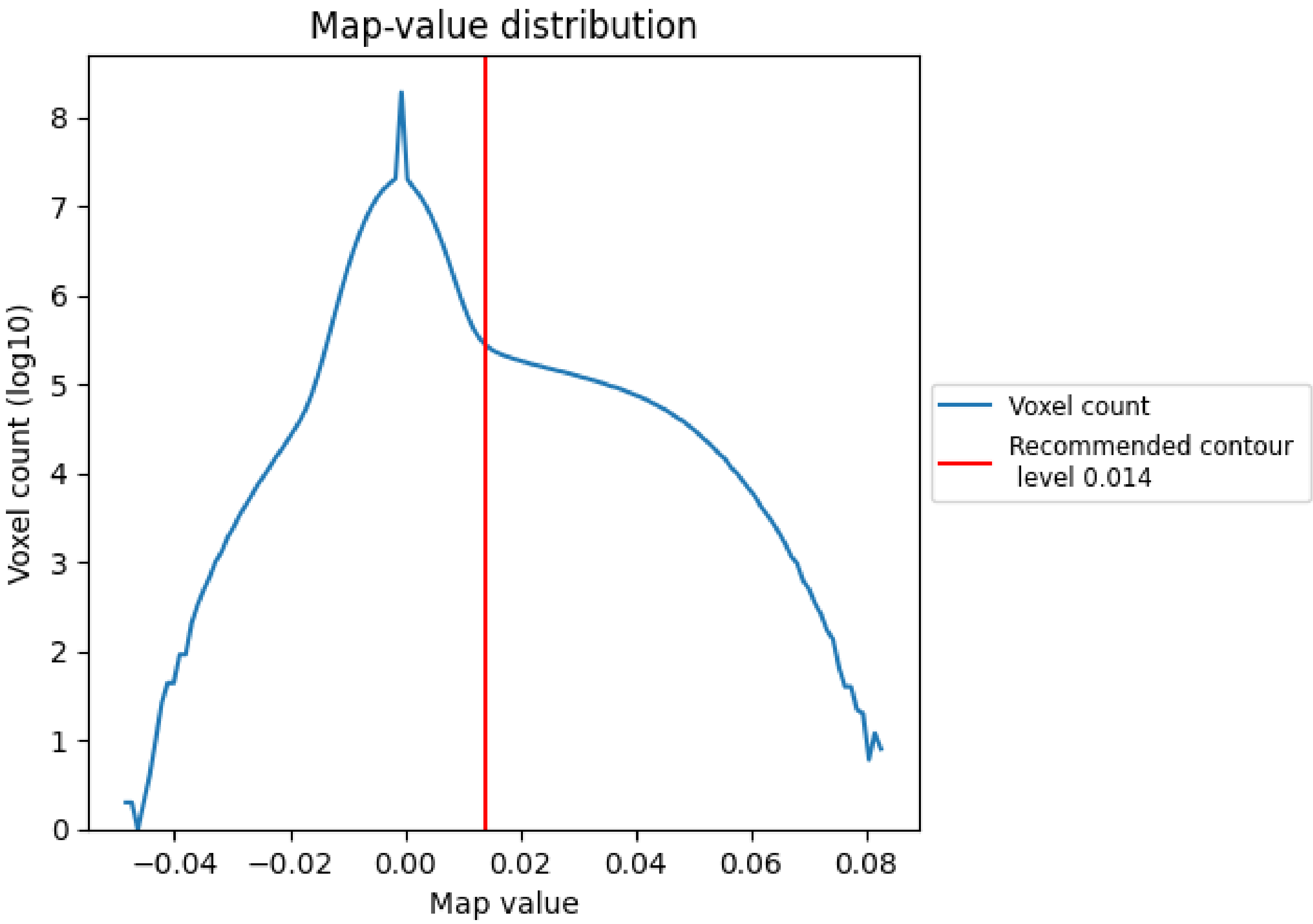

The map-value distribution is plotted in 128 intervals along the x-axis. The y-axis is logarithmic. A spike in this graph at zero usually indicates that the volume has been masked.

For Manuscript Review

7.2 Volume estimate (i)

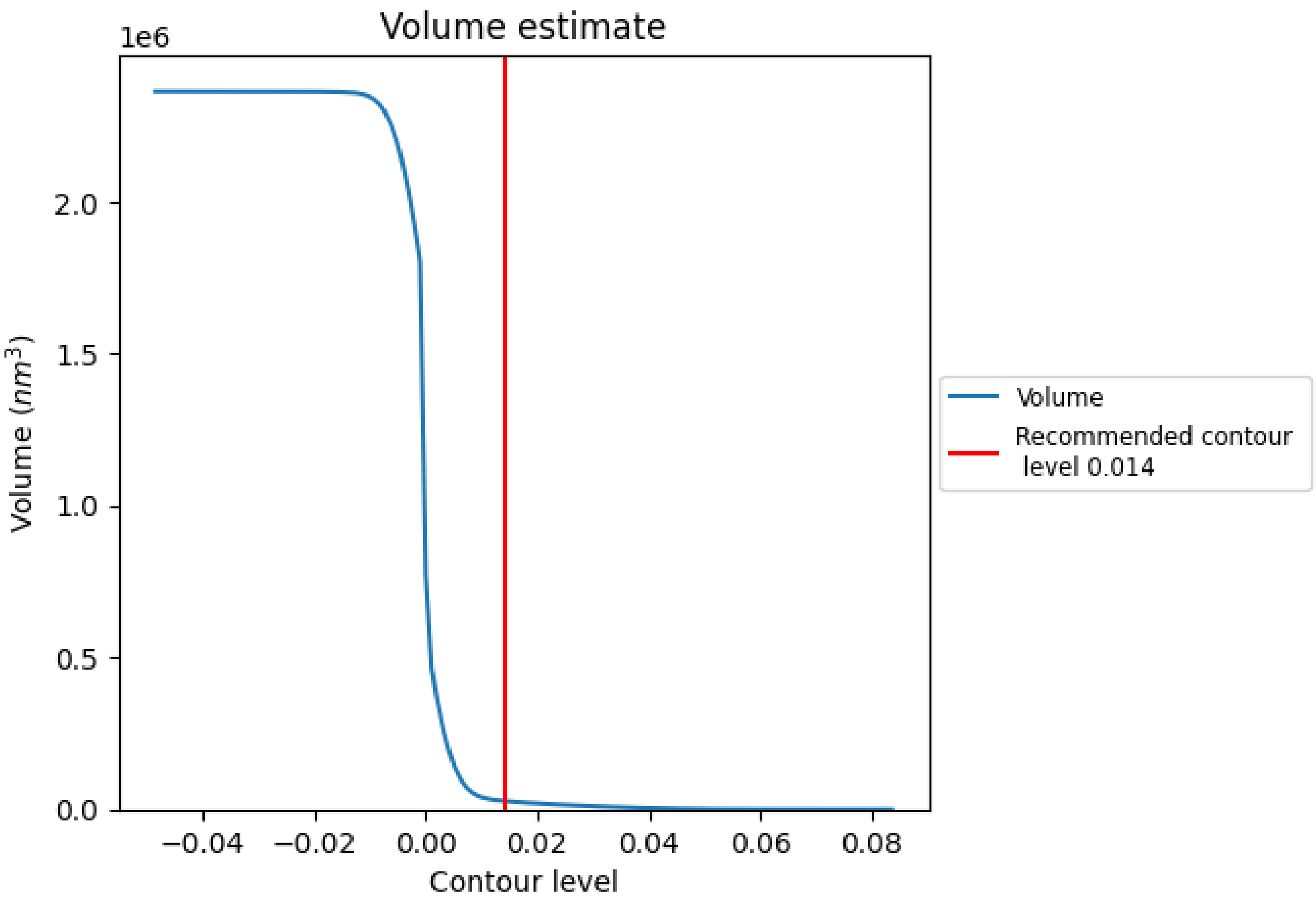

The volume at the recommended contour level is 28460 nm^3; this corresponds to an approximate mass of 25708 kDa.

The volume estimate graph shows how the enclosed volume varies with the contour level. The recommended contour level is shown as a vertical line and the intersection between the line and the curve gives the volume of the enclosed surface at the given level.

For Manuscript Review

7.3 Rotationally averaged power spectrum (i)

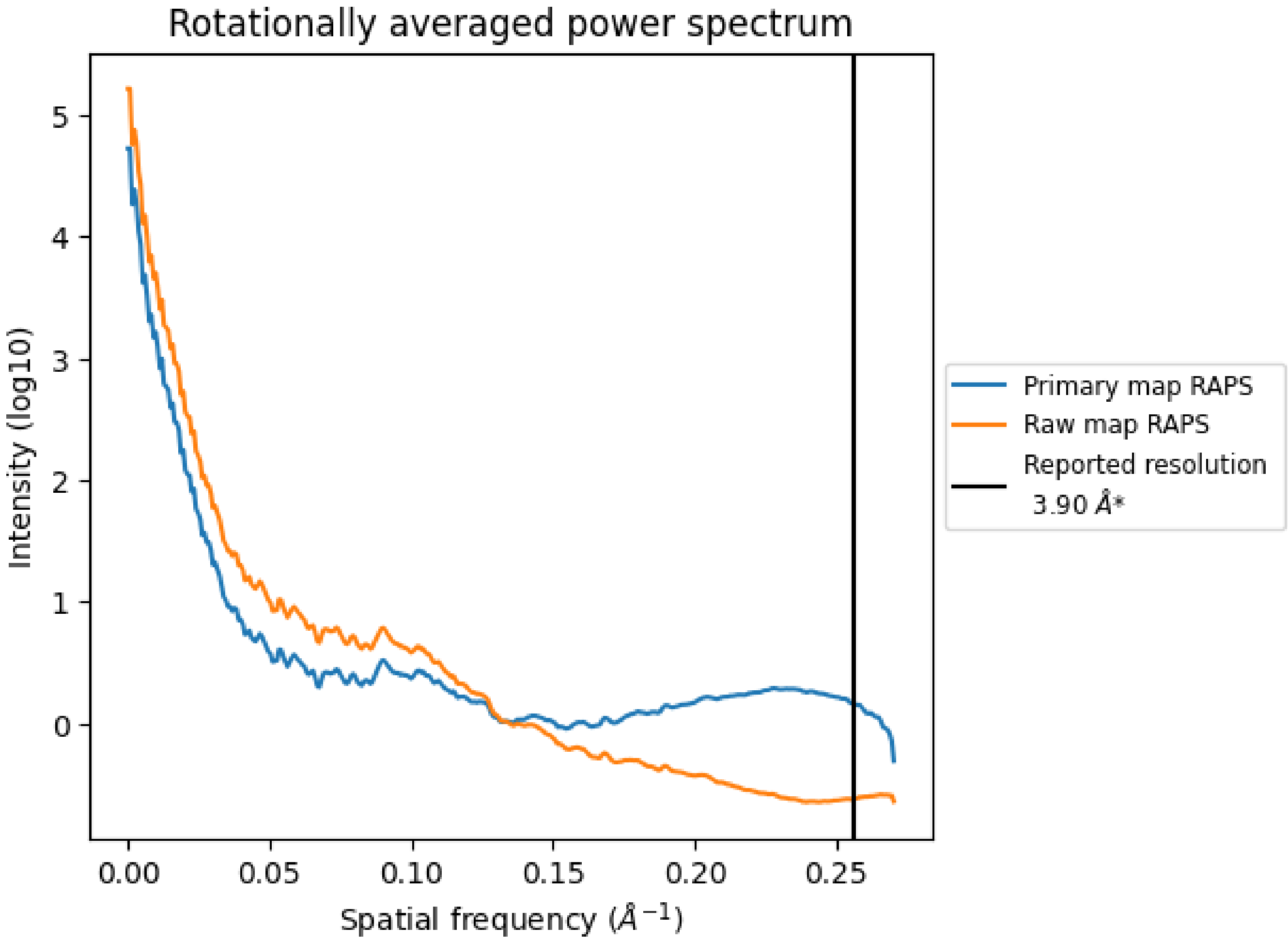

*Reported resolution corresponds to spatial frequency of 0.256 Å^{-1}

For Manuscript Review

8 Fourier-Shell correlation (i)

Fourier-Shell Correlation (FSC) is the most commonly used method to estimate the resolution of single-particle and subtomogram-averaged maps. The shape of the curve depends on the imposed symmetry, mask and whether or not the two 3D reconstructions used were processed from a common reference. The reported resolution is shown as a black line. A curve is displayed for the half-bit criterion in addition to lines showing the 0.143 gold standard cut-off and 0.5 cut-off.

8.1 FSC (i)

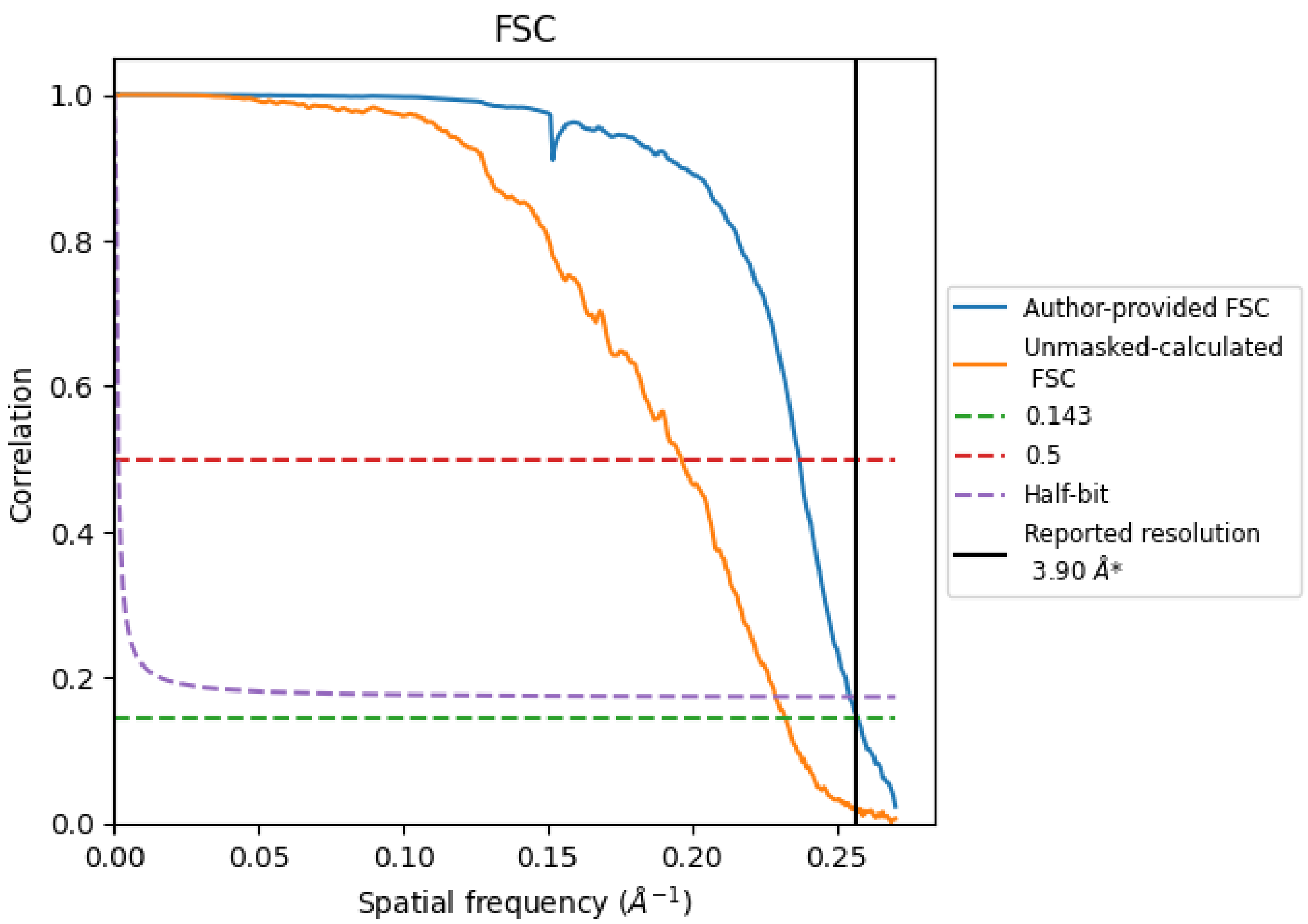

*Reported resolution corresponds to spatial frequency of 0.256 Å^{-1}

For Manuscript Review

8.2 Resolution estimates (i)

Resolution estimate (Å)	Estimation criterion (FSC cut-off)		
	0.143	0.5	Half-bit
Reported by author	3.90	-	-
Author-provided FSC curve	3.89	4.22	3.93
Unmasked-calculated*	4.31	5.09	4.37

*Resolution estimate based on FSC curve calculated by comparison of deposited half-maps. The value from deposited half-maps intersecting FSC 0.143 CUT-OFF 4.31 differs from the reported value 3.9 by more than 10 %

For Manuscript Review

9 Map-model fit (i)

This section contains information regarding the fit between EMDB map EMD-40228 and PDB model 8GMO. Per-residue inclusion information can be found in section 3 on page 32.

9.1 Map-model overlays

9.1.1 Map-model overlay (i)

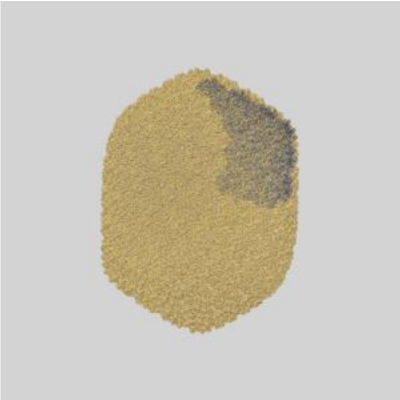
X

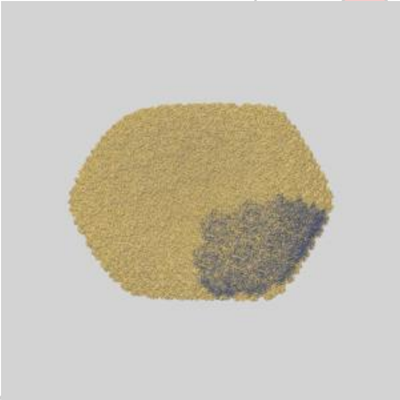
Y

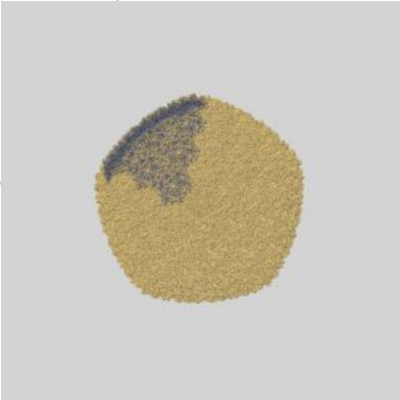
Z

9.1.2 Map-model assembly overlay (i)

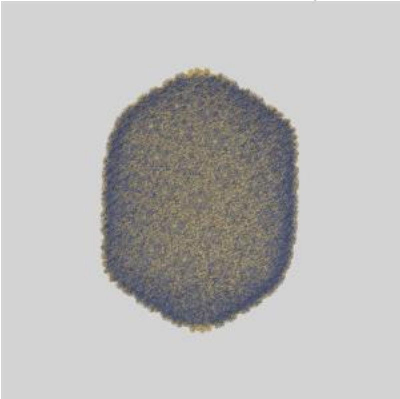
X

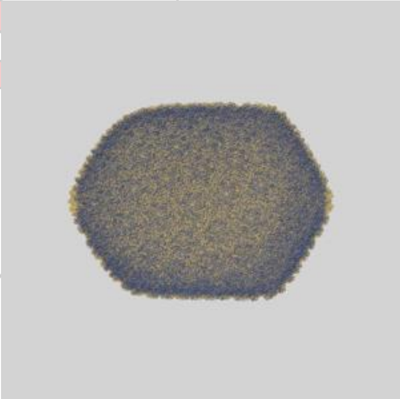
Y

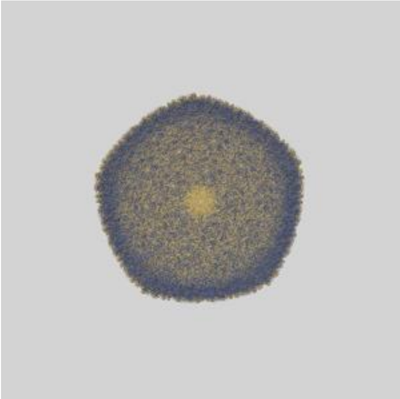
Z

The images above show the 3D surface view of the map at the recommended contour level 0.014 at 50% transparency in yellow overlaid with a ribbon representation of the model coloured in blue. These images allow for the visual assessment of the quality of fit between the atomic model and the map.

For Manuscript Review

9.2 Q-score mapped to coordinate model (i)

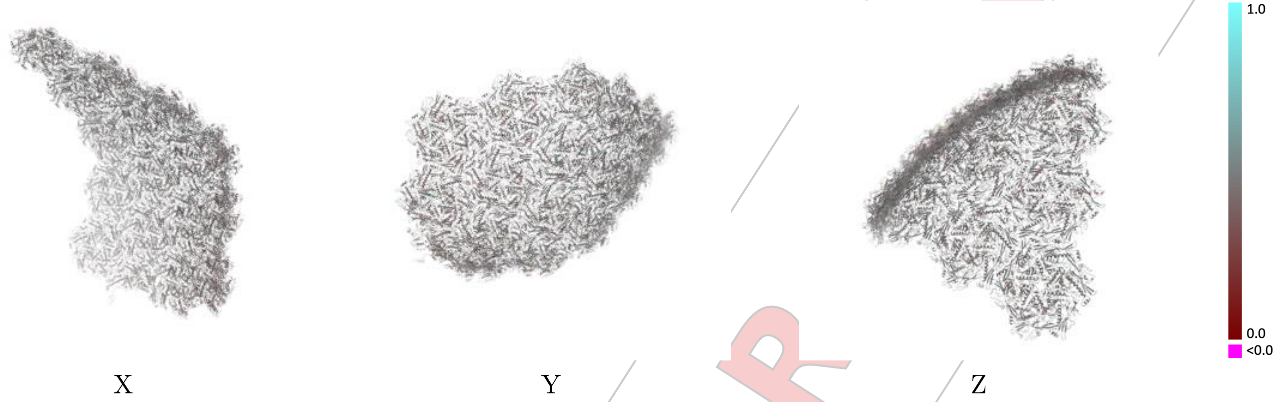

The images above show the model with each residue coloured according its Q-score. This shows their resolvability in the map with higher Q-score values reflecting better resolvability. Please note: Q-score is calculating the resolvability of atoms, and thus high values are only expected at resolutions at which atoms can be resolved. Low Q-score values may therefore be expected for many entries.

9.3 Atom inclusion mapped to coordinate model (i)

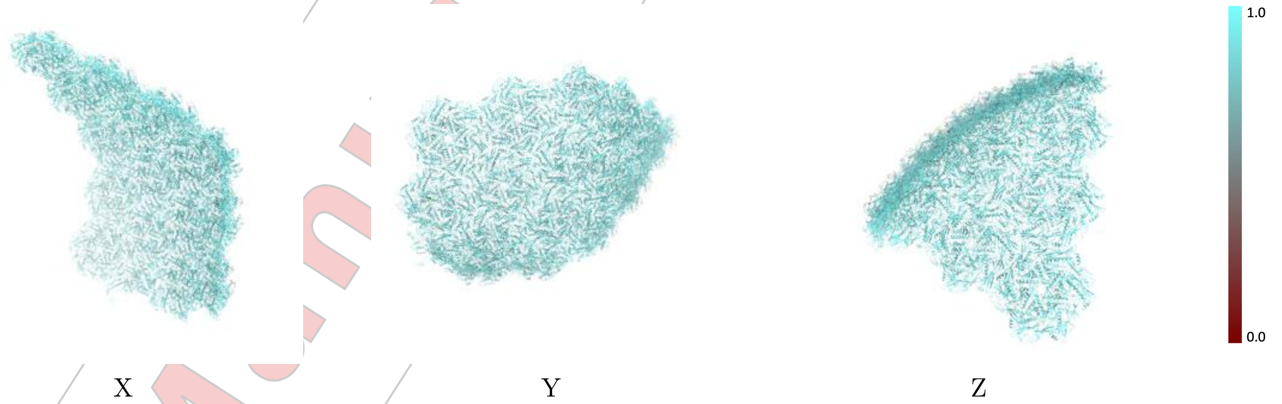

The images above show the model with each residue coloured according to its atom inclusion. This shows to what extent they are inside the map at the recommended contour level (0.014).

For Manuscript Review

9.4 Atom inclusion (i)

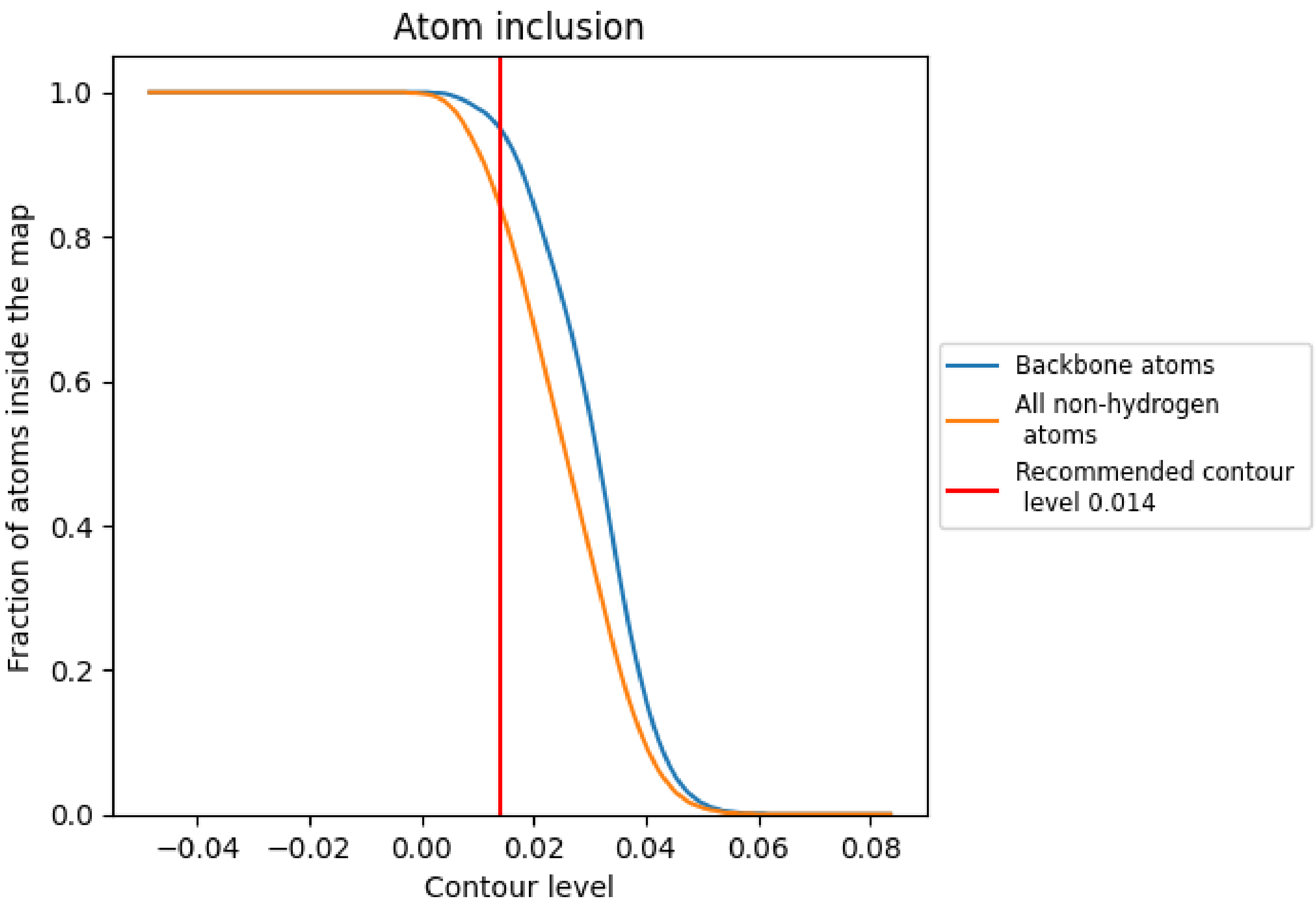

At the recommended contour level, 95% of all backbone atoms, 84% of all non-hydrogen atoms, are inside the map.

For Manuscript Review

9.5 Map-model fit summary (i)

The table lists the average atom inclusion at the recommended contour level (0.014) and Q-score for the entire model and for each chain.

Chain	Atom inclusion	Q-score
All	0.8430	0.4520
0	0.8340	0.4450
1	0.8150	0.4420
2	0.8060	0.4330
3	0.8040	0.4330
4	0.8170	0.4380
5	0.8180	0.4310
6	0.8380	0.4530
7	0.8260	0.4400
8	0.8270	0.4430
9	0.8260	0.4420
A	0.8280	0.4520
AA	0.8370	0.4430
AB	0.8290	0.4390
AC	0.8290	0.4440
AD	0.8600	0.4630
AE	0.8600	0.4600
AF	0.8730	0.4630
AG	0.8650	0.4570
AH	0.8670	0.4680
AI	0.8710	0.4690
AJ	0.8540	0.4570
AK	0.8660	0.4670
AL	0.8680	0.4690
AM	0.8610	0.4560
AN	0.8510	0.4520
AO	0.8650	0.4650
AP	0.8610	0.4620
AQ	0.8550	0.4620
AR	0.8610	0.4660
AS	0.8630	0.4610
AT	0.8640	0.4560
AU	0.8600	0.4600
AV	0.8670	0.4600
AW	0.8670	0.4620

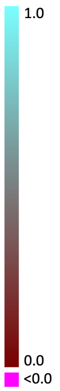

Continued on next page...

For Manuscript Review

Continued from previous page...

Chain	Atom inclusion	Q-score
AX	0.8670	0.4600
AY	0.8590	0.4570
AZ	0.8630	0.4620
Aa	0.8690	0.4600
Ab	0.8600	0.4650
Ac	0.8550	0.4650
Ad	0.8610	0.4620
Ae	0.8620	0.4640
Af	0.8620	0.4590
Ag	0.8580	0.4590
Ah	0.8590	0.4620
Ai	0.8640	0.4610
Aj	0.8620	0.4620
Ak	0.8550	0.4580
Al	0.8580	0.4610
Am	0.8560	0.4620
B	0.8320	0.4540
C	0.8350	0.4480
D	0.8390	0.4530
G	0.8190	0.4370
H	0.8230	0.4380
J	0.8200	0.4350
K	0.8520	0.4620
L	0.8500	0.4580
M	0.8440	0.4560
N	0.8480	0.4580
O	0.8370	0.4530
P	0.8390	0.4430
Q	0.8240	0.4430
R	0.8280	0.4430
S	0.8310	0.4410
T	0.8280	0.4350
U	0.8520	0.4610
V	0.8460	0.4560
W	0.8520	0.4610
X	0.8480	0.4570
Y	0.8410	0.4500
Z	0.8460	0.4570
a	0.8380	0.4530
b	0.8460	0.4600
c	0.8410	0.4490
d	0.8550	0.4600

Continued on next page...

For Manuscript Review

Continued from previous page...

Chain	Atom inclusion	Q-score
e	0.8380	0.4550
f	0.8310	0.4450
g	0.8410	0.4530
h	0.8450	0.4530
i	0.8570	0.4600
j	0.8490	0.4610
k	0.8180	0.4470
l	0.8390	0.4520
m	0.8370	0.4500
n	0.8230	0.4420
o	0.8390	0.4500
p	0.8180	0.4290
q	0.8110	0.4340
r	0.8050	0.4350
s	0.8170	0.4330
t	0.8050	0.4300
u	0.8150	0.4310
v	0.8340	0.4470
w	0.8390	0.4560
x	0.8350	0.4450
y	0.8380	0.4490
z	0.8160	0.4340

For Manuscript Review
