# Supplementary material for: Design of bacteriophage T4-based artificial viral vectors for human genome remodeling
Source: Nat Commun. 2023 May 30;14:2928. doi: 10.1038/s41467-023-38364-1 (PMC10229621; doi:10.1038/s41467-023-38364-1)
Supplement: Supplementary file 2 — Reporting Summary [file 41467_2023_38364_MOESM2_ESM.pdf]

nature portfolio

Corresponding author(s): Venigalla Rao
Last updated by author(s): Apr 16, 2023

Reporting Summary

Nature Portfolio wishes to improve the reproducibility of the work that we publish. This form provides structure for consistency and transparency in reporting. For further information on Nature Portfolio policies, see our Editorial Policies and the Editorial Policy Checklist.

Statistics

For all statistical analyses, confirm that the following items are present in the figure legend, table legend, main text, or Methods section.

n/a	Confirmed	
☐	☒	The exact sample size (*n*) for each experimental group/condition, given as a discrete number and unit of measurement
☐	☒	A statement on whether measurements were taken from distinct samples or whether the same sample was measured repeatedly
☐	☒	The statistical test(s) used AND whether they are one- or two-sided<br>*Only common tests should be described solely by name; describe more complex techniques in the Methods section.*
☒	☐	A description of all covariates tested
☒	☐	A description of any assumptions or corrections, such as tests of normality and adjustment for multiple comparisons
☐	☒	A full description of the statistical parameters including central tendency (e.g. means) or other basic estimates (e.g. regression coefficient) AND variation (e.g. standard deviation) or associated estimates of uncertainty (e.g. confidence intervals)
☐	☒	For null hypothesis testing, the test statistic (e.g. F, t, r) with confidence intervals, effect sizes, degrees of freedom and P value noted<br>*Give P values as exact values whenever suitable.*
☒	☐	For Bayesian analysis, information on the choice of priors and Markov chain Monte Carlo settings
☒	☐	For hierarchical and complex designs, identification of the appropriate level for tests and full reporting of outcomes
☒	☐	Estimates of effect sizes (e.g. Cohen's *d*, Pearson's *r*), indicating how they were calculated

Our web collection on statistics for biologists contains articles on many of the points above.

Software and code

Policy information about availability of computer code

Data collection	ZEN Lite 2012;<br>ChemiDoc™ Gel Imaging System;<br>EPU 3 Software
Data analysis	GraphPad Prism9 was used for data analysis and plotting.<br>Photoshop CS6 Extended for figure preparation.<br>Chimera 1.14 was used for protein structure analysis and modeling.<br>ImageJ 1.8.0_172 was used for band intensity analysis.<br>BioRender was used for schematic figures.<br>MotionCor 2 program.<br>CTFFind 4 program.<br>Relion 4 software package.<br>Coot program.<br>Phenix.real_space_refine program.

For manuscripts utilizing custom algorithms or software that are central to the research but not yet described in published literature, software must be made available to editors and reviewers. We strongly encourage code deposition in a community repository (e.g. GitHub). See the Nature Portfolio guidelines for submitting code & software for further information.

Data

Policy information about availability of data

All manuscripts must include a data availability statement. This statement should provide the following information, where applicable:

- Accession codes, unique identifiers, or web links for publicly available datasets
- A description of any restrictions on data availability
- For clinical datasets or third party data, please ensure that the statement adheres to our policy

All data supporting the findings of this study are included in the manuscript and its Supplementary Information files. The cryo-EM reconstruction of 9DE-T4 capsid generated in this study has been deposited in the Electron Microscopy Data Bank under the accession code EMD-40228 and in the Protein Data Bank under the accession number PDB: 8GMO. They are scheduled to be released upon publication of this paper. Source data are provided with this paper.

Human research participants

Policy information about studies involving human research participants and Sex and Gender in Research.

Reporting on sex and gender	N/A
Population characteristics	N/A
Recruitment	N/A
Ethics oversight	N/A

Note that full information on the approval of the study protocol must also be provided in the manuscript.

Field-specific reporting

Please select the one below that is the best fit for your research. If you are not sure, read the appropriate sections before making your selection.

[x] Life sciences [ ] Behavioural & social sciences [ ] Ecological, evolutionary & environmental sciences

For a reference copy of the document with all sections, see nature.com/documents/nr-reporting-summary-flat.pdf

Life sciences study design

All studies must disclose on these points even when the disclosure is negative.

Sample size	We did not use statistical methods to predetermine the sample sizes. Sample sizes were chosen based in the minimum number of independent experiments that allow statistical evaluation (n=3). The measurements were taken from distinct or independent biological samples (n=3-5 per individual group). The size numbers were indicated in each figure legend.
Data exclusions	No data were excluded.
Replication	All experiments were repeated at least three times with reproducible results.
Randomization	Cells or transduction materials were randomized into different groups.
Blinding	No blinding was performed in this study because the measurements in this study are objective and does not depend on the researcher's interpretation or bias.

Reporting for specific materials, systems and methods

We require information from authors about some types of materials, experimental systems and methods used in many studies. Here, indicate whether each material, system or method listed is relevant to your study. If you are not sure if a list item applies to your research, read the appropriate section before selecting a response.

Materials & experimental systems

n/a	Involved in the study
	☒ Antibodies
	☒ Eukaryotic cell lines
☒	☐ Palaeontology and archaeology
☒	☐ Animals and other organisms
☒	☐ Clinical data
☒	☐ Dual use research of concern

Methods

n/a	Involved in the study
☒	☐ ChIP-seq
☒	☐ Flow cytometry
☒	☐ MRI-based neuroimaging

Antibodies

Antibodies used

Anti-firefly luciferase antibody (1/500 dilution); Abcam; Cat#ab21176.
AlexaFluor488 goat-anti-rabbit IgG H&L (1/500 dilution); Abcam; Cat# ab150077.
Goat anti-mouse IgG-HRP antibody (1/50,000 dilution for ELISA and 1/10,000 dilution for WB); Abcam; Cat#ab6789.
Anti-tubulin antibody, GTU-88 clone (1/5,000 dilution); Sigma-Aldrich; Cat#T6557.
Anti-GFP antibody (1/2,000 dilution); Invitrogen; Cat#A11121.
Anti-Dystrophin antibody (1/100 dilution); Abcam; Cat#ab15277.
Anti-His6 antibody, HIS.H8 clone (1/2,000 dilution); Thermo Scientific; Cat#MA1-21315.
Goat anti-rabbit IgG-HRP antibody (1/10,000 dilution); Abcam; Cat#ab6721.
Goat anti-human IgG-HRP antibody (1/5,000 dilution); Abcam; Cat#ab6858.
HIV CH58 antibody, ARP12550 (1 mg/mL); the National Institutes of Health AIDS Reagent Program, Division of AIDS, NIAID. Cat#12550.

Validation

All the validation data of antibodies are available on the manufacturer's website.
Anti-firefly luciferase antibody; Abcam; Cat#ab21176.
https://www.abcam.com/firefly-luciferase-antibody-ab21176.html
AlexaFluor488 goat-anti-rabbit IgG H&L; Abcam; Cat# ab150077.
https://www.abcam.com/goat-rabbit-igg-hl-alexa-fluor-488-ab150077.html
Goat anti-mouse IgG-HRP antibody; Abcam; Cat#ab6789.
https://www.abcam.com/goat-mouse-igg-hl-hrp-ab6789.html
Anti-tubulin antibody; Sigma-Aldrich; Cat#T6557.
https://www.sigmaaldrich.com/US/en/product/sigma/t6557
Anti-GFP antibody; Invitrogen; Cat#A11121.
https://www.thermofisher.com/antibody/product/GFP-Antibody-Polyclonal/A-11122
Anti-Dystrophin antibody; Abcam; Cat#ab15277.
https://www.abcam.com/dystrophin-antibody-ab15277.html
Anti-His6 antibody; Thermo Scientific; Cat#MA1-21315.
https://www.thermofisher.com/antibody/product/6x-His-Tag-Antibody-clone-HIS-H8-Monoclonal/MA1-21315
Goat anti-rabbit IgG-HRP antibody; Abcam; Cat#ab6721.
https://www.abcam.com/goat-rabbit-igg-hl-hrp-ab6721.html
Goat anti-human IgG-HRP antibody; Abcam; Cat#ab6858.
https://www.abcam.com/goat-human-igg-hl-hrp-ab6858.html
HIV CH58 antibody; the National Institutes of Health AIDS Reagent Program, Division of AIDS, NIAID. Cat#12550.
https://www.hivreagentprogram.org/Catalog/HRPMonoclonalAntibodies/ARP-12550.aspx

Eukaryotic cell lines

Policy information about cell lines and Sex and Gender in Research

Cell line source(s)	HEK293T cells from ATCC
Authentication	Authentication by ATCC. Cell morphology and behavior was consistent with expectations.
Mycoplasma contamination	The cell line was tested to be negative for Mycoplasma.
Commonly misidentified lines (See ICLAC register)	No commonly misidentified cell lines were used in this study.
