# Supplementary material for: Design of bacteriophage T4-based artificial viral vectors for human genome remodeling
Source: Nat Commun. 2023 May 30;14:2928. doi: 10.1038/s41467-023-38364-1 (PMC10229621; doi:10.1038/s41467-023-38364-1)
Supplement: Supplementary file 4 — Description of Additional Supplementary Files [file 41467_2023_38364_MOESM4_ESM.pdf]

Title: Supplemental Movie 1

Description: Sequential Assembly of Bacteriophage T4 Artificial Viral Vectors (T4-AVV).
