# Supplementary material for: Design of bacteriophage T4-based artificial viral vectors for human genome remodeling
Source: Nat Commun. 2023 May 30;14:2928. doi: 10.1038/s41467-023-38364-1 (PMC10229621; doi:10.1038/s41467-023-38364-1)
Supplement: Supplementary file 5 — Supplementary Movie 1 [file 41467_2023_38364_MOESM5_ESM.pptx]

## Slide 1
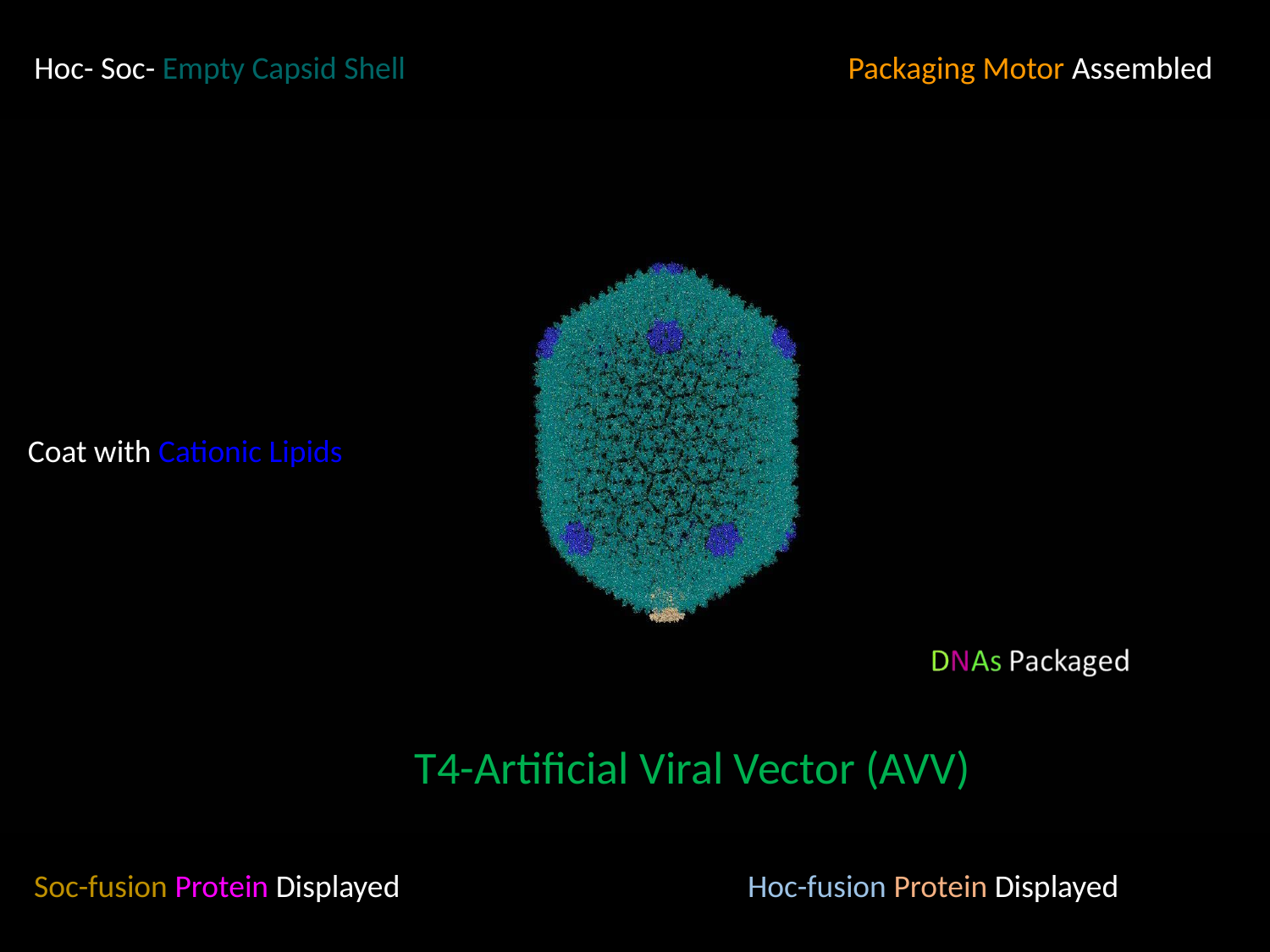

Hoc- Soc- Empty Capsid Shell
Packaging Motor Assembled
Coat with Cationic Lipids
T4-Artificial Viral Vector (AVV)
Soc-fusion Protein Displayed
Hoc-fusion Protein Displayed
